# Lithic technological responses to Late Pleistocene glacial cycling at Pinnacle Point Site 5-6, South Africa

**DOI:** 10.1371/journal.pone.0174051

**Published:** 2017-03-29

**Authors:** Jayne Wilkins, Kyle S. Brown, Simen Oestmo, Telmo Pereira, Kathryn L. Ranhorn, Benjamin J. Schoville, Curtis W. Marean

**Affiliations:** 1 Human Evolution Research Institute, Department of Archaeology, University of Cape Town, Rondebosch, Private Bag, South Africa; 2 Centre for Coastal Paleoscience, Nelson Mandela Metropolitan University, Port Elizabeth, Eastern Cape, South Africa; 3 Institute of Human Origins, School of Human Evolution and Social Change, Arizona State University, Tempe, AZ, United States of America; 4 Interdisciplinary Center for Archaeology and Evolution of Human Behavior, Faculdade das Ciências Humanas e Sociais, Universidade do Algarve, Campus Gambelas, Faro, Portugal; 5 Center for the Advanced Study of Human Paleobiology, George Washington University, Washington, DC, United States of America; Max Planck Institute for the Science of Human History, GERMANY

## Abstract

There are multiple hypotheses for human responses to glacial cycling in the Late Pleistocene, including changes in population size, interconnectedness, and mobility. Lithic technological analysis informs us of human responses to environmental change because lithic assemblage characteristics are a reflection of raw material transport, reduction, and discard behaviors that depend on hunter-gatherer social and economic decisions. Pinnacle Point Site 5–6 (PP5-6), Western Cape, South Africa is an ideal locality for examining the influence of glacial cycling on early modern human behaviors because it preserves a long sequence spanning marine isotope stages (MIS) 5, 4, and 3 and is associated with robust records of paleoenvironmental change. The analysis presented here addresses the question, *what*, *if any*, *lithic assemblage traits at PP5-6 represent changing behavioral responses to the MIS 5-4-3 interglacial-glacial cycle?* It statistically evaluates changes in 93 traits with no *a priori* assumptions about which traits may significantly associate with MIS. In contrast to other studies that claim that there is little relationship between broad-scale patterns of climate change and lithic technology, we identified the following characteristics that are associated with MIS 4: increased use of quartz, increased evidence for outcrop sources of quartzite and silcrete, increased evidence for earlier stages of reduction in silcrete, evidence for increased flaking efficiency in all raw material types, and changes in tool types and function for silcrete. Based on these results, we suggest that foragers responded to MIS 4 glacial environmental conditions at PP5-6 with increased population or group sizes, ‘place provisioning’, longer and/or more intense site occupations, and decreased residential mobility. Several other traits, including silcrete frequency, do not exhibit an association with MIS. Backed pieces, once they appear in the PP5-6 record during MIS 4, persist through MIS 3. Changing paleoenvironments explain some, but not all temporal technological variability at PP5-6.

## Introduction

Humans are unique for their extreme behavioral plasticity and their exaggerated dependence on social learning [1–7]. Given that the modern human lineage originates in Africa, it then follows that the roots for these traits have their origins in Africa, but the origin point or points are still unknown. Humans with modern physical characteristics first appeared in East Africa ~190–150 ka [8–10]. Evidence for behaviors linked to modern behavioral capacities–symbolism, complex tools, sophisticated foraging strategies–first begin appearing in South Africa at about the same time ~ 160 ka [11, 12], but become more common and widespread starting ~100 ka [13–32].

The latter part of the evolution of our species witnessed three major glacial cycles; marine isotope stages (MIS) 6 (191 ka-130 ka), MIS 4 (74 or 71 ka-57 ka), and MIS 2 (29 ka-14 ka)[33, 34]. While the precise environmental impacts of these glacial cycles are still relatively unknown, it is safe to say that they would have had some significant impacts on the distribution of resources upon which humans relied. It has been argued that humans adapted to climate change by changing their mobility strategies and the degree to which they invested in inter-group trade and exchange [35], and that fluctuating conditions through the Plio-Pleistocene may have selected for human traits that permit flexible behavioral responses to new environments [36]. Glacial pulses in the Pleistocene may have also isolated early human populations, explaining evidence for low genetic diversity in modern human populations [37] and perhaps promoted territoriality and hyper-prosociality [38, 39]. On the south coast of South Africa, there is a research program in place to specifically model these impacts [40, 41].

There is some consensus that behavioral variability through the Late Pleistocene in South Africa is at least in part explained by technological adaptation to changing environmental conditions. However, as is detailed below, even at the largest scale of MIS and glacial/interglacial cycles, there is a huge degree of disagreement on how exactly early modern humans responded to these changing environmental conditions, even on a broad, time-averaged scale. The disagreement persists largely because few Late Pleistocene archaeological sites are connected to robust and regional paleoenvironmental records, and there are few sites that preserve long sequences across multiple MIS. Because it is linked to a robust paleoenvironmental record and a multidisciplinary program focused on characterizing past resource availabilities [40, 42–50], Pinnacle Point Site 5–6 (PP5-6) presents an ideal case study for examining the nature of early modern human behavioral responses to glacial cycling in the Late Pleistocene.

Lithic artifacts are a reflection of cognition, cultural patterns, technological needs, and economic decision making, and they provide information on how past populations moved across the landscape, chose stone raw material sources, manufactured tool blanks and tools, and discarded debris. Informed by a combination of experimental and ethnographic research, lithic technology is used to make higher-level interpretations about human behaviors such as interaction (e.g. [35, 51–53]), mobility (e.g. [35, 54]), cultural learning and innovation (e.g. [23, 55, 56]), and group/population size (e.g. [35, 57]).

This paper investigates how early modern humans responded to glacial cycling during the Late Pleistocene at PP5-6, South Coast, South Africa. It examines the lithic technological sequence from the site at the scale of MIS. Based on more than 65 optically stimulated luminescence (OSL) age estimates, PP5-6 provides a high-resolution, nearly continuous record of early modern human behavior between ~89 and ~51 ka [22, 43], spanning two interglacials (MIS 5 and MIS 3) and one glacial (MIS 4). The question addressed by this analysis is: *what*, *if any*, *lithic assemblage traits at PP5-6 represent changing behavioral responses to the MIS 5-4-3 interglacial-glacial cycle?*

## Background

### The effect of Late Pleistocene glacial cycling on temperature and rainfall

Researchers generally associate glacial periods in Africa with colder, drier climates and interglacial periods, such as today, with warmer, wetter climates (e.g., [58]). The sedimentary and speleothem records at some interior sites are in line with this generalization [59, 60]. These patterns, however, varied across the continent [61] and for southern Africa [62]. Environmental indicators at many sites are consistent with cooler and wetter conditions during the Last Glacial Maximum [63–65] and MIS 4 [66]. For the Cape, there is disagreement on whether glacial phases were characterized by increased aridity or increased moisture, and the lack of evidence for major changes in precipitation suggests that changes in overall moisture availability may have been muted [67], while changes in rainfall seasonality may have been significant [42].

In addition to influencing the amount of precipitation, it has been argued that glacial cycling influenced the position of the winter rainfall belt, changing for many regions across South Africa the relative inputs of winter and summer rainfall [68]. The exact impacts are still unclear [67]; the winter rainfall regime in South Africa generally supports C_3_ pathway plants, while the summer and bimodal rainfall regime supports more C_4_ pathway grasses. At Crevice Cave on the South Coast near Pinnacle Point, speleothem isotopes track this shifting rainfall regime, showing relatively more input from the summer rainfall regime during MIS 4 with a lessened summer rain input during MIS 5 [42]. This finding is inconsistent with general predictions that glacial phases will normally be characterized by expansions of the winter rainfall regime [69, 70]. For most parts of South Africa, whether glacial conditions were wetter or drier, and whether there was an increase or decrease in seasonal rainfall are not yet resolved, but there is consensus that the large-scale global changes in temperature resulted in differing rainfall conditions during MIS 5, 4, and 3.

### The effect of Late Pleistocene glacial cycling on food resource distribution

Changes in climate and vegetation due to glacial cycling would have affected the distribution of food resources for supporting a hunter-gatherer lifestyle across the landscape, and theory allows some basic predictions as to how this may have affected hunter-gatherer behavior. For example, Dyson-Hudson and Smith [71] emphasized the importance of resource predictability and density for understanding human territoriality (see also [72–74]). Harpending and Davis [75] have highlighted the relationship between resource predictability, density and mobility. Bleed [76] argues that the most important parameter for understanding hunting weapon design is predictability. Resource density will also set the limits on population size. In general, humans should adapt to predictable and densely-packed resources with elevated territoriality [71], decreased mobility [75], and ‘over-designed’ reliable hunting weapons [76], and their populations will increase. Building on the above, Ambrose and Lorenz [35] hypothesized the resource distribution during MIS 5 in South Africa (interglacial) was predictable and dense, during MIS 4 (glacial) resource distribution was predictable and scarce, and that during MIS 2 (glacial), resource distribution was unpredictable and dense. Resource structure for MIS 3 (interglacial) remains unknown. These generalizations were based on a combination of environmental and archaeological data from sites such as Border Cave, Boomplaas Cave, Montagu Cave, Nelson Bay Cave, and Klasies River, and on the following characterizations: (1) resources in savanna and grassland biomes with large herds of migratory ungulates are unpredictable and dense, (2) coastal resources such as shellfish are predictable and dense, (3) scrub or fynbos is characterized by predictable, but scarce resources in the form of small mammals with an even dispersion. The characterization for MIS 4 is partly based on evidence for scrub-adapted species in the archaeological record during this time [35].

In contrast, Marean [38, 77] suggests that resource predictability and density across much of the West and South Coast changed little in response to glacial cycling. The primary driver of Pleistocene change was the expansion and contraction of the Paleo-Agulhas Plain, which resulted in changes in the size and structure of resources based on that plain [67]. A key component of this model is that by the late Middle Pleistocene or early Upper Pleistocene humans had adopted a coastal adaptation on the south coast that relied to a large extent on shellfish resources which tend to be dense and predictable [38], and was supplemented by exploitation of the diverse geophyte plants and a migratory large mammal ecosystem on the Paleo-Agulhas Plain [49, 77, 78]. Because of the stable supply of predictable and dense resources, these regions may have been able to support human populations through glacial periods when other regions were not, however, a more robust investigation is required to hind cast changes in resource availabilities in response to glacial cycling [40].

### The effect of Late Pleistocene glacial cycling on human behaviors

There are many hypotheses about how Late Pleistocene humans in South Africa responded to changing resource availabilities due to glacial cycling and some hypotheses are contradictory to each other. To summarize what will be detailed below, the various human responses to glacial conditions in southern Africa that have been proposed include reduced population sizes (page 107 in [79] and page 163 in [80]), demographic expansions ([81], c.f. [82]), increased population and/or group sizes and longer and more intense site occupations [43], increased mobility [35, 54], decreased residential mobility [54], increased interconnectedness [35, 51, 83], decreased interconnectedness [84], increased resource intensification [15, 35, 85–87], and decreased territoriality [35, 79, 80].

Some researchers have focused on changes in mobility and settlement systems. Ambrose and Lorenz [35] suggest that foragers in southern Africa generally had higher mobility during glacials than during the current interglacial. Similarly, McCall and Thomas argue that the unique technological characteristics of two MSA industries that date to MIS 4—the Still Bay (SB) and Howiesons Poort (HP)—reflect changing mobility systems during a glacial period. It is argued by McCall and Thomas [54] that the SB reflects extreme mobility, and the HP differs from the SB in that it reflects a logistical mobility system that would have resulted in longer occupations at residential camps (i.e., reduced residential mobility). Consistent with the latter prediction for reduced residential mobility, but not necessarily a logistical mobility system, Karkanas et al. (2015) found there is sedimentological evidence for increased site occupation intensity during the MIS 4 glacial compared to the MIS 5 interglacial at PP5-6. At Klipdrift Shelter, Reynard et al. [88], found that an environmental shift toward open grasslands during the HP occupation corresponds with increased faunal density and trampling evidence suggesting increased occupation intensity [88].

Another potential response of humans to glacial cycling concerns interaction and inter-group connectedness. Mackay et al. [51] suggest that during MIS 4 (glacial), there was increased interconnectedness, or 'coalescence', in contrast to MIS 5 and 3 (interglacials), when human populations were more fragmented and isolated. In a related vein, Ambrose and Lorenz [35] suggest that there was increased information exchange during MIS 2 and 4 (glacials) compared to the current interglacial. Lowered sea-levels during glacials, which expose a continuous, unobstructed coastal plain that is accessible to the interior, may have facilitated these increased interactions [83].

Related to inter-group connectedness, innovation and symbolism have also been tied to MIS 4 glacial conditions. For example, it is argued that rapid cooling events in MIS 4 created selective pressures for the innovation and invention of new technologies such as bifacial points and backed pieces [89]. Glacial conditions may have changed resources distributions in such a way as to select for risk reduction behaviors in humans, such as technological overdesign (i.e., reliable technology [90]) and increased trade and exchange [35]. Increased information exchange and interaction between groups living in diverse environments helps buffer the effects of environmental change and scarce or unpredictable resources [91]. Interaction is facilitated by symbolic items–in MIS 4, potentially ochre, beads, engraved ostrich eggshell, weapon tips, rare raw material types [13, 15, 52, 53, 92]–and increased interaction fuels innovation–because it expands the number and reach of social learning opportunities [93]. Mellars [81] suggests new economic, social, and cognitive behaviors in MIS 4 were the impetus for a dramatic demographic expansion that ultimately resulted in the dispersal of modern humans into Eurasia and beyond. However, Wadley [94, 95] problematizes using similarities in lithic material culture to make interpretations about the flow of ideas, and the presence of what archaeologists consider symbolic items as evidence for cultural complexity.

In contrast, others argue that it was strong glacial pulses in the Pleistocene that likely isolated early human populations [77, 78, 84, 96], particularly during MIS 6 when genetic evidence suggests a bottleneck in the human lineage [97]. Isolated populations result in diversifying populations and cultures, and can lead to regional population extinctions. However, Blome et al. [61] show that changes in the frequency of archaeological sites is asynchronous with climate change, and argue that there is no support for population decline during MIS 4.

#### Lithic technological responses

Two technological industries in South Africa–the Still Bay (SB) and Howiesons Poort (HP)–date to MIS 4 [98–100]. The SB, characterized primarily by the presence of finely-made bifacial points, and the HP, characterized primarily by the presence of backed pieces and bladelets, have been presented as exceptional technological phases that flickered in and out of existence (e.g. [89, 93, 98, 101, 102]). Based on numerous and well-constrained age estimates at PP5-6, Brown et al. [22] have showed that backed pieces and small blade technology persisted for at least 11 ka starting 71 ka. Earlier dates for the HP at Diepkloof in the order of ~100 ka have also been produced, and are the focus of current debate [99, 103–105]. The post-HP MIS 3 assemblages [~58 ka, 98] at Klasies River lack backed pieces [106], but backed pieces are present in other early MIS 3 assemblages at sites such as Diepkloof Rockshelter [107], Rose Cottage Cave [108], and Sibudu Cave [109]. It has been suggested that, in general, Late Pleistocene glacial assemblages compared to interglacial assemblages exhibit more emphasis on blade flaking systems rather than radial flaking systems and more emphasis on backed artifacts or bifacial points rather than unifacial points or denticulates, [51]. However, backed pieces and bifacial points are not unique to one particular time period and have a long, but perhaps, punctuated, chronology in the South African MSA [110].

With regard to human behavioral responses to glacial cycling that are reflected specifically by lithic technology, the observations and interpretations are diverse. Some researchers propose that the lithic assemblages of MIS 4 are marked by inter-regional similarities, and MIS 5 and 3 by inter-regional heterogeneity [35, 51]. Based on the presence of large assemblages with evidence for onsite production, Mackay et al. [51] suggest that there is increased evidence for ‘place’ rather than individual provisioning during the HP. For the SB, small assemblages with emphasis on implement repair and maintenance are consistent with individual provisioning [51]. Comparisons to earlier and later time periods are not available because of limited data. The HP (glacial) phase at Diepkloof and Klein Kliphuis is characterized by an increased edge length to mass ratio compared to earlier and later periods, which is reflective of more conservative, more efficient flaking strategies [111].

One of the best documented shifts in lithic technology at South African sites is an increase in more ‘non-local’ (c.f. [112]) and/or fine-grained raw materials during glacial MIS 4 [35, 51, 113] and glacial MIS 2 [79, 90]. It is now known that in many cases, the “non-local” silcrete at MSA assemblages is actually local [112] and only appears different due to the effects of heat treatment [12]. At Klasies River, the frequency of silcrete and other fine-grained raw materials decreases in the MIS 3 (MSA III) occupation compared to the MIS 4 (HP) occupation [114]. At other sites, such as Diepkloof [107], Rose Cottage Cave [108], and Sibudu Cave [115], the raw material shift is less pronounced or absent.

The lithic characteristics described above have been variably linked to aspects of hunter-gatherer demography, mobility, and interaction, but there are inter-analyst differences in how the variability is interpreted. For example, the increased use of ‘non-local’ or fine-grained raw material has been interpreted as evidence for increased residential mobility [35, 54], decreased residential mobility [54], and increased interconnectedness [35, 51]. Related to the latter, other researchers have emphasized the non-functional, symbolic aspects of raw material choice [52, 53]. The raw material shift associated with MIS 4 has also been interpreted in a strictly cost-benefit framework, where the shift can be explained by changing costs in the procurement and production of silcrete versus quartzite [113, 116].

More recently researchers have been emphasizing the lack of a deterministic relationship between environment and lithic technology in the MSA. For example, Wurz [56], draws attention to the point that the apparent increase in temporal and spatial patterning during the SB and HP compared to earlier and later time periods could be in part a reflection of more intense research, and that those industries are best understood within the context of local historical trajectories of technological change rather than as responses to environmental change. This perspective of the MSA in general is supported by evidence for dynamic, short-term behavioral variability in the recently studied MIS 3 levels at Sibudu [117–119]. At Blombos Cave, the SB technocomplex is associated with warmer temperatures (based on shellfish types) and higher productivity (based on mammal assemblages), leading Langejans et al. [120] to argue that the SB technocomplex is not an adaption to harsh conditions or low environmental productivity as has previously been suggested. Clark [121] and Wadley [95] highlight a marked disconnect at Sibudu in the timing and nature of changes in the local environment relative to HP-post HP technological change and at the scale of the technocomplex. A lack of correlation between local environmental change and technological change at Blombos Cave and Klipdrift Shelter is used to argue that environment was not a significant driver of innovation, also at the scale of the technocomplex [122]. In their multi-site, macro-scale investigation of MSA variability, Kandel et al [123] argue that the Early MSA, SB, HP, and Late MSA are not associated with any particular environmental condition or conditions, and thus cultural change is not connected to environmental factors.

#### Mobility and lithic assemblage characteristics

Lithic assemblage characteristics have been used to establish how hunter-gatherer mobility changed through time. Mobility can be classified as residential or logistical [124], where the mobility strategy of a hunter-gatherer group is conceptualized as involving both kinds of mobility, but with different relative importance along a continuum [125–127]. Residential mobility describes the process of moving all members of a group from one residential location to another. Logistical mobility describes the process of a small sub-groups moving away from the residential site for short-term stays at separate camps. Often, groups with low residential mobility have high logistical mobility, because special task groups move resources back to residential camps, reducing the need to move the whole group. However, this is not always the case; some hunter-gatherer groups, such as many contact period California Indians, simultaneously exhibit low residential mobility and low logistical mobility, and can thus be described as relatively more sedentary then other hunter-gatherer groups [41]. This mobility strategy is normally associated with focused use of dense and predictable resources, such as marine resources. Therefore, mobility strategy must be characterized, not along a single residential-logistical continuum, but along two axes, with the second axis reflecting degree of sedentariness combined with a lack of logistical mobility [41].

PP5-6 permits a consideration of changing degrees of residential mobility and sedentariness. The protective nature and large space afforded by the rockshelter geologic structure would make the site an attractive locus of re-occupation. The high density of artifacts particularly compared to nearby open-air localities at Vleesbaai [128], low frequency of impact fractures [129], and range of tool edge damage patterning that represents a range of processing activities [130] suggest that PP5-6 served primarily as a residential site [124, 131] through much, but not necessarily all, of its occupation record. For that reason, duration of occupation at PP5-6 primarily informs us about degree of residential mobility. The resource structure and low seasonality of southern Africa would, in general, not promote high degrees of logistical mobility, but there were likely changes in the degree of residential mobility, or sedentariness, depending on the resource base [41].

Proxies for the duration of occupation at PP5-6 give an indication of how frequently early modern human hunter-gatherers made residential moves. The archaeological record indicates that many prehistoric North American groups shifted to more ‘expedient’ and ‘informal’ stone technologies (i.e., fewer prepared cores, fewer retouched pieces, less intense reduction) when they became more sedentary, and their residential mobility decreased [132]. This is because lithic raw material can be effectively abundant at base camps due to direct local availability, embedded procurement, or place provisioning. However, others have demonstrated that more ‘formal ‘ technologies that lengthen the use-life of cores and tools (i.e., more prepared cores, more retouched pieces, and/or more intense reduction) are produced by sedentary groups when good quality lithic raw materials are not locally abundant [131, 133]. When decreased residential mobility situates hunter-gatherer groups where there is a local scarcity of good quality lithic raw material, hunter-gatherers can respond by using more intense reduction strategies, represented by a higher blank to core ratio, smaller flakes and cores, and less cortex [134]. Depletion can also play a role in raw material availability; raw material will also be more efficiently and intensely reduced when raw materials near a site are continuously exploited because duration of occupation is longer, or group/population size is larger [135]. How we interpret the lithic assemblage characteristics at PP5-6 with respect to mobility will depend on what probable stone raw material sources we identify and how use of those sources changed through time. As will be discussed further below, the coastal characteristics of the area where Pinnacle Point is located might considerably influence the recognition of raw material sources. Some may currently be submerged and others may be hidden under the dunes.

### Pinnacle Point 5–6

PP5-6 is a rockshelter and cave site at Pinnacle Point on the south coast of South Africa near Mossel Bay. The rockshelter and cave formed in the quartzitic cliffs that belong to the Table Mountain Sandstone Group. There are two main sections of the site, PP5-6 North and PP5-6 South, which were probably connected in the past but are now separated by a major eroded area. PP5-6 North is divided into three areas—the Northwest Remnant, the Long Section, and the South Remnant. The Long Section has the temporally longest and vertically thickest main deposit at the site. It is a ~14 vertical meter excavated section of sediment that built up against a cliff face and partially under the rockshelter. That sediment stack overlies an aeolian dune that is at least ~4 m thick, and probably is the same dune that sealed many of the caves on the western side of Pinnacle Point ~90 ka [43]. The rockshelter follows a south-trending fault breccia. An erosion gully that follows the back wall of the shelter has removed some of the original deposit there, and created a west-facing cliff-face that is one of the two cliff-faces in the Long Section targeted for excavation. The other cliff-face is south-facing and resembles a catastrophic detachment of sediment, probably due to high-sea stands [43]. The excavated portion of the PP5-6 North Long Section exposes a continuous section of Middle Stone Age deposits, ~14 m in height, rich in lithic artifacts, fauna, ochre, ostrich eggshell, and hearths.

The PP5-6 North Long Section consists of 11 major geological Stratigraphic Aggregates (StratAggs) defined based on broad-scale changes in sedimentation. These StratAggs are horizontally continuous across large excavation areas and represent a homogenous set of formation processes recognized based on field observations, micromorphology, and GIS-based analysis of structure in the plotted finds [43]. Within the StratAggs there are several other geological stratigraphic groupings at smaller scales that capture more subtle changes in sedimentation. Sub-Aggregates (SubAggs) have a horizontally continuous character beyond a 1 x 1 m square and are typically either palimpsests of combustion features with dense artifact concentrations or strongly geogenic units. Stratigraphic Units (StratUnits) represent small stratigraphic lenses and features and capture the most subtle changes in sediment color and texture. This scale of stratigraphic grouping does not typically extend beyond a 50 x 50 cm quadrant; when it does, each quadrant is further divided into Lots. A Lot is the smallest unit within this system of hierarchical stratigraphic grouping, and excavation occurs at the scale of the Lot. All excavated artifacts within the PP5-6 North Long Section have been piece-plotted using total stations. More than 300,000 plotted finds have been excavated so far.

Below is a summary of each of the geological StratAggs in the PP5-6 North Long Section, from bottom of the sequence to the top. Details about the sedimentology and micromorphology have been reported by Karkanas et al. [43]. More than 65 single-grain OSL age estimates from this section provide a robust and detailed chronology. Optically stimulated luminescence (OSL) analyses were conducted by Zenobia Jacobs. Updated weighted mean OSL ages for each StratAgg were reported by Karkanas et al. [43] and those ages are used here. The Pinnacle Point OSL chronologies have been blind tested with U-Th dating in two separate caves and found to be concordant [42, 136].

*Yellow Brown Sand (YBS)*, *weighted mean OSL age 96 ± 6 ka*, *MIS 5*—aeolian dune at base of sequence, at least 3 m in thickness, minimal anthropogenic input, rare artifacts near contact with overlying StratAgg, corresponds with aeolian event that has also been recognized at other Pinnacle Point sites and is tightly constrained to ~90 ka [42, 136, 137];

*Yellowish Brown Sand and Roofspall (YBSR)*, *weighted mean OSL age 89 ± 5 ka*, *MIS 5—*roofspall rich, contains lenses of combustion features representing single intact hearth structures normally with significant mollusk remains, interbedded with layers of free-fall roofspall with little anthropogenic input, 1.25 m in thickness;

*Light Brown Sand and Roofspall (LBSR)*, *weighted mean OSL age 81 ± 4 ka*, *MIS 5—*sedimentologically similar to YBSR, roofspall rich, contains lenses of combustion features representing single intact hearth structures normally with significant mollusk remains, interbedded with layers of roofspall with little anthropogenic input, ~4.5 m in thickness;

*Ashy Light Brown Sand (ALBS)*, *weighted mean OSL age 72 ± 3 ka*, *MIS 4—*sands with several lenses of dense human occupation and ash rich shell middens, deposited after a significant roof fall event, 0.8 m in thickness;

*Shelly Ashy Dark Brown Sand (SADBS)*, *weighted mean OSL age 71 ± 3 ka*, *MIS 4—*thick deposit of trampled combustion microfacies that represent a cumulative palimpsest of hearth features that are not individually discernable, mollusk remains abundant but not forming a shell supported matrix, the oldest assemblage of backed pieces at Pinnacle Point were recovered from this StratAgg [22], 0.7 m in thickness;

*Orange Brown Sand 1 (OBS1)*, *weighted mean OSL age 69 ± 3 ka*, *MIS 4*—aeolian sand layers with thin layers of trampled and reworked combustion feature palimpsests, mollusk remains present, 0.7 m in thickness;

*Shelly Gray Sand (SGS)*, *weighted mean OSL age 64 ± 3ka*, *MIS 4—*concentrated input of trampled and reworked combustion feature palimpsests, very dense shell-supported matrix in some SubAggs, 0.3 m in thickness;

*Orange Brown Sand 2 (OBS2)*, *weighted mean OSL age 63 ± 3 ka*, *MIS 4*—similar to OBS1, aeolian sand layers with thin layers of trampled and reworked combustion feature palimpsests, mollusk remains appear absent, decalcified, 1 m in thickness;

*Dark Brown Compact Sand (DBCS)*, *weighted mean OSL age 62 ± 3 ka*, *MIS 4*—debris flow that truncates OBS2, SGS, and OBS1, lithic artifacts consistent with strict definition of HP industry [22], earlier phase of the same depositional process that produced the BBCSR (see below), includes eroded sediments of OBS2, 0.75 m in thickness;

*Black Brown Compact Sand and Roofspall (BBCSR)*, *weighted mean OSL age 52 ± 3 ka*, *MIS 3—*previously Black Compact Sand and Roofspall (BCSR), thick black layer rich in burnt finds, 0.5 m in thickness;

*Reddish Brown Sand and Roofspall (RBSR)*, *weighted mean OSL age 51 ± 2 ka*, *MIS 3*—aeolian sand with paleosol formation, centimetric and decimetric roofspall, find density low, overlies entirety of North Long Section, 2.75 m in thickness.

For the analyses presented here, StratAggs were lumped into MIS stages based on their mean OSL age estimate; StratAggs YBSR and LBSR date to MIS 5, ALBS, SADBS, OBS1, SGS, OBS2, and DBCS date to MIS 4, BBCSR and RBSR date to MIS 3. The ALBS represents a major break in the formation of the site and the sedimentation from roofspall-dominated to aeolian [43]. The sedimentation change is roughly concordant with the transition from warmer conditions in MIS 5 to cooler conditions in MIS 4 [138] and also coincides with a shift in coastline distance [44]. For these reasons SADBS and ALBS are included within MIS 4, following Karkanas et al. [43], despite having weighted mean OSL age estimates that situate them right at the MIS 5 to MIS 4 boundary.

### Glacial cycling at PP5-6

The time period represented at PP5-6 covers a portion of MIS 5b and the entirety of MIS 5a, all or at least most of MIS 4, and of the early part of MIS 3. Based on benthic δO^18^ records, MIS 5b and 5a were moderate interglacial periods; MIS 4 is a moderate glacial period with medium-low temperatures; and MIS 3 is a moderate return to near interglacial conditions with medium-high temperatures [33, 138]. At PP5-6, glacial cycling influenced distance to coastline [44, 139], the size of the Paleo-Agulhas plain and the presence of grazing ungulates [49] and C3 adapted animals [47], C3-C4 vegetation types [42], and raw material availability [113, 128], with trickle-down effects on hunter-gatherer economic decision-making and adaptation [67]. Table 1 summarizes the changing conditions at Pinnacle Point by MIS.

**Table 1:** Summary of changing environmental conditions at Pinnacle Point at the scale of MIS.

MIS	Glacial/Interglacial [33, 138])	Mean coastline distance	Rainfall seasonality and vegetation type	Faunal communities	Temporal patterning
**MIS 3**	moderate interglacial conditions	~8 km (3.2 km bootstrapped 95% CI)	winter rainfall increased accompanied by C3 vegetation [42]		
**MIS 4**	moderate glacial conditions	~15 km away (5.3 km bootstrapped 95% CI)	summer rain increased accompanied by an increased frequency of C4 grasses [42]	potential migratory ungulate communities appeared on this exposed coastal plain and seemingly did not migrate north into the interior proper [49]	the first half of MIS 4 indicates vegetation instability, whereas after ~64 ka, C4 grasses stabilized and became common [42]
**MIS 5**	strong (5e) and moderate interglacial conditions (5a-d)	~1.4 km (0.4 km bootstrapped 95% CI) away from current position [44]	winter-rainfall dominated accompanied by C3 dominant shrubby fynbos [42]	C3-adapted animals [47]	towards the end of MIS5 (5a) the frequency of C4 grasses increased [42], while the sea level retreated exposing the low-relief offshore coastal plain [140]

#### Distance to coastline

Glacial conditions in the Pleistocene resulted in lower sea levels across the South Coast of South Africa. Because of the broad, shallow continental shelf of the shores near PP5-6, a small change in sea level there resulted in large changes in the landscape. At some points in the 40 thousand years of Late Pleistocene represented at PP5-6 the coastline was as much as 30 km away [44]. This distance is beyond the normal daily foraging radius of a hunter-gatherer group as documented in modern ethnography. A typical one day roundtrip for hunter-gatherers rarely exceeds 15–30 km [141–143]. Half of this average roundtrip, 8–15 km, represents the daily foraging radius. During the PP5-6 MIS 5 phase of occupation, the coastline was near to site, with a minimum modeled distance of 0.8 km to a maximum modeled distance of 4.1 km [43]. During MIS 4, the coastline was usually far from site, with the mean distances ranging from 10.7 km to 20.7 km at the StratAgg-scale. The minimum modeled distance during MIS 4 is 1.7 km and the maximum distance is 30.0 km [43]. During MIS 3, the coastline was on average 10.1–11.6 km away, with a minimum distance of 7.8 km and a maximum distance of 14.4 km.

**Fig 1:**
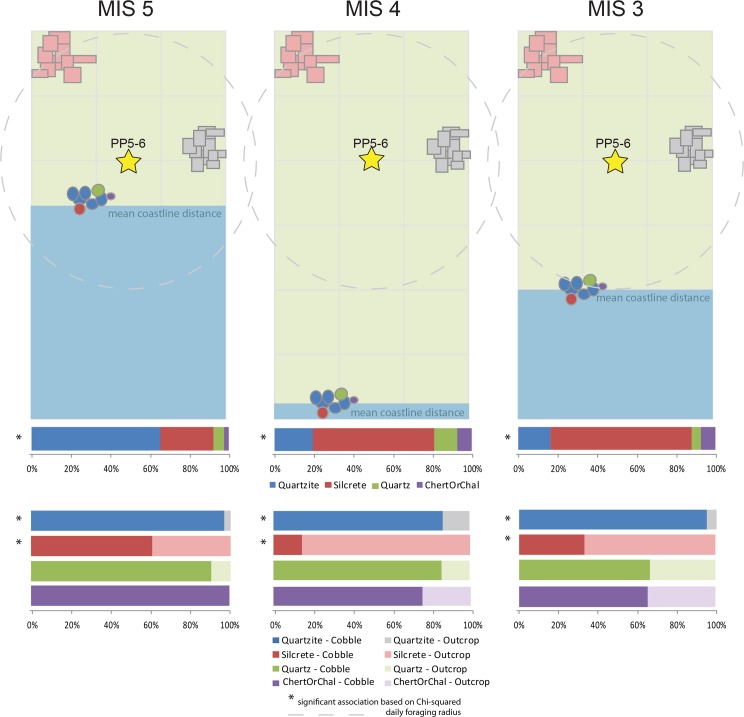
Simplified model of raw material availabilities based on current outcrop locations and mean modelled coastline distance for each MIS [43, 44]. The dispersed distribution of quartz is represented by the light green shade in the background. Each grid square is 5 km x 5 km. The uppermost row of stacked bar graphs present the relative frequencies of each raw material type within each MIS. The lower rows of stacked bar graphs present the relative frequency of cobble and outcrop cortex for each raw material type. Only quartzite and silcrete showed a significant association between cortex type and MIS (Table E in S1 Dataset).

Fig 1 provides a simplified representation of mean distance to coastline at the MIS-scale. To summarize, the coast was always within the daily foraging radius of hunter-gatherers during MIS 5. The coast was generally far, and outside this radius during MIS 4, but distance to coastline fluctuated through this period. The coast was a moderate distance from site in MIS 3, with mean values sitting within the ~8–15 km threshold for hunter-gatherer daily foraging radius. Based on multibeam bathymetry and side-scan sonar, a low-relief ‘plains’ landscape occurred between PP5-6 and the coastline when sea levels were lower than present day [140]. Offshore quartzite outcrops and shelf sand would have provided the substrate for both sandy and rocky shorelines during times of lowered sea-level [140]. Different resources would have been available depending on sea level–coastal and terrestrial resources when the sea was close, and potentially more terrestrial resources when the sea was more than 10 km away. In general, PP5-6 was positioned for the exploitation of both coastal and terrestrial resources in MIS 5, but for the exploitation of more terrestrial resources in MIS 4 and MIS 3. During MIS 3, the coast was within the daily foraging radius. When exposed during MIS 4 and MIS 3, the wide Paleo-Agulhas Plain probably supported large migratory ungulate populations [77, 78] that did not migrate north in to the interior [49]. Dense shellfish remains at PP5-6 in some MIS 4 levels indicate the coastal resources still played a significant role in the adaptive system during those periods, and that these may correlate to short transgressions.

#### C3-C4 vegetation types

Carbon isotope (δ^13^C) records from speleothems at Crevice Cave have been interpreted to indicate changing amounts of C_4_ grass in the immediate area. For most of MIS 5, input from C_4_ grass is interpreted to be minor and consistent with the strongly C_3_ shrubby fynbos vegetation that occurs along the coast today. At the end of MIS 5, there is an increase in the amount of C_4_ grasses at Pinnacle Point, probably in response to increased summer rains [42]. During this period, sea levels were retreating, the low-relief Agulhas Bank was exposed [140], and vegetation in the area around Pinnacle Point was likely grassy fynbos, and possibly sub-tropical thicket [42]. Hunter-gatherers using and discarding lithic material at PP5-6 were more inland, and occupying a different environment with a broader plain between PP5-6 and the ocean. Through roughly the first half of MIS 4, the δ^13^C carbon isotope record indicates instability. After ~64 ka, C_4_ grasses are common again. There is variability within each MIS, but at the coarsest scale, MIS 5 is characterized by a stronger C_3_ signal and MIS 4 by a relatively stronger C_4_ signal. The Crevice record ends at 53 ka, and therefore does not cover the entirety of MIS 3. However, for the first 7 ka the Crevice Cave record suggests a return to more C_3_ conditions with a drop in summer rain.

There is significant correlation between the nearby Crevice Cave δ^13^C carbon isotopes and the types of raw materials discarded at PP5-6 [42, 113]. Peaks in silcrete frequency during the SADBS and DBCS (MIS 4) at PP5-6 correspond to modeled spikes in C_4_ vegetation. It has been suggested that changing raw material frequencies could represent different mobility strategies in response to different environmental conditions [113] and/or different availabilities of wood fuel required for heat treating silcrete [67, 116]. The increased sample sizes reported here permit us to further investigate the pattern reported by Bar-Matthews et al. [42] and Brown [113]. Specifically, this analysis expands on the portions of the MIS 4 PP5-6 sequence between ~69 and 64 ka, and on the earlier part of the MIS 5 occupation ~89 ka.

#### Raw material availability

Glacial cycling at PP5-6 likely had an impact on raw material availability (Table 2, Fig 1). Today, quartzite is the most abundant material near PP5-6 suitable for knapping, but good quality quartzite may not always have been as readily available in the past. The Pinnacle Point caves occur in Skurweberg quartzitic sandstone; however most of this material is coarse-grained and does not fracture conchoidally. The quartzite lithic artifacts at PP5-6 are not manufactured on this material. Water-worn quartzite cobbles are abundant in Eden Bay, directly adjacent to PP5-6, but these derive primarily from the Skurweberg Formation, which is not ideal for lithic reduction (page 81 in [113]). Better quality quartzite is available at this nearby beach, but requires increased time investment to recover [113]. Cobble beaches at Dana Bay, located ~5 km west of Pinnacle Point, are also composed mainly of quartzite, and the material at these beaches are fine-grained and exhibit good potential for knapping. Quartzite cobbles are also available in the De Hoopvlei and Enon conglomerate formations, exposed today, respectively, at 5.5 km and 14 km away from Pinnacle Point, however, there may be other Enon conglomerate exposures closer to PP5-6 (page 215 in [144]). Primary sources of good quality quartzite of the Robberg Formation are available at Cape St. Blaize, 6.4 km from Pinnacle Point.

**Table 2:** Summary of minimum distances to different raw material types in each MIS. Secondary sources (cobbles) depend on modeled sea levels [43, 44]. The distance for primary sources (outcrops) is presented under the assumption that lowered sea levels did not expose good quality outcrops.

	Silcrete		Quartzite		Quartz		Chert/chalcedony	
	Primary	Secondary	Primary	Secondary	Primary	Secondary	Primary	Secondary
**MIS 3**	8.5 km	8–14 km, depending on sea level (mean range 10–11 km)	6 km	8–14 km, depending on sea level (mean range 10–11 km)	<5 km	<5 km	>100 km	8–14 km, depending on sea level (mean range 10–11 km)
**MIS 4**	8.5 km	5–30 km, depending on sea level (mean range 10–20 km)	6 km	5–30 km, depending on sea level (mean range 10–20 km)	<5 km	<5 km	>100 km	5–30 km, depending on sea level (mean range 10–20 km)
**MIS 5**	8.5 km	<5 km	6 km	<5 km	<5 km	<5 km	>100 km	<5 km

Today, and during past interglacial conditions when sea levels were high, the cobble beaches near PP5-6 are continuously refreshed with new cobbles. During past glacial conditions, when sea levels were low, such as during MIS 4, the cobbles in these beaches would have experienced no turnover and may have even been covered by sediment [77, 113, 128]. If the cobble bed was visible, hunter-gatherers exploiting these patches of raw material would have quickly depleted the best quality nodules and there would be no ocean currents replenishing the raw material source. However, the rate of such depletion is not yet known. Rocky shorelines at the new coastline would have been a source for quartzite cobbles during lowered sea levels [140], but these sources would have been up to 30 km away [44].

Silcrete is rarer in the immediate vicinity of Pinnacle Point today. The nearest known primary source of silcrete is 8.5 km northwest of the site at Rietvlei (pages 77, 80 in [113]). However, it remains possible that there is a primary source of silcrete closer to Pinnacle Point currently submerged that may have been exposed when sea levels are low [113]. Silcrete nodules have been encountered in very low frequencies (~1%) at Dana Bay about 5 km from Pinnacle Point (page 81 in [113]). It is possible the silcrete is or was also available as nodules at Eden Bay immediately adjacent to the Pinnacle Point sites due to water currents transporting materials from the Gouritz River Mouth east along the coastline. Further quantitative survey at Eden Bay is required to establish the availability of silcrete nodules there. In any case, silcrete foraging requires a significant increase in time-investment or transport compared to quartzite under current environmental conditions.

Quartz has a broad distribution. It is available in seams within the Skurweberg quartzitic sandstone [144], and is available as small nodules at secondary sources; 4% small quartz nodules were recovered at Eden Bay during a quantitative survey [144]. Surveys have also found that angular quartz nodules are associated with the primary silcrete sources nearest to Pinnacle Point [113].

Other raw material types are rare and only known at the local scale from secondary sources. Hornfels nodules have been recovered in low frequencies (4%) at Dana Bay about 5 km from Pinnacle Point [113]. Cherts and chalcedonies have been recovered from river beds and conglomerates at Hartenbos ~17 km from Pinnacle Point [144]. The Gouritz River (~30 km away) would be another likely source for chert nodules. The Gouritz and its tributaries cut through primary sources of chert in the southern Karoo Basin more than 100 km away [113]. This river also carries chert nodules to the coastline where they can be transported by water currents to cobble beaches near Pinnacle Point [113]. Chert nodules were recovered from Kanon Beach just east of the Gouritz River Mouth about 28 km from Pinnacle Point.

Brown [113] hypothesized that changes in the kinds of raw material used at PP5-6 could be in part explained by distance to coastline. When the coastline was near, similar to the conditions today, there would be greater use of local quartzite and more artifacts with water-worn cobble cortex. In contrast, when the coastline was distant, raw material foraging strategies would shift to primary sources, particularly silcrete, because cobble beaches were no longer rejuvenated with new cobbles. Initial investigation of the PP5-6 assemblage provided some support for this hypothesis [113]; secondary sources of quartzite were more frequently exploited during the LBSR when sea levels are modelled to be high [44], and primary sources of silcrete were more frequently exploited during the SADBS, when sea levels are modelled to be lower. However, the hypothesis is not supported by raw material types in the OBS and SGS when sea level regression peaks. During the OBS and SGS, silcrete frequency is lower than the SADBS, and the frequency of small cobbles of quartz and chert increases [113]. Furthermore, during the DBCS and RBSR, when sea levels where lower than today, there is a high frequency of water-worn cobble cortex. The results presented here, which increase the sample sizes, and occurs at a different scale of analysis (i.e., MIS) permits further consideration of the raw material and coastline distance hypothesis.

## Sample selection and methods

Multiple scales of analysis are possible for the PP5-6 lithic artifacts. Analysis can occur at the level of MIS, technocomplex, geological StratAgg, the more specific Sub-aggregate, or smaller, at the level of the plotted artifact within a Stratigraphic Unit. The maximum amount of behavioral information will come from conducting analyses at each of these multiple scales [118, 123, 145]. This first overall analysis of the assemblage examines variation mainly at the largest scale–MIS.

MIS-scale analysis is appropriate for the first large scale of analysis for the PP5-6 technological sequence for three main reasons. First, there is a precedent for making interpretations about MSA behavioral variability at the scale of glacial periods or MIS as summarized above (e.g. [15, 35, 43, 51, 54, 79–81, 83, 84, 87]) and conducting analyses at the MIS scale permits us to quantitatively test those previously put forth hypotheses. Second, as a coarse-grained climatic framework, MIS is the appropriate framework for testing hypotheses about coarse-grained, long-term, time-averaged behavioral responses to paleoenvironmental change, which is the focus or this investigation. Lastly, this MIS-scale analysis is conducted independently from traditional culture-historic designations (e.g., MSA II, HP, MSA III etc.), and does not make *a priori* assumptions about the structure of the lithic technological variability. Because our question is about the influence of glacial cycling on human technological behaviors, we have chosen not to impose a techno-typological framework at this stage.

The technocomplex concept is problematic for many reasons [146], and is an inappropriate scale of analysis to use here because lithic technological traits can independently vary in response to different factors. For example, while the HP technocomplex is characterized by high frequencies of backed pieces, an emphasis on blade production and the use of fine-grained raw materials, each of these variables can change at different times and in response to different factors. Likewise, one cannot use any trait alone to identify a technocomplex; for example, bifacial points in MSA assemblages are often equated with the SB, but low frequencies of bifacial points come and go through the MSA [118]. When analyses are carried out at the technocomplex scale there is an untested assumption that all the changes in the multiple traits that characterize the technocomplex are coeval and influenced by the same processes. Some recent investigations have found no association between the appearance of new MSA technocomplexes and environment [95, 121–123]. However, those studies did not test for an association between individual lithic assemblage traits and environmental change, and in that way their methodologies differ significantly from ours.

Importantly, it is *not* our goal here to establish what factors best explain all the variability at PP5-6. Nor are we testing whether environment or MIS best explain all the variability at PP5-6, or the appearance of new MSA technocomplexes. The goal is to identifying which, *if any*, individual lithic assemblage traits are associated with MIS. Because lithic assemblage traits are proxies for reduction stage, flaking efficiency, reduction strategy, percussion technique, and tool function they inform us on how higher-level hunter-gatherer behavioral traits associate with MIS, such as demography, provisioning, and mobility strategies. At the MIS-scale, lithic assemblage traits tell us how early modern humans responded on a large, time-averaged scale to major environmental change. Traits that do not associate with MIS have potential to inform of us on other factors that influence technological change, including socio-cultural ones.

The MIS-scale analysis carried out here has two main limitations. First, because it aggregates data, it necessarily leads to the homogenization of data and masks internal variability. Second, the PP5-6 sequence contains only one glacial period, MIS 4 with no other glacial period comparison, so the extent to which the results apply to other glacial periods cannot be assessed using this dataset. For the reasons highlighted above, MIS-scale analysis is still appropriate for addressing our questions about the influence of glacial conditions on Pleistocene technological behaviors. The strength of our approach is that it identifies which lithic assemblage traits demonstrate a cyclical pattern, allowing us to consider human dynamics before, during, and after a glacial period. Analyses at other scales and including sequences from MIS 6 and 2 contexts are forthcoming.

### Lithic analysis

The PP5-6 lithic artifacts were examined by five lithic analysts (JW, KR, BJS, SO, TP) in 2013. The total analyzed sample is more than 14 000 artifacts (Table 3). The team represents different backgrounds and experiences with respect to lithic analysis, and is in line with the goal of optimizing the potential for identifying significant temporal variation and for maximizing comparability with other lithic assemblages in South Africa, East Africa, and Europe. Multi-analyst teams are rare in lithic studies, and we think this approach helps reduce person-specific bias and contributes positively to the reliability of our data set. The methods of analysis were designed to build upon and complement the analyses of Brown [12, 22, 113], who had already examined a sample of ~8000 lithic artifacts from the PP5-6 Long Section. We recorded many of the traits used by Brown (2011) so that the two sets of databases could be combined, and then an additional set of traits that expands the range of possible inter-site comparisons and research questions were documented during the Oct-Nov 2013 season. Our method incorporates traits and typological identifications from multiple sources, including published reports by Villa et al. [106] and Wurz [147, 148] at Klasies River, Wadley [149–151] and Villa et al. [115, 152] at Sibudu, Wadley and Harper [153] and Soriano et al. [108] at Rose Cottage Cave, and Porraz et al. [107] at Diepkloof. A total of 93 different attributes were recorded, which included metric traits such as length, width, and thickness, technologically-relevant traits such as dorsal scar pattern and number of platform scars, and functionally-relevant traits such as presence of “diagnostic impact fractures”. A complete list of recorded traits and definitions is available in S1 File. For analysis some trait categories were lumped together (e.g., ‘small lip’ and ‘large lip’ lumped as ‘lipped’). Many recorded metric traits were converted into technological-relevant ratios (e.g., technological length/technological maximum width), which are indicated in S1 File.

**Table 3:** Summary of analyzed sample from PP5-6.

	Blade or blade fragment	Flake or flake fragment	Shatter	Retouched Piece	Core	Hammer or manuport	Total
**MIS3**	**426**	**1249**	**337**	**75**	**31**	**5**	**2123**
RBSR	103	339	52	33	22	1	550
BCSR	323	910	285	42	9	4	1573
**MIS4**	**1798**	**4882**	**1645**	**417**	**113**	**5**	**8860**
DBCS	416	1218	402	154	24		2214
OBS2	162	686	218	22	14		1102
SGS	103	212	97	17	5		434
OBS1	107	364	118	21	18	1	629
SADBS	961	2173	770	194	47	3	4148
ALBS	49	229	40	9	5	1	333
**MIS5**	**414**	**2209**	**325**	**85**	**60**	**11**	**3104**
LBSR	366	2014	303	81	49	10	2823
YBSR	48	195	22	4	11	1	281
**Total**	**2638**	**8340**	**2307**	**577**	**204**	**21**	**14087**

The total assemblage analyzed amounts to 47% of the plotted lithic assemblage from the PP5-6 Long Section as of 2013 and includes a robust sample from each of the StratAggs and MIS (Table 3). Selection for analysis occurred at the level of the Lot; Lots were selected for analysis in a manner that maximized vertical coverage through the excavation. All plotted finds within an analyzed Lot were examined without a size cut-off, except for cases where the number of artifacts within a Lot was too large and random sampling was required, or when the Lot had been excavated with residue analysis protocol with powder free latex gloves. In the latter cases, a random sample (20%) was preserved for residue analysis.

Analyzed artifacts were washed using an ultrasonic cleaner and soft bristle brush. Those with concreted adhesions were treated with a commercial calcium remover.

Open source software (E4, http://www.oldstoneage.com/software/e4.shtml) was used for data entry. This program, developed by Shannon McPherron and Harold Dibble, is designed to reduce analysis time and to minimize data entry errors. The complete E4 code we developed is available in S1 File.

Raw data, including all specimen numbers, are included in S2 Dataset. All materials are currently housed at the Munro House, Dias Museum, Mossel Bay, South Africa. Once all analyses are complete, the material will be permanently housed at the Iziko South Africa Museum, Cape Town, South Africa. The permit to carry out the work presented here was granted by Heritage Western Cape (1402103TS0225M).

### Statistical analysis

All recorded lithic attributes were included in a statistical analysis comparing the three MIS. The analysis was conducted with no *a priori* predictions of which traits would be influenced by glacial cycling or how. Raw material type was analyzed first, and then frequencies and means of the lithic attributes were calculated for each raw material type (quartzite, silcrete, quartz, and chert/chalcedony) separately. Chert and chalcedony were lumped because of their similar distribution and availability on the landscape near PP5-6, and the difficulties distinguishing between them based on visible characteristics alone [154].

The two different data types in the PP5-6 lithic database–categorical and numerical—were necessarily analyzed differently. Categorical traits such as platform type, dorsal scar direction, and retouched piece typology, were examined via contingency tables and Chi^2^ or Fisher exact test for small samples. Multivariate traits were examined further using correspondence analysis. Numerical traits were examined by using the non-parametric pairwise Wilcoxon test to compare means. All tests were conducted using JMP 11 software.

We propose that a trait that represents a behavioral response to MIS 4 glacial conditions should be more alike before and after MIS 4 than during it. If there is an explanatory association between interglacial-glacial cycles and a given trait, then we expect the following:

1. A significant association between MIS and the trait;
2. MIS 4 different from MIS 5 and MIS 3;
3. The change in values from MIS 5 to 4 and MIS 4 to 3 occurs inversely. For example, if the frequency of a particular trait increases from MIS 5 to 4, then it should decrease from MIS 4 to 3

#### Categorical data

Trait frequencies for each MIS and comparison statistics are provided in Tables D -E in S1 Dataset. A Pearson Chi^2^ test was used to determine whether the distribution of the trait is the same across each MIS. When the contingency table had cells with a count of less than five, a Fisher’s Exact Test was conducted. A trait was determined to have a positive association with MIS when p<0.05. For each of those cases, the temporal trend exhibited by the variability was characterized based on the following criteria and indicated in Table E in S1 Dataset:

1. Glacial cycling—MIS 4 exhibits higher or lower frequencies compared to MIS 3 and 5;
2. Threshold MIS 4—MIS 5 exhibits the highest or lowest frequency, MIS 3 and 4 exhibit similar frequencies;
3. Threshold MIS 3—MIS 3 exhibits the highest or lowest frequency, MIS 4 and 5 exhibit similar frequencies;
4. Temporally-vectored—the trait frequency increases or decreases through time (e.g. MIS 5>MIS 4>MIS 3);
5. Other—there is change through time, but the change does not fit one of the above patterns–usually for multivariate traits where the three MIS plot far from each other on multiple axes (see below).

Correspondence analysis was carried out for multivariate categorical data that exhibited a significant association with MIS (Tables F-H in S1 Dataset, Figs A-D in S2 File). Correspondence analysis is a PCA-type multivariate analysis for count data and is used by archaeologists to compare assemblages in terms of the representation of different artifact types [155, 156]. Correspondence analysis calculates the eigenvalues and eigenvectors of a matrix containing the Chi^2^ distances between all rows. Traits that are strongly associated with MIS will exhibit large absolute eigenvectors, and traits typical for an association with a particular MIS will plot in the vicinity of that association. Traits strongly associated with glacial cycling will exhibit large absolute eigenvectors on the axis that exhibits the most separation of MIS 4 from MIS 5 and 3.

#### Numerical data

Mean values for each MIS and comparison statistics are provided in Tables I-J in S1 Dataset. Means were compared using the nonparametric Wilcoxon Each Pair test. For each case, the temporal trend exhibited by the variability was characterized based on the following criteria and indicated in Table J in S1 Dataset:

1. Glacial cycling—MIS 4 exhibits significantly higher or lower means compared to MIS 3 and 5, MIS 3 and 5 means not significantly different, or the size of the effect is very small;
2. Threshold MIS 4—MIS 5 exhibits the highest or lowest mean and is significantly different from both MIS 3 and MIS 4, MIS 3 and 4 exhibit similar means that are not significantly different;
3. Threshold MIS 3—MIS 3 exhibits the highest or lowest mean and is significantly different from both MIS 4 and MIS 5, MIS 4 and 5 exhibit similar means that are not significantly different;
4. Temporally-vectored—the means are significantly different and they increase or decrease through time (e.g., MIS 5 > MIS 4 > MIS 3).

### MIS-scale values

Two additional values were calculated at the MIS scale–estimated nodule size and blank to core ratio (Tables K-L in S1 Dataset). Nodule size is estimated based on the maximum dimensions of flakes and cores within each MIS, with the minimum representing the maximum dimension minus 10% and the max representing that dimension plus 20% (page 223 in [113]). Blank to core ratio was calculated by dividing the number of complete and proximal flakes and blades by the number of cores.

### Explanation of variability

The above analyses resulted in a summary results table linking each lithic artifact trait for each raw material type with a temporal trend (i.e., glacial cycling, threshold MIS 4, threshold MIS 3, temporally-vectored, other, or no significant association, Tables E and J in S1 Dataset). A Chi^2^ test and correspondence analysis, as described above, were used to test for association and visualize the relationship between raw material and the frequency of traits linked to glacial cycling.

Each trait was also linked to a higher-level behavioral interpretation. For example, cortex amount is often used as an indicator for reduction stage and/or intensity (e.g. [134, 157]). Here, we use the term ‘reduction stage’ to identify which portions of the reduction sequence are represented. Early stages of core reduction such as decortification and preliminary core shaping often produce different kinds of by-products than later stages, such as the ‘optimal’ blade production phase (sensu [108]), core rejuvenation and maintenance. The term ‘reduction intensity’ refers to the use-life of a tool or core–how thoroughly it is knapped or used before it is discarded. The reason reduction intensity and stage have to be lumped for many traits is that if reduction intensity is high, there will be proportionally more products from later stages.

Edge length to mass ratio is used as a proxy for flaking efficiency (e.g. [111, 158]). Here, flaking efficiency refers to the degree to which the cutting edge is maximized for the amount of raw material used [102, 111, 132, 133, 158–162] before retouch (c.f. [163]). It is not referring to the maximization of useable blanks [164] or functional efficiency. We measured efficiency directly using edge length to mass ratio, and indirectly by studying proxies associated with blade production, such as bidirectional scar pattern and frequency of cores with parallel removals. Platform thickness is an example used as a proxy here for percussion technique (e.g. [106, 108, 117]), where percussion technique refers to the manner with which flakes and blades are detached from stone cores, employing direct or indirect percussion or pressure, using hard or soft percussors and marginal or invasive percussion. A list of each behavioral interpretation with references and explanation is provided in Table M in S1 Dataset. By assigning each trait to a single higher-level behavioral interpretation, we are able to offer hypotheses for the temporal variability related to glacial cycling, and explore differences between raw material types using the same statistical tests described above. The behavioral interpretations we assign are generalizations, are not necessarily mutually exclusive, and there are sometimes interpretive contradictions, but as a combined dataset they provide a means for quantitatively exploring possible drivers of lithic variability through time at PP5-6.

### Intra-MIS variability

MIS are not homogenous. Nor are the lithic assemblage traits within each MIS. To address intra-MIS variability, seven key traits that exhibited a significant relationship with MIS and a pattern consistent with glacial cycling were examined at the smallest possible scale. Individual plotted find coordinates were displayed in ArcGIS to observe how variability is distributed across the whole PP5-6 sequence, and to visually evaluate the degree and nature of that variability. Relative point density maps for each of the key traits were generated based on point elevation.

## Results

**Fig 2:**
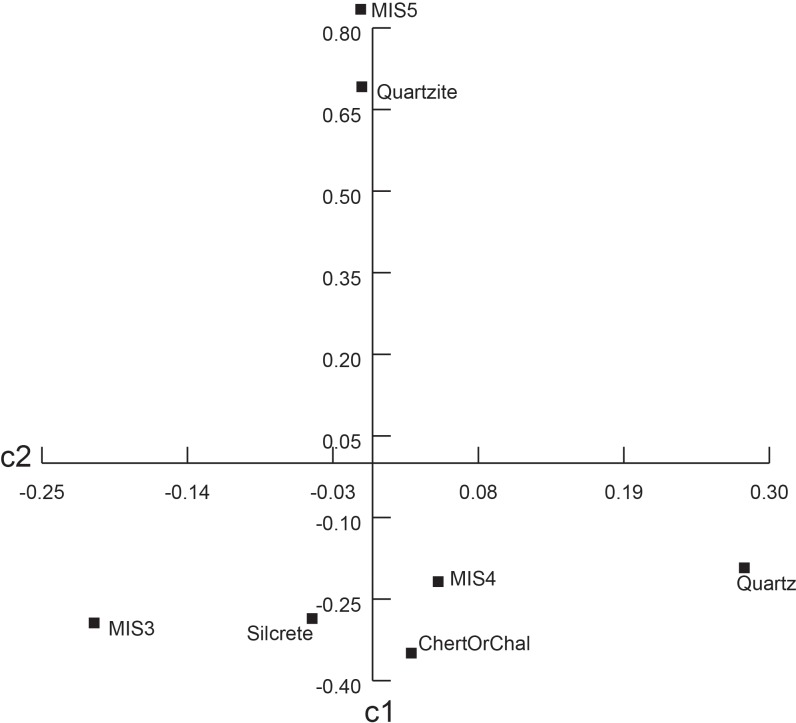
Correspondence plot of raw material types by MIS. c1 explains 96% of variation. c2 explains 4% of variation. Pearson Chi^2^ = 2815, p<0.001.

**Fig 3:**
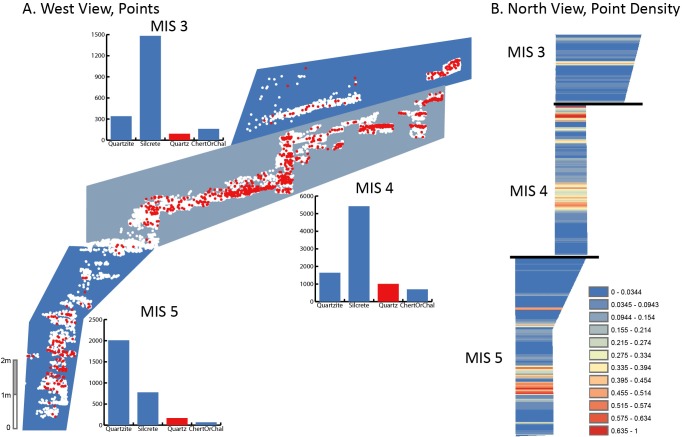
Vertical distribution of quartz frequency at PP5-6. When lumped by MIS stage, relative quartz frequency is highest in MIS 4, though there is variability through the sequence. A. West view of PP5-6 lithic artifact plotted find X,Y,Z coordinates. MIS boundaries indicated by blue bounded areas are defined based on the mean OSL age estimate within StratAggs. Red points are quartz lithic artifacts, white points are lithic artifacts in other raw material types. Bar plots indicate frequency counts of raw material types and MIS. B. Relative point density map of north view of PP5-6 quartz lithic artifact plotted finds generated in ArcMap using the rectangular neighborhood option and based on point elevation. Classes defined based on 1/3 standard deviation. Because some of the deposits dating to late MIS 4 and early MIS 3 overlap with respect to elevation (Z), the MIS 3 relative density map was generated separately from the rest of the sequence and is depicted above the MIS 4 deposits in the point density column.

**Fig 4:**
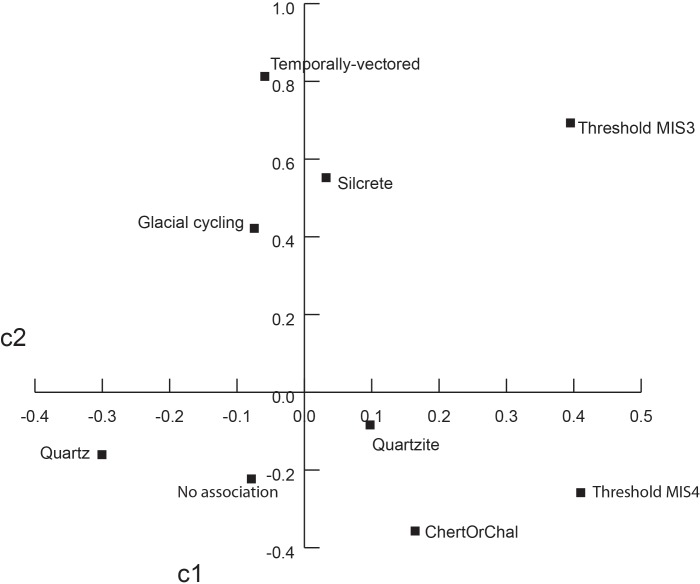
Correspondence plot of raw material types by temporal trend. c1 explains 78% of variation. c2 explains 27% of variation. Pearson Chi^2^ = 51.5, p = <0.0001.

**Fig 5:**
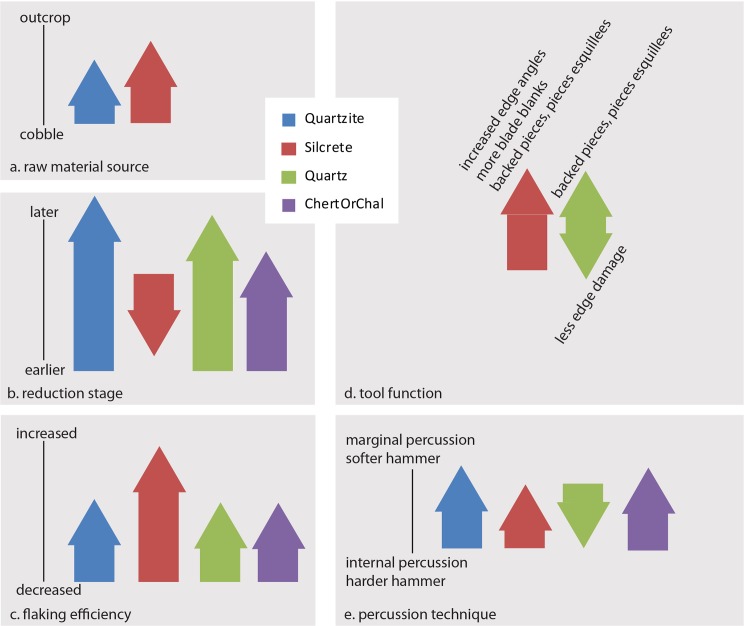
Summary of lithic technological responses to MIS 4 at PP5-6. Each arrow is scaled to the number of proxy traits indicative of that technological response (Table O in S1 Dataset) and the direction of the arrow indicates the direction of the change relative to the y-axis. (a) Proxy traits for raw material source associated with glacial cycling are the presence of cortex chatter marks and interpreted cortex type based on roundness and marks. (b) Proxy traits for reduction stage and/or intensity associated with glacial cycling are cortex area and location, platform cortex, pre-heat treatment area and location, retouched piece typology, visible luster, cortical surface area estimate, dorsal scar count, length of final removal on core face 1, mass, maximum length, maximum thickness, mid-point thickness, pre-heat treatment surface area estimate, and technological length, lithic artifact class (frequency retouched pieces), and blank to core ratio. (c) Proxy traits for flaking efficiency associated with glacial cycling are aris orientation, Conard et al. unified core type, dorsal direction, aris orientation on core face 1, lithic artifact class (frequency blades), edge length to mass ratio, and blank to core ratio. (d) Proxies for tool function or use are presence of edge damage, retouch edge angle, retouched piece blank, retouched piece typology, retouch type, unretouched points, and retouch edge angle. (e) Proxies for percussion technique associated with glacial cycling are fissuring on platform, marks on the ventral surface, platform abrasion, platform delineation, platform morphology, and platform width to thickness ratio.

Descriptive and comparative statistics for all recorded traits are available in Tables A-O in S1 Dataset. Correspondence plots are also presented in Figs A-D in S2 File. Results are summarized here in Figs 1–5, and Tables 3–5. Additional intra-MIS variability plots are available in the Figs E-K in S3 File.

**Table 4:** List of traits by raw material type that associate with MIS and exhibit a pattern consistent with glacial cycling for each raw material type. Traits that do not show a significant association for any raw material type are excluded from this table. Definitions are provided in S1 File.

	Quartzite	Silcrete	Quartz	Chert and chalcedony
ArisOrientation		x		
BlankToCoreRatio	x			
Bulb			x	
CoreConardUnifiedType		x		
CoreVolmanTypeOne			x	x
CoreSurfaceAreaEstimate		x		
CortexArea		x		
CortexChatterMarks		x		
CortexLocation		x		
CortexSurfaceAreaEstimateFlake		x		
DorsalDirection	x	x		x
DorsalScarCount			x	
EdgeDamage			x	
Edge Length EL	x	x		
Edge Length to Mass EL/M	x	x		
Face1DorsalDirection		x		
Face1LengthOfFinalRemoval		x		
FissuringOnPlatform		x		
Flake Platform Area Estimate	x			
FlakeSurfaceAreaEstimate	x		x	
FlakeTermination		x		
FractureInitiationPoint			x	
InferredCobbleType	x	x		
LithicArtifactClass	x		x	
MarksVentralSurface		x	x	x
Mass	x	x	x	x
MaxLength	x		x	
MaxTechWidth	x	x	x	
MaxThickness	x		x	x
MaxWidth	x	x	x	x
MidThickness	x		x	
PlatAbrasion		x		
PlatformCortex		x		
PlatformDelineation		x		
Platform W/T	x			x
PlatformMorphology	x	x		
PlatformThickness	x	x	x	
TechLength	x			
PostDepBurning		x		x
PreHeatArea		x		
PreHeatSurfaceAreaEstimateFlake		x		
PreHeatTreatmentLocation		x		
ProfileShape		x		
RetouchEdgeAngle		x		
RetouchedPieceBlank		x		
RetouchedPieceTypology		x	x	
RetouchType		x	x	
TechnicalCategory		x		
TechLength			x	x
VisibleLuster		x		

**Table 5:** Summary of results.

Inference Category	MIS 3 (57–29 ka)	MIS 4 (74/71-57 ka)	MIS 5 (130-74/71 ka)
**StratAggs represented at PP5-6**	RBSR, BBCSR	ALBS, SADBS, OBS1, SGS, OBS2, DBCS	YBSR, LBSR
**Raw material type**	Silcrete dominant, relatively high frequency of chert, low frequencies of quartzite and quartz	Silcrete dominant, relatively high frequency of chert and quartz, low frequency of quartzite	Quartzite dominant, moderate frequency of silcrete, low frequencies of quartz and chert
**Raw material source**	Evidence for both cobble and outcrop sources	Higher frequency of quartzite and silcrete artifacts with evidence for outcrop sources	Mainly cobble sources, few artifacts with evidence for outcrop sources
**Reduction stage and/or intensity**	All reduction stages present based on presence of cortical pieces, small knapping debris, and retouched pieces	All reduction stages present, relatively later stages or more intense reduction of quartzite, quartz, and chert, earlier stages or less intense reduction of silcrete	All reduction stages present based on presence of cortical pieces, small knapping debris, and retouched pieces
**Flaking strategy and efficiency**	Diverse blade and prepared core reduction strategies. Discarded core types include parallel and platform cores (Conard et al. 2004), pyramidal blade cores, recurrent centripetal cores, and preferential point cores (Table D in S1 Dataset).	Diverse blade and prepared core reduction strategies, evidence for increased flaking efficiency for all raw material types, most pronounced change in flaking efficiency observed for silcrete based on increased EL/M, and increased frequency of blades, blade core types, and parallel cores.	Diverse blade and prepared core reduction strategies. Discarded core types include parallel and platform cores [168], pyramidal blade cores, recurrent centripetal cores, and preferential point cores (Table D in S1 Dataset).
**Tool types and function**	Diverse tool types including backed pieces, points, notched pieces, *pieces esquillées*	Diverse tool types including backed pieces, points, notched pieces, *pieces esquillées*, Silcrete—more backed pieces and *pieces esquillées*, more blade blanks, increased edge angles (backing), Quartz—less edge damage, more backed pieces and *pieces esquillées*	Diverse tool types including points, notches, *pieces esquillées*, no backed pieces
**Percussion technique**		Potentially more evidence for marginal percussion with softer hammer for quartzite, silcrete, and chert	

### Raw material type

There is a significant association between raw material type and MIS (Pearson Chi^2^ = 2815, p<0.001). Silcrete and chert/chalcedony frequencies are high in MIS 4 and MIS 3, whereas quartzite frequency is high in MIS 5. Based on correspondence analysis, the main trend of variation for raw material type on axis 1, which explains 96% of the total variation (Table C in S1 Dataset), runs from high quartzite frequencies associated with MIS 5 and high silcrete and chert/chalcedony frequencies associated with MIS 3 and 4 (Fig 2). The quartz dimension makes a small contribution to the difference between the glacial (MIS 4) and interglacial (MIS 3 and 5) periods on axis 2. Only quartz shows similar frequencies in MIS 3 (4.4%, n = 91) and 5 (5.6%, n = 168) that differ from the frequency in MIS 4 (11.5%, n = 1008). In other words, only quartz frequency exhibits a pattern consistent with a glacial cycling effect. During the glacial period, there was overall an increased frequency of quartz use compared to the interglacial periods, and other raw material types do not fit this pattern.

A consideration of the vertical variability through the PP5-6 sequence demonstrates that quartz frequency fluctuated through time. There are periods during MIS 4 when quartz frequency was low, and periods during MIS 5 and 3 when quartz frequency was high; however, during MIS 4 there are longer stretches of the sequence with 16% or more quartz and several pulses with 40% or more (Fig 3).

### Lithic attributes

Table 4 lists all attributes by raw material type that exhibit temporal change consistent with an effect from glacial cycling. The traits that exhibit other types of temporal change can be found in the S1 Dataset, and are not discussed further here. For quartzite, 22% (n = 18) of traits show variability consistent with glacial cycling, 39% (n = 35) for silcrete, 22% (n = 18) for quartz, and 11% (n = 9) for chert/chalcedony. Silcrete exhibits the highest frequency of traits that show a pattern consistent with an effect from glacial cycling. There is a significant association between the frequency of different temporal trends and raw material (Pearson Chi^2^ = 51.5, p = <0.0001). On a correspondence plot (Fig 4, Table N in S1 Dataset), silcrete, glacial cycling, temporally-vectored, and threshold MIS 3 associate on one end of axis 1, which explains 78% of the variability, while quartzite, chert, quartz, no association, and threshold MIS 4 associate on the other end of axis 1. At PP5-6, silcrete was treated differently during the glacial occupation (MIS 4) than it was during the interglacial occupations (MIS 3 and 5). All raw material types were affected by glacial cycling to some extent, however, silcrete exhibits significantly more traits influenced by glacial cycling.

The lithic artifact traits are proxies for a number of behaviors: changing raw material sources, reduction stage and/or intensity, flaking efficiency, tool function, and percussion technique (Table M in S1 Dataset). With a couple of exceptions discussed further below, most behavioral interpretations are represented at least once within each raw material type category (Fig 5, Table O in S1 Dataset). And, for the traits that show a pattern consistent with an effect from glacial cycling, there is no significant association between raw material type and behavioral interpretation (Fisher’s Exact Test, p = 1.000, each of four tests). In other words, hunter-gatherers occupying PP5-6 altered the way they treated quartzite in multiple ways, which included shifts in changing raw material sources, reduction stage, flaking efficiency, tool function, and percussion technique. The same is true for every other raw material type. However, in some cases the nature and direction of these changing treatments differs between the raw material types (Fig 5), as discussed further below.

#### Changing raw material sources in response to glacial cycling

Raw material sources differed in MIS 4 compared to MIS 3 and 5 for quartzite and silcrete. Both quartzite and silcrete artifacts show a significant increase in the frequency of outcrop cortex in MIS 4 (Fig 1). A consideration of the vertical variability through the PP5-6 sequence demonstrates that the relative frequency of outcrop cortex fluctuated through time. There are periods during MIS 4 when quartzite and silcrete outcrop cortex frequency was low, and periods during MIS 5 and 3 when the frequencies were high. However, for quartzite, there are longer stretches and more pulses with a frequency of outcrop cortex of 12% or more in MIS 4, especially in the lower part of the MIS 4 sequence. In MIS 3 and 5, there appears to have been fewer and shorter pulses with more than 12% (Fig E in S3 File). For silcrete, there are long stretches of the sequence with 56% or more outcrop cortex and many pulses with 94% or more during MIS 4 (Fig F in S3 File). Quartz and chert show no significant change in inferred cortex type. Estimated nodule size does not exhibit change related to glacial cycling for any raw material type (Table K in S1 Dataset).

#### Changing reduction stages and/or intensity in response to glacial cycling

Quartzite artifacts at PP5-6 are overall smaller in size based on mass, maximum length, technological length, and maximum width and thickness measurements, and exhibit a higher blank to core ratio, perhaps consistent with later stages of reduction and/or increased reduction intensity [165]. Fig G in S3 File shows the vertical distribution of quartzite technological length through the PP5-6 sequence; the frequency of complete pieces greater than 44 mm in length is less than 21% throughout MIS 4. In contrast, there are several pulses during MIS 3 and 5 that demonstrate high frequencies (≥ 37%) of complete pieces greater than 44 mm in length. Silcrete, on the other hand, shows more traits consistent with earlier stages of reduction or less intensive reduction in MIS 4. The frequency of flakes with cortex and pre-heat treatment surfaces is highest in MIS 4. There is intra-MIS variability; however, there are longer stretches with a frequency of 12% or more pieces with partial or complete cortex in MIS 4 compared to MIS 3 and 5 (Fig H in S3 File). The frequency of silcrete pieces that lack visible luster (i.e., they were potentially detached from a core prior to heat treatment) is highest in MIS 4. Cortex is less frequently located distally on silcrete artifacts in MIS 4, also consistent with earlier stages of reduction or less intensive reduction [157]. The frequency of silcrete pieces with platform cortex is highest in MIS 4. Fewer silcrete traits are indicative of later stages of reduction, including measures of size (mass and width measurements). Also, the length of the final removal is smallest in MIS 4, consistent with higher degrees of core exhaustion [166]; there is no significant difference in estimated original nodule size between MIS, so original nodule size cannot explain this pattern. Many quartz artifact metric dimensions–mass, maximum length, maximum technological width, maximum thickness, maximum width, mid-thickness, and technological length are smaller in MIS 4 then MIS 3 and 5, perhaps consistent with later stages of reduction or more intense reduction [166]. Dorsal scar count shows the opposite trend, decreasing in MIS 4. Chert artifact mass, maximum thickness, maximum width, and technological length are lowest in MIS 4, consistent with later stages of reduction or more intense reduction. In sum, quartzite, quartz, and chert show some evidence for later stages of reduction or more intense reduction during MIS 4, whereas silcrete shows more traits consistent with earlier stages of reduction.

#### Changing flaking efficiency in response to glacial cycling

All raw material types show evidence for increased flaking efficiency, that is, the degree to which the cutting edge is maximized for the amount of raw material used, during MIS 4. Quartzite exhibits glacial cycling related changes in four traits that are proxies for flaking efficiency–dorsal direction, blade frequency, edge length to mass ratio, and blank to core ratio. There is a higher frequency of pieces with bi- or unidirectional scars during MIS 4 than during MIS 3 and 5. Bi- and unidirectional elongate and parallel scars are associated with blade production, which is often linked to more efficient reduction strategies [102, 133, 159], but see discussion below. Quartzite blade and blade fragment frequency is also highest in MIS 4. During MIS 4, quartzite exhibits a mean edge length to mass ratio that is highest in MIS 4. Fig I in S3 File shows the intra-MIS variability exhibited by this trait. In MIS 4, there are longer stretches where the frequency of pieces with edge length to mass ratio of 51 or more exceeds 19%. Pulses of 19% or more are rare in MIS 5 and 3. The blank to core ratio is highest in MIS 4 for quartzite. Silcrete also exhibits traits consistent with increased flaking efficiency in MIS 4 –bi- or unidirectional dorsal direction on flakes and cores, parallel dorsal scars, and increased edge length to mass ratio (Fig J in S3 File). Silcrete shows an association between MIS 4 and blades/blade fragments and parallel core types (Fig A in S2 File). Quartz shows an increased frequency of blades and cores for the production of detached pieces with parallel or convergent scars (Fig C in S2 File). Chert shows an increase in flakes with bi- and unidirectional dorsal scars, and cores for the production of detached pieces with parallel or convergent scars and cylinder cores (Fig D in S2 File).

#### Changing tool function in response to glacial cycling

Quartzite shows no change in most traits lumped here as related to tool function, including retouched piece typology, retouched edge angle, and retouched piece blank, unretouched point frequency, or edge damage frequency. Quartzite does show an increase in the relative frequency of retouched pieces in MIS 4, and MIS 4 associates with retouched pieces in a correspondence plot of MIS versus lithic artifact class (Pearson Chi^2^ = 299.9, p <0.001, Fig B in S2 File), but this could also be a reflection of increased reduction intensity. Silcrete, on the other hand, shows changes in multiple traits related to tool function–such as increased retouched edge angle, increased relative frequency of blades as blanks, a high frequency of backed pieces and *pieces esquillées*, and a high frequency of retouched pieces in MIS 4. Quartz shows lower frequencies of pieces with edge damage/utilization and increase in the frequency of backed pieces and *pieces esquillées*.

#### Changing percussion technique in response to glacial cycling

The analysis conducted here included a specialized database of platform and ventral surface characteristics advocated for by Soriano et al. [108] and Villa et al. [106], based on a report by Pelegrin [167], and utilized by other MSA researchers [117] for determining percussion technique (i.e., percussor type, pre-impact preparation, impact location relative to core edge). For all the raw material types, there are some significant changes in these platform characteristics associated with glacial cycling. Quartzite exhibits significantly thinner platforms based on platform width to thickness ratio, and platform morphology (Fig B in S2 File), perhaps consistent with more marginal percussion. Silcrete exhibits a change in platform morphology (MIS 4 associated with narrow linear platform, Fig A in S2 File), increased shattered bulbs and contoured Hertzian cones on the ventral surface, increased occurrence of double platform delineation, few contoured fissures, and more platform abrasion. In general, the combination of traits exhibited by silcrete artifacts at PP5-6 are consistent with direct marginal percussion with a soft stone hammer as observed for HP assemblages at Rose Cottage Cave and Klasies River [106, 108]. The increased shattered bulbs, contoured Hertzian cones, and double platform delineation in MIS 4 could reflect earlier stages of reduction for silcrete with a harder stone hammer. Quartz shows an association with shattered bulbs in MIS 4. In MIS 4, chert shows an association with thinner platforms, consistent with more marginal percussion, but also more marks associated with a stone hammer. Based on the variability exhibited by the platform and ventral characteristics, there is a significant association between percussion technique and MIS, with MIS 4, in general, showing increased evidence for marginal soft stone hammer percussion.

## Discussion

This study analyzed the lithic technological record at PP5-6 between ~90 and 50 ka with a focus on change over time at the scale of MIS. Here we focused on identifying traits that exhibit a pattern consistent with an effect from glacial cycling; they are more alike before and after MIS 4 than during it. A summary of the results is presented in Table 5. A large number of the traits that vary with glacial-interglacial cycles involve how foragers were accessing and processing silcrete and how they were knapping it. Compared to the interglacial occupations at PP5-6, MIS 4 occupants were making increased use of outcrop sources of silcrete. Based on the higher frequency of silcrete pieces that lack visible luster and exhibit cortex, they were preparing nodules by removing detachments prior to heat treatment onsite at levels higher than during MIS 5 and 3. The silcrete core reduction methods utilized during MIS 4 emphasized blade production and resulted in a significantly higher cutting edge to mass ratio. These methods produced an assemblage with increased frequency of flakes and blades with uni- or bidirectional scars relative to flakes with radial or subradial scars. Discarded cores more commonly exhibit removals parallel to a plane of intersection between two faces, and were reduced to a smaller size and with smaller final removals. Many of the core reduction method traits just described relate to maximizing the amount of useable cutting edge (i.e., flaking efficiency, see further discussion below). Preferential selection for silcrete blades as blanks for retouched pieces was more pronounced during MIS 4 than MIS 5 and 3. Retouched piece frequency on silcrete is associated with the interglacial-glacial cycle at PP5-6, with higher frequencies associated with MIS 4. Glacial conditions at PP5-6 triggered changes in the nearby environment, and early human foragers using PP5-6 increased their focus on outcrop silcrete, increased the frequency with which they discarded earlier stage silcrete products at the site, and employed more efficient blade-focused reduction methods for that silcrete.

To carry out the analyses here, we assigned individual lithic traits to higher-level behavioral interpretations, such as flaking efficiency and reduction intensity. When contradictory studies have been published for a given interpretation, we acknowledge it in Table M in S1 Dataset. For example, one argument that we have presented here is that increased evidence during MIS 4 for silcrete blade production reflects increased flaking efficiency. Blade production has been traditionally linked to flaking efficiency and raw material conservation because blades provide relatively more cutting edge than flakes [102, 133, 159]. However, under at least some experimental conditions and when different measures of efficiency are used, blade production can be less efficient than flake production [163, 164]. Eren et al., 2008 show that, while blades do have more cutting edge and higher cutting edge/mass ratio than flakes, this apparent gain in efficiency disappears when you consider the whole process of blade-making (i.e., the removal of preparation flakes), and retouch (because the broad shape of flakes mean they can tolerate more cycles of retouch). Jennings et al. [164] show that blade cores do not produce more useable blanks then bifacial flake cores. Therefore, one may argue that we should discount evidence for increased blade production as a proxy for flaking efficiency. If we do that, our database still shows that edge length to mass ratio increases in MIS 4. Even excluding evidence for increased blade production as a proxy for increased flaking efficiency our behavioral interpretation does not change. As noted in the methods section, the behavioral interpretations we assign are generalizations, and there are sometimes interpretive contradictions, but as a combined dataset they provide a means for testing hypotheses. Interestingly, there is a positive correlation between blade production, edge-length to mass ratio, and blank to core ratio at PP5-6, as revealed by this study. This relationship can now be experimentally investigated on local raw material types and using core reduction strategies represented in the PP5-6 sequence.

Why increased use of outcrop silcrete in MIS 4? When sea levels retreated, the nearest secondary sources of silcrete were probably located further away than primary sources (Fig 1). In MIS 5, sea levels were near and silcrete pebbles could be foraged from nearby cobble beaches. Outcrop sources were used and their products discarded at PP5-6, but to a lesser degree than during MIS 4. In MIS 4, the coast was on average about 20 km away so cobble beaches with replenishing sources of silcrete pebbles were located beyond the daily foraging radius of hunter-gatherers. Silcrete outcrops were within the daily foraging radius, however. Today, the nearest source is located ~8km to the NW of PP5-6. This may have been true through much of the Pleistocene unless a nearer primary silcrete source was exposed on the Paleo-Agulhas Plain when sea levels retreated. Further investigations that address this pattern are required. During MIS 3, the coast and thus, replenished cobble beaches with silcrete pebbles, were much closer than during MIS 4 and the assemblage contains higher frequencies of cobble cortex.

Why are earlier stages of reduction for silcrete better represented during MIS 4? This is a surprising result that defies an easy explanation. This pattern could be explained by the exposure of a primary source of silcrete during sea level retreat that was located nearer to site than the known source today, which is 8 km away. When raw material sources are nearer, we expect some evidence of earlier stages of reduction (e.g. [169]). However, that same source would have been exposed during MIS 3 when the coastline was ~11 km away (Fig 1), while others presently known could have been cover by dunes. A nearby source of primary silcrete does not seem to have been readily available in MIS 3, because during MIS 3 there is increased focus on silcrete cobbles and there are fewer products from earlier stages of reduction compared to MIS 4. Another potential explanation for earlier stages of reduction for silcrete in MIS 4 could be the provisioning strategy–place versus people provisioning [170, 171]. In this scenario, MIS 4 would represent a strategy on the ‘place’ side of the provisioning strategy continuum, where silcrete was transported across the landscape in order to ensure that prime locations for occupation and resource extraction were stocked with the needed stone resources. This result is consistent with Mackay et al.’s [51] argument for evidence of ‘place provisioning’ in the HP. In contrast, MIS 3 and 5 may represent strategies closer to the ‘people’ side of the provisioning strategy, where silcrete was still transported across the landscape, but to a lesser extent, in smaller quantities, and on a needs-basis with less long-term planning. This explanation is consistent with the increased proxies for earlier stages of reduction represented in the MIS 4 occupation at PP5-6 that are rarer in the MIS 3 and 5 occupations.

Why use more efficient methods of reduction for silcrete in MIS 4? Our results show an increase in edge length to mass ratio, and an increase in blade production as measured by bidirectional dorsal directions and presence of blade cores, during MIS 4. Presumably these efficient methods are employed to maximize cutting edge while reducing costs associated with transport or search time for new nodules. The need to maximize efficiency may be linked to an increased distance from raw material sources, because efficient methods can balance out increased transport cost. The need to maximize efficiency may also be linked to changes in mobility [131, 133], and/or increased group or population sizes, because efficient methods can maximize the use life of raw materials that are at risk of depletion due to longer stays or more people [135]. At PP5-6, it is possible that any or all of these three explanations may be correct, but the latter two are more likely. The first explanation can be ruled out because all raw material types, not just silcrete, show an increase in flaking efficiency during MIS 4. Increased flaking efficiency is probably not a response to raw material availability because the same response occurs during MIS 4 across all raw material types, which exhibit different availability shifts in MIS 4. Furthermore, during MIS 3, primary source availability is presumably the same based on current evidence as for MIS 4, and beach cobbles are still ~11 km away. In other words, the transport distance of silcrete is as high during MIS 3 as MIS 4, but silcrete is not knapped using the same efficient method.

The latter two explanations–changes in mobility or increased group/population size–are difficult to tease apart, and both are potentially reflected in the sedimentology of the site through MIS 4. Compared to MIS 5, the MIS 4 deposits at PP5-6 exhibit thick palimpsests of anthropogenic burnt remains, consistent with increased occupation duration and/or intensity [43].

If the technological changes in MIS 4 are a reflection of changing mobility strategies, rather than, or in addition to increases in group and/or population size, then in what way did mobility change? This question is difficult to answer and we propose the following causal mechanism. When sea levels are lower during MIS 4 and MIS 3, local raw material availability is overall low, and good sources of silcrete are at least 8 km from site (Table 2, Fig 1). Knappers at PP5-6 may have employed methods that increased flaking efficiency during MIS 4 because they needed to conserve a limited silcrete supply. Their silcrete supply may have been relatively limited compared to earlier and later periods because they had lowered levels of residential mobility; when local raw material availability is low and/or depleted, less mobile hunter-gatherers react by increasing flaking efficiency [131, 133]. Changes in resource availability and distribution that resulted from lowered sea-levels and the exposure of the Agulhas Bank during MIS 4 may have driven the hunter-gatherer populations utilizing PP5-6 to have reduced the number of residential moves they made. During MIS 4, silcrete is knapped using more efficient reduction strategies that result in an assemblage with increased evidence for blade production, higher cutting edge to mass ratio, and higher blank to core ratio. The PP5-6 lithic technological record is consistent with reduced residential mobility during glacial MIS 4 compared to interglacial MIS 3 and MIS 5. Effective local raw material availability was potentially lower in MIS 4 than MIS 3, because groups were occupying the site for longer duration and continuously exploiting the nearest sources. This interpretation of changing mobility patterns at PP5-6, with a shift toward decreased residential mobility and increased sedentariness, is inconsistent with the argument that mobility increased during MIS 4 [35].

An important result of this analysis is that silcrete frequency at PP5-6 does not associate with the MIS 5-4-3 interglacial-glacial cycle. Silcrete frequency significantly increases in MIS 4 and then this high frequency persists into MIS 3. There is a change in *how* silcrete is exploited at PP5-6 that is related to glacial cycling, but *not how frequently* silcrete debris was discarded at site. It is possible that this pattern of silcrete persistence through MIS 3 is explained by modeled sea levels during this time–the coast and rejuvenated quartzite cobble beaches are ~10–11 km away and further away than primary sources of silcrete (~8 km). But good quality primary sources of quartzite were always available ~6 km from site through MIS 4 and MIS 3, so if distance were the only factor explaining raw material choice, silcrete would not dominate either MIS 4 or MIS 3.

Quartz frequency, on the other hand, is associated with interglacial-cycles, and is significantly higher during MIS 4. Quartz is available in several different forms and from several different locations on the landscape. It does not have a restricted distribution. During MIS 4, PP5-6 occupants were either targeting quartz to a higher degree because of functional or stylistic requirements, their mobility system caused them to intercept more or better quartz sources, or local sources of quartz were exploited more intensely because of changes in site occupation intensity. The latter is consistent with the other observations described above. Quartz frequency increases in the MIS 4 occupations at other sites in South Africa, including Klasies River [106, 147, 148], and in the mid-MIS 4 levels at Klipdrift shelter [172], and Klein Kliphuis [173]. At Diepkloof, elevated quartz frequencies are associated with some HP levels [107]. The same pattern is not apparent at Blombos Cave [19], Rose Cottage Cave [108], or Sibudu Cave [115]. Changes in raw material source exploitation in response to glacial cycling are probably best understood within a local or regional context.

There are several silcrete traits that show no association with the MIS 5-4-3 interglacial-glacial cycle. One of these traits is estimated nodule size. Even though MIS 4 occupants were more often exploiting primary sources of silcrete than MIS 5 and 3 occupants, raw material package size does not appear to differ. Foragers were transporting small (~80 mm) silcrete nodules to PP5-6 throughout the sequence. When sea levels were higher, and active cobble beaches were closer, they transported mainly small beach cobbles with water-rolled cortex. When sea levels were lower, and active cobble beaches were farther, they transported mainly small nodules from primary sources. The implication here is that raw material package size is not a good explanatory factor for the patterning reported here.

For quartzite, quartz, and chert, fewer traits exhibit an association with interglacial-glacial cycles. However, many exceptions to this pattern relate to raw material flaking efficiency. All raw material types show some evidence for increased flaking efficiency. Core reduction strategies seem to have been modified to maximize the efficiency of quartzite, quartz, and chert, though not to the same degree or in the same way as for silcrete. The same potential explanations for silcrete apply to the other raw materials; the need to maximize efficiency may be linked to (1) an increased distance from those raw material sources, (2) changes in mobility, and/or (3) increased group or population sizes. For quartz, only the latter two explanations are consistent with all the observations discussed above, because quartz availability did not change with shifting coastline distances (Fig 1). The evidence for increased flaking efficiency observations at PP5-6 is consistent with the pattern for increased blade production [51], and increased edge length to mass ratio [111] in the MIS 4 occupations at other sites and on different raw material types.

Backed piece presence is one of many assemblage traits that show major change with the shift from MIS 5 to MIS 4, and little change with the shift from MIS 4 to MIS 3. Once backed pieces appear in the archaeological record at PP5-6 they persist through the sequence. They are present in the SADBS dated by OSL to 71±3 ka, in the RBSR dated by OSL to 51±2 ka, and in all the StratAggs in between (OBS1, SGS, OBS2, DBCS, BBCSR). There is a decrease in frequency in the MIS 3 levels (12.2%, n = 9/67 retouched pieces) compared to the MIS 4 levels (18.8%, n = 65/345 retouched pieces) but the difference is not significant based on this sample (Fisher’s exact test, p = 0.3843). The presence of backed pieces in the MIS 3 occupations at PP5-6 is consistent with observations by others at Diepkloof Rockshelter [107], Rose Cottage Cave [108], and Sibudu Cave [109], and goes against the dominant perspective that backed piece technology flickered in and out of fashion for a brief period of time during MIS 4 (e.g. [89, 93, 98, 101, 102]).

Another trait that persists through MIS 3 that was already mentioned is increased silcrete frequency. Silcrete frequency significantly increases through time at the MIS scale, but the size of the effect is much greater when comparing MIS 5 and MIS 4. Once silcrete becomes a dominant component of the assemblage at PP5-6, it persists. As discussed above and based on current evidence, distance to source alone does not explain this pattern. This result is consistent with observations at Diepkloof Rockshelter [107], Rose Cottage Cave [108], and Sibudu Cave [115], where changing raw material frequencies are more muted than has been generally emphasized in the HP literature. The shift in silcrete use in MIS 4, together with the other technological changes that persist through MIS 3, may represent a sort of technological ‘tipping point’ for Late Pleistocene foragers in this region that persist once they are established. Once foragers preferred silcrete to the point that silcrete represents a large proportion of the assemblage, and backed pieces were manufactured mainly on that silcrete, those traits persevere despite the interglacial-glacial cycles and the changes in raw material availability and knapping efficiency described above. Emphases on silcrete-use and backed pieces have a long duration at PP5-6, but an increased emphasis on blade production is associated with MIS 4. Thus, the techno-typological traits that characterize the HP vary independently on a temporal scale at PP5-6, and this observation challenges the usefulness of applying a rigid and restrictive techno-typological framework to the sequence at this time.

Two major artifact categories decrease in frequency though time at the MIS scale. Notched pieces significantly decline in frequency between MIS 5 and MIS 4 and the frequency remains low in MIS 3. Unretouched point frequency is highest in MIS 5 and lowest in MIS 3. Interglacial-glacial cycles do not explain changes in unretouched point and notched piece frequencies at PP5-6. These artifact categories are significant because they are often used to typologically distinguish different phases in the MSA (e.g. [107, 147]). These results do not support the idea that at the largest scale of MIS stages, there is an association between environmental change and these typological characteristics. Future analyses at smaller scales may show temporal changes more consistent with other published reports with respect to notched pieces and unretouched points.

Many traits do not associate with MIS and/or do not show a temporal pattern consistent with an effect from glacial cycling. Some of these are mentioned above, such as an emphasis on the use of silcrete, and the frequency of particular retouched tool types (notched pieces, unretouched points). The traits not listed in Table 4 are not linked to environmental change at the scale of MIS and may be better explained by socio-cultural factors. For example, a preference for silcrete could represent a cultural tradition that was maintained for millennia through social learning [174]. The decision to choose silcrete over quartzite might not be explained solely by economic factors, which is a perspective in line with a recent study of MSA and LSA silcrete abundance [175], and hypotheses that link raw material selection to aspects of reciprocal exchange and symbolism [52, 53]. The traits not listed in Table 4 are also good candidates for understanding the meaning of continuity in material culture style across space in South Africa and for exploring the ideas that similarities were reinforced by contact and exchange among and between early modern human foragers [51, 92, 172, 176], that short-term temporal changes in technological behavior and differences between sites could reflect local socio-cultural historical trajectories [56, 118, 177], and that a holistic consideration of what drove MSA technological change will include a consideration of non-environmental factors [95, 119, 121, 123]. Further investigations of intra-MIS variability at PP5-6 will also contribute to better understanding the diversity of factors—environmental, economic, functional, and socio-cultural—that operate on finer-scales to influence technological behaviors.

Bifacial points and their by-products are notably absent in the PP5-6 lithic assemblage. At many other South African sites with MIS 4 deposits, lithic assemblages with high frequencies of bifacial points underlie lithic assemblages with high frequencies of backed pieces and blades (for reviews see [56, 178]). At many of these sites, the bifacial point-rich, SB-designated assemblages are chronometrically coeval with the SADBS and ALBS deposits at PP5-6 [98, 100]. Unlike SB-designated assemblages, the SADBS is characterized by a high-frequency of narrow backed pieces and small blades [22]. The ALBS analyzed sample from PP5-6 is currently too small to make generalizing techno-typological characterizations, but thus far backed pieces and bifacial points are absent, blades are relatively infrequent, and quartzite is the most common raw material type. The absence of bifacial points in the SADBS and ALBS levels of the PP5-6 assemblage is an anomaly worth further investigation; it further demonstrates the reality that every MSA site records a unique history [177] that could be a reflection of differing socio-cultural technological decisions, differing site functions, and/or differing local environmental adaptations.

The results presented here are consistent with previous reports that peaks in silcrete use during MIS 4 at PP5-6 correspond to modeled spikes in C4 vegetation [42]. However, a high frequency of silcrete also persists in MIS 3. It was hypothesized silcrete-use increased in MIS 4 due to different mobility strategies in response to different environmental conditions (pages 122–123 in [113]). The results presented in this paper better support a shift towards reduced residential mobility in MIS 4 compared to MIS 5 and MIS 3, and the shift in mobility is reflected by changes in flaking efficiency rather than a change in how much silcrete was used. If changing silcrete frequency is explained by changing availabilities of wood fuel required for heat treating silcrete [67, 116], then those availabilities must have also persisted into MIS 3. Further consideration of this hypothesis requires higher-resolution comparisons of the expanded technological record reported here to the Crevice Cave speleothem record, and to an expanded paleoenvironmental record that extends further into MIS 3.

Based on economic defendability theory, people respond to dense and predictable resources with elevated territoriality [71] and there is also commonly a decrease in mobility [75]. When the resource base focuses on predictable resources theory predicts ‘over-designed’ reliable hunting weapons [76]. Predictable and dense resources often provide greater carrying capacity and thus support larger populations and/or group sizes. Evidence for decreased mobility, tool functional changes, and larger populations or group sizes during the MIS 4 occupation at PP5-6 are consistent with a model of increased resource predictability and density during MIS 4 compared to MIS 5 and MIS 3 in this part of the south coast of South Africa. What may have caused this? During MIS 4 an unusual confluence of resources was presented to the hunter-gatherers occupying the site during the deposition of the SADBS, SGS, and OBS1. All three StratAggs have substantial amounts of mollusk remains along with the presence of an enlarged coastal plain [44] and an increase in high nutrient C4 grasses [42] that likely supported substantial populations of migratory ungulates. This novel juxtaposition of dense and predictable shellfish beds with the rich hunting grounds provided by a bi-annual and spatially constrained ungulate migration may have offered a startlingly rich resource base [41]. The seemingly implausible result was an increase in populations and occupation intensity during a glacial, and this drove some of the technological changes documented here in the stone tools. Further paleoenvironmental work is required to establish if this was the case. However, the results presented here do not support the proposed hypothesis that the technological characteristics of the HP reflect a response to food resource scarcity during MIS 4 [35].

MIS 4 was not homogenous. However, *intra*-MIS variability does not negate the larger scale pattern of *inter*-MIS variability. From a time-averaged perspective, global temperatures and sea-levels were on average lower during MIS 4, and this influenced the paleoenvironment at and around Pinnacle Point; during MIS 4, raw material availability differed, coastal resources were further away on average, and more C4 grasses covered the landscape on average. That was certainly not true for *all* of MIS 4, but it is an adequate abstraction, because our consideration of the lithic assemblage occurs at the same MIS-scale. From a time-averaged perspective, quartz frequencies are higher on average, edge length to mass ratio is higher on average, and silcrete outcrop cortex frequencies are higher on average during MIS 4. For human evolutionary studies, the MIS-scale analyses presented here are valuable for understanding long-term behavioral adaption, even though they necessarily homogenize complex data. Future work will focus on evaluating the relationship between environment and technology at finer scales and for individual environmental variables, and will permit us to address more specific questions about the relationship between Pleistocene environments and technologies. Currently, the age estimates of the sediments, the sea-level curves that underlie the coastline model, and the speleothem isotope curves are dated with different techniques with the result that they are not perfectly comparable at fine time scales to the lithic technological record. For example, the mean sea-level estimates generated by the paleoscape model [44] reported here do not take into account short-term fluctuations in coastline position, which would have changed the distance to rejuvenated quartzite cobble beaches. Some StratAggs in the MIS 4 levels at PP5-6 have substantial amounts of shellfish that may be explained by short sea level excursions not captured in the existing models. Current research is focused on developing higher-resolution and robust paleoenvironmental and paleoscape models for the region around PP5-6 [40] and when this is complete, we will be able to more precisely compare these records to changes in lithic technology at PP5-6 using the high-resolution database developed by the analyses presented here. To assess the extent to which our observations apply to glacial conditions more broadly could be achieved by integrating sequences that cover other glacial periods such as MIS 6 at Pinnacle Point 13B [77, 179], and MIS 2 at Boomplaas [180, 181] and Nelson Bay Cave [79, 182].

## Conclusion

The analysis presented here identified lithic technological traits that may reflect a response to Late Pleistocene glacial cycling with no *a priori* assumptions about what those traits will be or how the data are structured. The following qualities of the lithic assemblage are associated with glacial cycling because they are unique to the MIS 4 occupation at PP5-6 compared to the MIS 5 and MIS 3 occupations and they are expressed by a relatively large number of proxy traits: increased quartz, increased evidence for outcrop sources of quartzite and silcrete, increased evidence for earlier stages of reduction in silcrete, evidence for increased flaking efficiency in all raw material types, and changes in tool types and function for silcrete and quartz. Based on these results, it is hypothesized that humans responded to MIS 4 glacial environmental conditions at PP5-6 with increased population or group sizes, ‘place provisioning’, longer and/or more intense site occupations, and decreased residential mobility. Evidence at PP5-6 is not consistent with a climate-driven reduction in population size, shorter and less intense site occupations, or increased residential mobility during glacial conditions.

The extent to which this pattern applies beyond PP5-6 is unknown. Evidence for interconnectedness across major biomes in the form of coeval changes in raw material, tool types, and technological characteristics at some other sites [51, 172, 176] might suggest that similar changes occurred across much of South Africa. There is also evidence for spatial differences across South Africa, as indicated above, which may be the result of resource distributions in different regions having been affected differentially by glacial cycling. One solution is to conduct comparable analyses at multiple sites or site complexes that preserve a record of human behavior through MIS 5, MIS 4, and MIS 3. Some of these analyses must include sites outside the Cape Floristic Region.

By identifying the traits that associate with MIS and considering the potential causal mechanisms for these associations, this paper adds to our understanding of why lithic technology changes through time. Our results indicate that environment likely explains at least some lithic technological variability in the South African Pleistocene, but not all. This means that environment, adaptation, and economy, alongside socio-cultural factors, must be taken into account when comparing lithic assemblages. Technological differences across time and space in the South African MSA will not always indicate cognitive or socio-cultural changes, and no single mechanism will explain all the variability.

## Supporting information

S1 File Supplementary methods. List of coded lithic traits and definitions. Includes .cfg code for E4 program.
(DOCX)

S2 File Correspondence plots. Correspondence plots for all multivariate categorical data that exhibited a significant association with MIS (Chi^2^, p<0.05).
(PDF)

S3 File IntraMIS figures. Relative point density maps for key traits based on point elevation.
(PDF)

S1 Dataset Data tables. All data tables used for analysis. Includes trait frequencies and means, comparative statistics, and reference list for behavioral interpretations.
(XLSX)

S2 Dataset Raw data. Includes specimen numbers.
(XLSX)
