# Supplementary material for: Lithic technological responses to Late Pleistocene glacial cycling at Pinnacle Point Site 5-6, South Africa
Source: PLoS One. 2017 Mar 29;12(3):e0174051. doi: 10.1371/journal.pone.0174051 (PMC5371328; doi:10.1371/journal.pone.0174051)
Supplement: S1 File — List of coded lithic traits and definitions. Includes .cfg code for E4 program. (DOCX) [file pone.0174051.s001.docx]

This document provides the lithic traits and definitions recorded during the analysis of the Pinnacle Point 5-6 North Long Section.

Below is a list of the traits, definitions, references, and the conditions under which they were coded. There was no size cut-off for recording attributes. We encourage other lithic analysts studying Stone Age Africa to consider applying this coding scheme to enable large scale temporal and regional comparisons.

Traits were recorded using the open-source E4 data entry program designed by Shannon McPherron and Harold Dibble ([www.oldstoneage.com](http://www.oldstoneage.com)). The E4 software was used to speed up data entry and reduce transcription error. The code for using these traits in E4 can be found below. These should be cut and paste into the CFG file when starting E4. Alternatively, the text can be pasted into Notepad and saved as a .TXT file. The extension must then be changed to .CFG, which E4 can open. The CFG codes can be edited according to the characteristics of the assemblage and individual research questions.

The coding schemes for some traits were lumped for analysis and some traits were used to calculate ratios, indices, and estimates. The methods for those calculations are also indicated below.

List of recorded traits and their conditions (as coded in the CFG file), definitions, and references for additional details

| **Trait** | **Code** | **Condition** | **Menu** | **Definition** | **Reference for additional details** |
| --- | --- | --- | --- | --- | --- |
| Plotted Find Number | PlottedFindNumber | - | Enter Plotted Find Number | Unique ID number | - |
| Lot | Lot | - | Enter lot number | Smallest unit of provenience information | - |
| Excavated with Residue Protocol |  | - | Yes | Whether artifact was excavated with residue protocol or not. | - |
|  | ExcavatedWithResidueProto |  | No |  | - |
| Washed | Washed | - | Yes | Whether artifact was washed or not. We used detergent and ultrasonic cleaner. Calgon was used for calcretions when necessary. | - |
|  |  |  | No |  | - |
| Researcher | Researcher | - | Select the Researcher: | Name of researcher analyzing lithic | - |
| Raw Material | RawMaterial |  | Menu will depend on local raw material availability |  |  |
| Lithic Artifact Class | LithicArtifactClass |  | Compflake | Complete flake with bulb, platform, distal end, clear dorsal and ventral surfaces and no major breaks | - |
|  |  |  | FlakeFrag | Incomplete flake, with clear dorsal and ventral surfaces. Bulb and platform may or may not be present, flake is broken |  |
|  |  |  | BladeBladeFrag | Blade or blade fragment. Blank length is twice the width (this has to be estimated for fragments). Parallel or nearly parallel dorsal scars and lateral edges are expected. If in doubt go with flakefrag |  |
|  |  |  | Shatter | Artifact with no bulb and it is not possible to distinguish clear dorsal side and ventral surfaces. It should be chosen for all pieces in doubt |  |
|  |  |  | RetouchedPiece | Any piece with retouch - fairly continuous series of small removals along an edge |  |
|  |  |  | Core | Any piece that has had one or more blank removals (at least one negative bulb of percussion is visible) |  |
|  |  |  | HammerManuport Grindstone | Lithic artifact that has not been knapped |  |
| Unretouched Point | UnretouchedPoint? | Condition1=LithicArtifactClass CompFlake FlakeFrag BladeBladeFrag | Yes | Lateral edges converge to a point | - |
|  |  |  | No | Lateral edges do not converge to a point |  |
|  |  |  | Indeterminate | Difficult to classify |  |
| Retouched Piece Blank | RetouchedPieceBlank | Condition1=LithicArtifactClass RetouchedPiece | Flake | Retouched tool was most likely manufactured on flake blank | - |
|  |  |  | Blade | Retouched tool was most likely manufactured on blade blank |  |
|  |  |  | ShatterIndeterminate | Retouched tool was most likely manufactured on shatter or other kind of knapping debris or it is difficult to assign to "Flake" or "Blade".If in doubt, go with "Shatter or indeterminate" |  |
| Completeness | Completeness | Condition1=LithicArtifactClass NOT CompFlake Shatter Core HammerManuportGrindstone | Complete | Complete artifact is present (also acceptable with some minor snaps or damage) | - |
|  |  |  | Proximal | Preserves complete bulb and platform but not the distal end |  |
|  |  |  | Distal | Distal portion of detached piece, no platform or bulb |  |
|  |  |  | Mesial | Mesial portion of detached piece. No platform, bulb, or distal end |  |
|  |  |  | Fragment | Fragment - not possible to say with confidence which part of the flake it represents |  |
|  |  |  | Left lateral | Preserves left portion of platform and bulb, flake or blade is split along flaking axis |  |
|  |  |  | Right lateral | Preserves right portion of platform and bulb, flake or blade is split along flaking axis |  |
|  |  |  | ProxLeftLat | Proximal left lateral |  |
|  |  |  | ProxRightLat | Proximal right lateral |  |
|  |  |  | DistalLeftLat | Distal left lateral |  |
|  |  |  | DistalRightLat | Distal right lateral |  |
|  |  |  | MesialLeftLat | Medial left lateral |  |
|  |  |  | MesialRightLat | Medial right lateral |  |
| Core or Hammer Completeness | CoreHammerCompleteness | Condition1=LithicArtifactClass Core HammerManuportGrindstone | Complete | Complete core or hammer/grindstone/manuport | - |
|  |  |  | SplitFragment | Split or Fragment. A part of a core or hammer/grindstone/manuport that appears to have split or broke |  |
| Evidence for Bipolar Percussion | EvidenceBipolarPercussion | Condition1=LithicArtifactClass CompFlake Core HammerManuportGrindstone OR  Condition2=Completeness Complete LeftLateral RightLateral SplitHammerCobble | Yes | Artifact has two clear opposed bulbs on the ventral surface, or two opposed negative bulbs within a single dorsal or flake scar | - |
|  |  |  | No | Artifact does not have any indication of being a product of bipolar percussion |  |
|  |  |  | Indeterminate | Artifact is difficult to classify - maybe bipolar |  |
| Blade Technical Category | TechnicalCategory | Condition1=LithicArtifactClass BladeBladeFrag | Enter the blade technical category (i.e., B1, B2, B3, etc…) | Type definitions are from Soriano et al. 2007 and Villa et al. 2005, except that a ‘P’ is inserted into the category acronym if the piece exhibits pre-heat treatment surface, rather than a cortical surface (i.e. AP1, AP2, etc.) | Soriano et al., 2007; Villa et al., 2005 |
| Cortex Area | CortexArea |  | 0% | Enter the Estimated Cortex Area of DORSAL surface, or the entire surface of Shatter Core and hammermanuportgrindstone | Brown, 2011 |
|  |  |  | 1-20% |  |  |
|  |  |  | 21-40% |  |  |
|  |  |  | 41-60% |  |  |
|  |  |  | 61-80% |  |  |
|  |  |  | 81-99% |  |  |
|  |  |  | 100% |  |  |
| Platform Cortex | PlatformCortex | Condition1=LithicArtifactClass CompFlake OR  Condition2=Completeness Complete Proximal LeftLateral RightLateral ProxLeftLat ProxRightLat | YesComplete | Platform is completely cortical | - |
|  |  |  | YesPartial | Platform is partially cortical |  |
|  |  |  | No | There is no cortex on the platform |  |
| Cortex Chatter Marks | CortexChatterMarks | Condition1=CortexArea NOT 0% OR  Condition2=PlatformCortex YesComplete YesPartial | Yes | Chatter marks | - |
|  |  |  | No | No chatter marks present |  |
|  |  |  | Indeterminate | Cortex has marks but it is not possible to determine if there are clear chatter marks or not. If in doubt, go with indeterminate |  |
| Cortex Roundness | CortexRoundness | Condition1=CortexArea NOT 0% OR  Condition2=PlatformCortex YesComplete YesPartial | Angular | Applies only to cortical surface and any apparent edges that are present and not to the shape of the overall core. | - |
|  |  |  | SubangularSubrounded |  |  |
|  |  |  | Rounded |  |  |
|  |  |  | Indeterminate | There is no edge (cortical part of piece is flat) or it is difficult to classify |  |
| Cortex Location | CortexLocation | Condition1=CortexArea NOT 0% AND  Condition2=LithicArtifactClass NOT Shatter Core HammerManuportGrindstone | See “Completeness” categories above. | Indicate where the majority of cortex is present in the dorsal surface. See “Completeness” categories above. | - |
| Cortex Type | CortexType | Condition1=CortexArea NOT 0% OR  Condition2=PlatformCortex YesComplete YesPartial | Cobble | Rounded water-worn cortex |  |
|  |  |  | Outcrop | Irregular, rough cortex |  |
|  |  |  | Indeterminate | It is not possible to determinate if it is cobble or outcrop cortex |  |
| Visible Heat Treatment Luster | VisibleLuster | Condition1=RawMaterial Silcrete | Yes | Silcrete piece has luster suggestive of heat treatment | Brown, 2011; Brown et al., 2009 |
|  |  |  | No | Silcrete piece does not have luster. It has matte and rough surface |  |
|  |  |  | Indeterminate | It is not possible to determinate if the artifact has luster or not |  |
| Pre-Heat Treatment Area | PreHeatArea | Condition1=RawMaterial Silcrete AND  Condition2=VisibleLuster Yes | 0% | Amount of pre-heat treatment surface on dorsal surface of flakes, blades and blanks of retouched pieces. For shatter without distinguishable dorsal or ventral faces and cores it is the amount of pre-heat treatment surface on the entire surface (not just dorsal) | - |
|  |  |  | 1-20% |  |  |
|  |  |  | 21-40% |  |  |
|  |  |  | 41-60% |  |  |
|  |  |  | 61-80% |  |  |
|  |  |  | 81-99% |  |  |
|  |  |  | 100% |  |  |
| Platform Pre-Hea treatment Surface | PlatformPreHeatSurface | Condition1=RawMaterial Silcrete AND  Condition2=VisibleLuster Yes AND  Condition3=LithicArtifactClass CompFlake OR  Condition4=Completeness Complete Proximal LeftLateral RightLateral ProxLeftLat ProxRightLat | YesComplete | Platform is completely pre-heat treated | - |
|  |  |  | YesPartial | Platform is partially pre-heat treatment |  |
|  |  |  | Absent | There is no pre-heat treated surface on the platform |  |
| Pre-Heat Treatment Location | PreHeatTreatmentLocation | Condition1=RawMaterial Silcrete AND  Condition2=VisibleLuster Yes AND  Condition3=PreHeatArea NOT 0% AND  Condition4=LithicArtifactClass NOT Shatter Core HammerManuportGrindstone | See “Completeness” categories above. | Describe where majority of pre-heat treated surface is present in the dorsal surface, using the same criteria as for cortex. See “Completeness” categories above. | - |
| Evidence of Postdepositional Burning | EvidencePostDepBurning |  | None | No evidences of post-depositional burning |  |
|  |  |  | PotLidScar | Artifact has negative scars of podlits |  |
|  |  |  | PieceISPotlid | The artifact is a potlit |  |
|  |  |  | Crazing | The artifact shows a network of fine cracks |  |
|  |  |  | ColorChange | The artifact shows changes in color due to burning |  |
|  |  |  | CombinationOfFeatures | The artifact shows a combination of two or more of the previouse features related with burning |  |
| Dorsal Scar Count | DorsalScarCount | Condition1=LithicArtifactClass NOT Shatter Core HammerManuportGrindstone AND  Condition2=CortexArea NOT 100% | - | Number of dorsal flake scars greater than 6 mm in maximum length | - |
| Dorsal Direction | DorsalDirection | Condition1=LithicArtifactClass NOT Shatter Core HammerManuportGrindstone AND  Condition2=RetouchedPieceBlank NOT ShatterIndeterminate AND  Condition3=CortexArea NOT 100% AND  Condition4=DorsalScarCount NOT 0 1 | Radial | Three or more scars from at least three different directions | - |
|  |  |  | Subradial | Two or more scars from different non-opposed directions |  |
|  |  |  | Bidirectional | Two or more scars from two opposed directions |  |
|  |  |  | Unidirectional | Two or more scars from one direction |  |
|  |  |  | BiOrUni | Bidirectional or unidirectional. Two or more scars from parallel directions but it is not possible to determine whether they are bidirectional or unidirectional |  |
|  |  |  | Indeterminate | Difficult to classify |  |
| Aris Orientation | ArisOrientation | Condition1=DorsalDirection Bidirectional Unidirectional BiOrUni AND  Condition2=CortexArea NOT 100% AND  Condition3=DorsalScarCount NOT 0 1 2 | Parallel | Arisses are parallel or sub-parallel | - |
|  |  |  | Convergent | Arrisses are converge |  |
|  |  |  | Indeterminate |  |  |
| Profile Shape | ProfileShape | Condition1=LithicArtifactClass CompFlake OR  Condition2=Completeness Complete Proximal LeftLateral RightLateral ProxLeftLateral ProxRightLat | Flat | The ventral face form the end of the bulb to the distal end is flat and completely or almost completely touches a flat surface | - |
|  |  |  | Curved | The ventral face is curved, makes an arch and does not touches a flat surface |  |
|  |  |  | Twisted | The ventral surface curls and often the distal end is oblique relating the proximal end |  |
|  |  |  | Indeterminate | It is not possible to determine if the profile is flat, curved or twisted |  |
| Mass | Mass |  | - | Weight in grams | - |
| Maximum Length | MaxLength |  | - | Maximum dimension | - |
| Maximum Width | MaxWidth |  | - | Maximum dimension perpendicular to maximum length | - |
| Technological Length | TechLength | Condition1=LithicArtifactClass CompFlake OR  Condition2=Completeness Complete LeftLateral RightLateral | - | Length from percussion point (or center of platform if not visible) to the distal end of the blank along flaking axis. In cores, the length is measured along axis of last flake/blade removal on surface with the most flake scars | Debénath and Dibble, 1994:19, method 3 |
| Maximum Technological Width | MaxTechWidth | Condition1=LithicArtifactClass CompFlake OR  Condition2=Completeness Complete Proximal Mesial | - | Maximum width perpendicular to the technological length | Debénath and Dibble, 1994:19, method 3 |
| Maximum Thickness | MaxThickness |  | - | Maximum thickness including bulb | - |
| Mid-point Thickness | MidThickness | Condition1=LithicArtifactClass CompFlake OR  Condition2=Completeness Complete LeftLateral RightLateral | - | Thickness at midpoint of technological length | - |
| Platform Width | PlatformWidth | Condition1=LithicArtifactClass CompFlake OR  Condition2=Completeness Complete Proximal | - | The distance between two points at which the striking platform intersects the ventral surface | Debénath and Dibble, 1994:19, method 3 |
| Platform Thickness | PlatformThickness | Condition1=LithicArtifactClass CompFlake OR  Condition2=Completeness Complete Proximal LeftLateral RightLateral ProxLeftLat ProxRightLat | - | The distance between the point of percussion and the opposite point on the dorsal edge of the striking platform and the flake dorsal surface | Debénath and Dibble, 1994:19 |
| Exterior Platform Angle | ExteriorPlatAngle | Condition1=LithicArtifactClass CompFlake OR  Condition2=Completeness Complete Proximal LeftLateral RightLateral ProxLeftLat ProxRightLat | - | Angle of platform and dorsal surface take at platform midpoint and with the arm of the goniometer parallel to flake profile | Dibble, 1997 |
| Flake Termination | FlakeTermination | Condition1=LithicArtifactClass CompFlake OR  Condition2=Completeness Complete Distal DistalLeftLat DistalRightLat | Feather | Acute angle, sharp | Andrefsky, 2005:21 |
|  |  |  | PartialHingeOrStep | Partial hinge/step . Termination is s-shaped in profile. In profile, termination is hinged or stepped on the ventral side and is feathered on the dorsal side |  |
|  |  |  | Hinge | Curved up towards dorsal surface. |  |
|  |  |  | Step | Abrupt right angle break |  |
|  |  |  | HingeorStep | Difficult to classify if it is either hinge or step |  |
|  |  |  | Overshoot | Preserves core relict edge or opposite platform, plunging or not plunging |  |
|  |  |  | Indeterminate | Termination is difficult to classify, or the artifact is retouched at distal end |  |
| Platform Preparation | PlatformPrep | Condition1=LithicArtifactClass CompFlake OR  Condition2=Completeness Complete Proximal LeftLateral RightLateral ProxLeftLat ProxRightLat AND  Condition3=PlatFormCortex NOT YesComplete | NotPrepared | Plain platform, with only a single scar | Soriano et al., 2007 |
|  |  |  | FacetedWithBulb | Faceted. More than one scar, negative bulbs of percussion are visible within at least one of them |  |
|  |  |  | ResidualFacetWithoutBulb | Residual facets. More than one scar, negative bulbs of percussion are not visible |  |
|  |  |  | Indeterminate | Difficult to classify, may be due to crushing or very small thin size |  |
| Number of Platform Scars | NumberPlatformScars | Condition1=LithicArtifactClass CompFlake OR  Condition2=Completeness Complete Proximal LeftLateral RightLateral ProxLeftLat ProxRightLat AND  Condition3=PlatformPrep NOT NotPrepared Indeterminate | Enter number of platform scars (greater than 1mm) | - | - |
| Dorsal Preparation | DorsalPrep | Condition1=LithicArtifactClass CompFlake OR  Condition2=Completeness Complete Proximal LeftLateral RightLateral ProxLeftLat ProxRightLat | None | No small scars off the platform on the dorsal face | Soriano et al., 2007 |
|  |  |  | HingedRemovals | Hinged removals . A cluster of small removals on the dorsal face with negative bulbs and hinged or stepped terminations that initiate of the platform |  |
|  |  |  | LongFeatherRemovals | Long feather terminating removals . A cluster of elongated feather-terminating removals with negative bulbs and hinged or stepped terminations that initiate of the platform |  |
|  |  |  | LateralNotching | LateralNotching. Two clusters of small removals with negative bulbs and hinged or stepped terminations that initiate of the platform- one cluster near each lateral edge |  |
|  |  |  | Indeterminate | Difficult to classify |  |
| Platform Abrasion | PlatAbrasion | Condition1=LithicArtifactClass CompFlake OR  Condition2=Completeness Complete Proximal LeftLateral RightLateral ProxLeftLat ProxRightLat | No | No visible platform abrasion | Soriano et al., 2007 |
|  |  |  | Yes | Some visible platform abrasion |  |
|  |  |  | Indeterminate |  |  |
| Fracture Initiation Point | FractureInitiationPoint | Condition1=LithicArtifactClass CompFlake OR  Condition2=Completeness Complete Proximal | NonExpressed | Fracture initiation is not expressed | Soriano et al., 2007 |
|  |  |  | Centered | Fracture initiated roughly in center of platform |  |
|  |  |  | Lateral | Fracture initiated significantly off-center |  |
|  |  |  | Indeterminate | Difficult to determine where fracture initiated |  |
| Platform Delineation | PlatformDelineation | Condition1=LithicArtifactClass CompFlake OR  Condition2=Completeness Complete Proximal LeftLateral RightLateral ProxLeftLat ProxRightLat | RegCurve | Regular curve .Platform on same plane as flake and bulb | Soriano et al., 2007:689 |
|  |  |  | OverhangCurveNoBreak | Curved, "overhanging", and no break in delineation . Platform plane is more dorsal than rest of flake, delineation between platform and ventral surface is smooth |  |
|  |  |  | OverhangCurveBreak | Curved, "overhanging", and significant break in delineation. Delineation has a bump that sticks out where percussion point is |  |
|  |  |  | Double | Image B4, There are two obvious percussion points |  |
|  |  |  | Rectilinear | Image B5, Delineation is not curved, but straight line |  |
|  |  |  | Indeterminate | Image B6, Difficult to classify or irregular |  |
| Fissuring on Platform | FissuringOnPlatform | Condition1=LithicArtifactClass CompFlake OR  Condition2=Completeness Complete Proximal LeftLateral RightLateral ProxLeftLat ProxRightLat | None | No visible fissure around percussion point | Soriano et al., 2007:689 |
|  |  |  | CompleteFissure | Visible fissure around break in delineation at percussion point |  |
|  |  |  | PartialFissure | Partial fissure on one side of break in delineation at percussion point |  |
|  |  |  | Indeterminate | Difficult to classify |  |
| Marks on Ventral Surface | MarksVentralSurface | Condition1=LithicArtifactClass CompFlake OR  Condition2=Completeness Complete Proximal LeftLateral RightLateral ProxLeftLat ProxRightLat | None | No ventral marks visible | Soriano et al., 2007:689 |
|  |  |  | RipplesOrUndulations | Concentric ripples or other kind of significant undulations in surface |  |
|  |  |  | ShatteredBulb | Bulb is split along the plane of detachment so that a scar with hinge or step termination is removed from the bulbar surface. Differs from eraillure scars, which a small and feather-terminated |  |
|  |  |  | Hertzian cone | Visible contour of hertzian cone |  |
|  |  |  | Crushing | Crushing at percussion point |  |
|  |  |  | Indeterminate | Difficult to classify or other, but there is a mark present |  |
| Lipping | Lipping | Condition1=LithicArtifactClass CompFlake OR  Condition2=Completeness Complete Proximal LeftLateral RightLateral ProxLeftLat ProxRightLat | NoLip | No lip | Soriano et al., 2007:694 |
|  |  |  | SmallOrPartialLIp | Small or partial lip |  |
|  |  |  | LargeContinuousLip | Large lip that extends across majority of platform |  |
|  |  |  | Indeterminate |  |  |
| Bulb Presence | Bulb | Condition1=LithicArtifactClass CompFlake OR  Condition2=Completeness Complete Proximal LeftLateral RightLateral ProxLeftLat ProxRightLat | Yes | Bulb is visible | - |
|  |  |  | No | No bulb is visible (even though there is a clear platform) |  |
|  |  |  | Indeterminate |  |  |
| Bulb Length | BulbLength | Condition1=Bulb Yes | - | Length of bulb (mm) from percussion point to furthest extent on ventral face | - |
| Platform Morphology | PlatformMorphology | Condition1=LithicArtifactClass CompFlake OR  Condition2=Completeness Complete Proximal LeftLateral RightLateral ProxLeftLat ProxRightLat | NarrowLinear | Narrow and linear platform, see image D2 | Soriano et al., 2007:689 |
|  |  |  | QuadrangularTrapezoidal | Quadrangular or trapezoidal . Broad thick platform, see image D5 |  |
|  |  |  | OvularOrTriangular | Ovular or triangular . Restricted platform , width is less than width of flake, see image D3 |  |
|  |  |  | NarrowCurved | Curved platform, see image D4 |  |
|  |  |  | Punctiform | Punctiform . Very small platform, see image D1 |  |
|  |  |  | ChapeauDeGendarme | Chapeau de gendarme . Unique kind of platform with this shape and lots of faceting |  |
|  |  |  | Indeterminate | Difficult to classify |  |
| Edge Damage | EdgeDamage | Condition1=LithicArtifactClass NOT Shatter Core HammerManuportGrindstone AND  Condition2=Completeness NOT Fragment | Yes | Small, generally isolated flake scars and/or snaps are present on edges | - |
|  |  |  | No | Edge shows no damage |  |
|  |  |  | Indeterminate | Difficult to classify |  |
| Diagnostic Impact Fractures | DiagnosticImpactFractures | Condition1=EdgeDamage Yes | None | No DIFS present, though there may be snaps or other types of breaks that are not 'diagnostic' | Fischer et al., 1984 |
|  |  |  | StepTerminatingBendingFracture | Step-terminating fractures end abruptly and meet the surface of the flake at a right angle (Hayden 1979). Bending fractures initiate without the formation of a Hertzian cone and consequently lack a negative bulb of percussion. |  |
|  |  |  | UnifacialSpinOff 6mm | Unifacialspinoff>6mm,Spin-off fractures are cone fractures that initiate off bending fractures. Unifacial spin-off fractures occur only on one face, not  both. These fractures need to be more than 6 mm in max length to be considered difs (Fischer et al. 1984). |  |
|  |  |  | BifacialSpinOff | Bifacialspinoff, Multiple spin-off fractures that initiate off both faces of a bending fracture. |  |
|  |  |  | ImpactBurinationNoBulb | Impact burinations resemble a burin blow occurring along either one of the lateral edges, but lack the negative bulb of percussion common to deliberate burination. |  |
|  |  |  | MoreThanOneTypeOf DIF | More than one type of DIF is present. These will be coded later in a seperate analysis. |  |
| Banding Orientation | BandingOrientation | Condition1=RawMaterial Quartzite AND  Condition2=LithicArtifactClass CompFlake OR  Condition3=Completeness Complete Proximal Distal LeftLateral RightLateral ProxLeftLat ProxRightLat DistalLeftLat DistalRightLat MesialLeftLat MesialRightLat | NoBandingVisible | No Banding Visible | Wilkins, 2013 |
|  |  |  | With | Bands will be visible on dorsal and ventral surfaces and parallel to flaking axis |  |
|  |  |  | Through | Bands are mainly visible in side view and run parallel to the flaking axis |  |
|  |  |  | Oblique | Bands are visible on the dorsal and ventral surfaces and are diagonal to the flaking axis |  |
|  |  |  | Against | Bands are visible on dorsal and lateral surfaces and are perpendicular to flaking axis |  |
|  |  |  | Indeterminate | Difficult to classify |  |
| Retouch Type | RetouchType | Condition1=LithicArtifactClass RetouchedPiece | NotchDenticulate | Notch or Denticulate. One or more convex half-moon modification to edge that was prepared with at least two small flake removals, or denticulation is regular and exaggerated (snaps or single flake removals are not considered notches but should be coded as edge damage) | - |
|  |  |  | Backing | Blunted edge(s) - very steep retouch, perpendicular to plane of tool |  |
|  |  |  | Burination | Probably rare - must have negative bulb and located along a lateral edge |  |
|  |  |  | InvasiveEdgeShaping | Invasive edge shaping. Long flake scars extend into body of tool |  |
|  |  |  | MarginalEdgeShaping | Marginal edge shaping. Short flake scars that do not extend into body of tool |  |
|  |  |  | *PiecesEsquillees* | *Pieces esquillees* . Wedge shaped piece with several small stepped scars imitating from two opposed chisel-like edges | Villa et al., 2012 |
| Retouch Edge Angle | RetouchEdgeAngle | Condition1=RetouchType NotchDenticulate Backing InvasiveEdgeShaping MarginalEdgeShaping | Enter angle of retouch. | Angle of retouched edge (measure center of longest series of continuous retouch), or from inside the largest notch | - |
| Diameter of Largest Notch | DiameterOfLargestNotch | Condition1=RetouchType NotchDenticulate | Enter the Diameter of the Largest Notch (mm): | Diameter of largest notch on tool | - |
| Retouched Piece Typology | RetouchedPieceTypology | Condition1=LithicArtifactClass RetouchedPiece | BackedPiece | Backed piece | - |
|  |  |  | NotchedPiece | Notched piece – notches must have multiple flake scars, notch or denticulate. |  |
|  |  |  | RetouchedPoint | Converging edges shaped into a point |  |
|  |  |  | SideScraper | Side scraper. Continuous retouch along a portion of one lateral edge |  |
|  |  |  | EndScraper | End scraper. Continuous retouch that includes distal end |  |
|  |  |  | DoubleScraper | Double scraper. Continuous retouch along two edges that do not converge or converge into a very thick or irregular point (would not be classified as a point) |  |
|  |  |  | ScraperWithThreeOrMoreEdges | Scraper with three or more edges retouched |  |
|  |  |  | PiecesEsquillees | *Pieces esquillees* . Wedge shaped piece with several small stepped scars imitating from two opposed chisel-like edges |  |
|  |  |  | MinimallyRetouched | Retouch is limited or edge damage may have resulted from use, difficult to classify because there is so little retouch |  |
|  |  |  | Indeterminate | Difficult to classify because irregular or broken, but could be one of above categories |  |
|  |  |  | Other | Does not fit above category, but a typological designation is possible (i.e., transverse scraper, tanged tool, any other thing that might surprise us), if you pick this you will have to call it something in the next field |  |
| Other Retouched Piece Typology | OtherRetouchedPieceTypology | Condition1=RetouchedPieceTypology Other | Please enter other retouched piece typology | Option to write in other typology if "other" is selected (e.g. awl) | - |
| Number of Core Faces | NumberCoreFaces | Condition1=LithicArtifactClass Core | One | One face with one or more removals. Face 1 will be the largest and/or most exploited face. | NOTE: Each core is divided into its number of faces. Individual attributes are recorded for each of those attributes. |
|  |  |  | Two | Two faces with one or more removals. Face 1 will be the largest and/or most exploited face. Face 2 will be the next largest and/or most exploited face. |  |
|  |  |  | Three | Three faces with one or more removals. Face 1 will be the largest and/or most exploited face. Face 2 will be the next largest and/or most exploited face. Face 3 will be the third largest and/or most exploited face. |  |
|  |  |  | Four | Four faces with one or more removals. Three faces with one or more removals. Face 1 will be the largest and/or most exploited face. Face 2 will be the next largest and/or most exploited face. Face 3 will be the third largest and/or most exploited face. Face 4 will be the fourth largest and/or most exploited face. |  |
|  |  |  | MoreThanFour | More than four faces with one or more removals. Details for only the first four faces will be recorded in this database. |  |
| Face X Cortex Area | FaceXCortexArea | Condition1=LithicArtifactClass Core AND  Condition2=CortexArea NOT 0% | 0% | Amount of cortex on core face | - |
|  |  |  | 1-20% |  |  |
|  |  |  | 21-40% |  |  |
|  |  |  | 41-60% |  |  |
|  |  |  | 61-80% |  |  |
|  |  |  | 81-99% |  |  |
|  |  |  | 100% |  |  |
| Face X Pre-Heat Treatment Area | FaceXPreHeatArea | Condition1=LithicArtifactClass Core AND  Condition2=RawMaterial Silcrete AND  Condition3=VisibleLuster Yes AND  Condition4=PreHeatArea NOT 0% | 0% | Amount of pre-heat treatment surface on each core face |  |
|  |  |  | 1-20% |  |  |
|  |  |  | 21-40% |  |  |
|  |  |  | 41-60% |  |  |
|  |  |  | 61-80% |  |  |
|  |  |  | 81-99% |  |  |
|  |  |  | 100% |  |  |
| Face X Dorsal Scar Count | FaceXDorsalScarCount | Condition1=LithicArtifactClass Core | - | Number of dorsal flake scars greater than 6 mm in maximum length | - |
| Face X Dorsal Direction | FaceXDorsalDirection | Condition1=LithicArtifactClass Core AND  Condition2=Face1DorsalScarCount NOT 0 1 | Radial | Three or more scars from at least three different directions | - |
|  |  |  | Subradial | Two or more scars from different non-opposed directions |  |
|  |  |  | Bidirectional | Two or more scars from two opposed directions |  |
|  |  |  | Unidirectional | Two or more scars from one direction |  |
|  |  |  | BiOrUni | Two or more scars from parallel directions but it's not possible to determine whether they are bidirectional or unidirectional |  |
|  |  |  | Indeterminate | Difficult to classify |  |
| Face X Aris Orientation | FaceXArisOrientation | Condition1=LithicArtifactClass Core AND  Condition2=Face1DorsalDirection NOT Radial Subradial Indeterminate NA | Parallel | Arisses more or less parallel | - |
|  |  |  | Convergent | Arrisses converge |  |
|  |  |  | Indeterminate |  |  |
| Face X Core Exploitation | FaceXCoreExploitation | Condition1=LithicArtifactClass Core | Recurrent | Multiple removals of relatively similar size | Boëda, 1995 |
|  |  |  | Preferential | One large removal that other removals may have been used to prepare for |  |
|  |  |  | Platform | This face served mainly as the platform for exploitation from a different core surface |  |
| Face X Termination of Final Removal | FaceXTerminationFinalRemoval | Condition1=LithicArtifactClass Core | Feather | Acute angle, sharp |  |
|  |  |  | PartialHIngeOrStep | In profile, termination hinged or stepped on the ventral side and is feathered on the dorsal side |  |
|  |  |  | Hinge | Curved up towards the dorsal surface |  |
|  |  |  | Step | Abrupt right angle break |  |
|  |  |  | HingeOrStep | Difficult to classify as either hinge or step, but one of the two |  |
|  |  |  | Overshoot | Preserves core relict edge or opposite platform, plunging or not plunging |  |
|  |  |  | Indeterminate | Terminations that are difficult to classify, or piece is retouched at distal end |  |
| Face X Length of Final Removal | FaceXLengthOfFinalRemoval | Condition1=LithicArtifactClass Core | - | Length from bulb of percussion to distal termination | - |
| Face X Width of Final Removal | FaceXWidthOfFinalRemoval | Condition1=LithicArtifactClass Core | - | The technological width is perpendicular to technological length | - |
| Face X Remnant Platform of Final Removal | FaceXRemnantPlatformOfFinalRemoval | Condition1=LithicArtifactClass Core | NotPrepared | Final remnant platform is not prepared | - |
|  |  |  | Faceted | Final remnant platform is faceted |  |
|  |  |  | Cortical | Final remnant platform is cortical |  |
|  |  |  | Indeterminate | - |  |
| Core Blank Sphericity | CoreBlankSphericity | Condition1=LithicArtifactClass Core | SphericalOrCubic | Estimated core blank shape is sherical/cubical nodule that would have had a low surface area to volume ratio | - |
|  |  |  | NotSphericalFlat | Estimated core blank shape is flat tabular nodule that would have had a high surface area to volume ratio |  |
|  |  |  | Flake | Probably a flake blank based on visible or partially visible bulbar surface |  |
|  |  |  | Indeterminate | If in doubt go with indeterminate |  |
| Intended Product | IntendedProduct | Condition1=LithicArtifactClass Core | Flake | Intended products are not blades or points |  |
|  |  |  | BladeBladelet | Flake scar removals resemble blade or bladelet (length is twice the width) |  |
|  |  |  | Point | Ridges of flake scar converge where a relatively large triangular product was detached |  |
|  |  |  | Indeterminate | - |  |
| Volman Core Type One | VolmanTypeOne | Condition1=LithicArtifactClass Core | CoreOnFlake | Core on flake. Flake with clear flake removals removed after original detachment | Volman, 1981 |
|  |  |  | MinimalCore | Minimal core. Only a few irregularly oriented flake removals |  |
|  |  |  | CorePrepforOneMajor | Core prepared for one major removal. Set-up for the production of a single flake |  |
|  |  |  | CoreForIntersectingScars | Core prepared for intersecting scars. Core for the production of flakes with intersecting scars (i.e. flakes come from multiple directions) |  |
|  |  |  | CoreForParallelConvScars | Core prepared for parallel or converging scars. Flake scars from a platform tend to run in a single direction or opposed directions |  |
|  |  |  | Cylinder Core | Cylinder core. Removals around the entire core surface from a single or two opposed platforms |  |
| Volman Core Type for Intersection Scars | VolmanCoreForIntersectingScars | Condition1=LithicArtifactClass Core AND  Condition2=VolmanTypeOne CoreForIntersectingScars | ChangeOrientation | Change of orientation core. Three or more striking platforms oriented on different planes with respect to one another, with intersecting removals on one or more planes | Volman, 1981 |
|  |  |  | RadialCore | Radial core. Flake removals directed from the circumference towards the center of one surface or towards the centers of two opposed surfaces |  |
|  |  |  | AdjacentPlatformCore | Adjacent platform core. Flaked to two opposed surfaces from only a portion of the circumference |  |
| Volman Core Type for Parallel Converging Scars | VolmanCoreForParallelConvScars | Condition1=LithicArtifactClass Core AND  Condition2=VolmanTypeOne CoreForParallelConvScars | SinglePlat | Single platform core. One major platform from which several flakes were struck in the same direction | Volman, 1981 |
|  |  |  | OpposedPlatSameSide | Opposed platform same side core. Two major platforms, opposed to one another in the same plane |  |
|  |  |  | OpposedPlatOppSide | Opposed platform opposite side core. Two major platforms, opposed to one another on parallel or subparallel planes |  |
|  |  |  | OpposedPlatSameAndOppSide | Opposed platform on same and opposite side. Two opposed platforms on one plane and one, or two opposed, platform(s) oriented in the same direction on a parallel or sub-parallel plane |  |
|  |  |  | OtherDoubletPlat | Other double platform core. Core with two major platforms not oriented in the same, parallel or sub-parallel planes |  |
| Conard Unified Core Type | ConardUnifiedType | Condition1=LithicArtifactClass Core | Inclined | Two opposed surfaces with removals that are angled with respect to the plane of intersection, removals are on broad surfaces and converge toward center. | Conard et al., 2004 |
|  |  |  | Parallel | Two opposed surfaces with removals that parallel or nearly parallel with plane of intersection, removals are on broad surface and may converge towards center, near distal end, or be parallel to each other. |  |
|  |  |  | Platform | Volume defined by more than two surfaces with removals parallel to each other. Main removals are from a well-defined striking platform. Often removals are from a narrow surface. |  |
|  |  |  | Other | - |  |
| Site Specific Core Typology | SiteSpecificCoreTypology | Condition1=LithicArtifactClass Core | Enter site specific core typology |  |  |
| Completeness of Backed Pieces | CompletenessOfBackedPiece | Condition1=RetouchedPieceTypology BackedPiece | Complete |  | - |
|  |  |  | Fragment |  |  |
| Backed Piece Typology | BackedPieceTypology | Condition1=RetouchedPieceTypology BackedPiece | Segment | Curved backed edge, can be completely or partially backed | Inizan et al., 1999; Porraz et al., 2013; Soriano et al., 2007; Villa et al., 2010 |
|  |  |  | Triangle | Two backed edges converge to point |  |
|  |  |  | Trapeze | Completely backed by definition, piece is trapezoid in form (essentially a double truncation with backing also along the blade edge between the truncations) |  |
|  |  |  | TruncationSingle | Single truncation. Distal or proximal portion of blade is ‘truncated’, back is left unretouched, by definition the piece is partially backed |  |
|  |  |  | TruncationDouble | Double truncation. Distal and proximal portion of blade is truncated, back is left unretouched, by definition the piece is partially backed |  |
|  |  |  | TruncationInd | Piece is broken so it is impossible to tell whether it’s double or single truncation |  |
|  |  |  | MinimallyBacked | Minimally backed. Low amount of retouch |  |
|  |  |  | Indeterminate | Piece is too fragmented to classify |  |
|  |  |  | Other | The artifact is another type of backed piece that is not present in the menu. |  |
| Other Backed Piece Typology | OtherBackedPieceID | Condition1=BackedPieceTypology Other | Identify the other backed piece typology: |  | - |
| Backing Distribution | BackingDistribution | Condition1=RetouchedPieceTypology BackedPiece | CompletelyBacked | Completely backed. One entire lateral edge of blade is retouched | - |
|  |  |  | PartiallyBacked | Partially backed. Lateral edge is only partially backed |  |
| Other Notes | OtherNotes |  | Enter Other Notes | - | - |

**Notes regarding calculations for analyses**

Indeterminate category always excluded from calculations.

Some categories lumped for analysis. See Category column in Table D in S1 Dataset.

Edge length (EL) and EL/Mass were calculated according to Mackay (2008)

Flake Platform Area Estimate was calculated multiplying platform width by platform thicknes.

Surface Area Estimate, Cortical Surface Area Estimate, and PreHeat Surface Area Estimate were calculated according to Douglass et al. (2008).

**References**

Andrefsky, W., 2005. Lithics: Macroscopic approaches to analysis. Cambridge University Press.

Boëda, E., 1995. Levallois: A Volumetric Construction, Methods, a Technique, The Definition and Interpretation of Levallois Technology. Prehistory Press, Madison, pp. 41-68.

Brown, K.S., 2011. The Sword in the Stone: lithic raw material exploitation in the Middle Stone Age at Pinnacle Point Site 5-6, Southern Cape, South Africa, Unpublished Dissertation. University of Cape Town, Cape Town.

Brown, K.S., Marean, C.W., Herries, A.I.R., Jacobs, Z., Tribolo, C., Braun, D., Roberts, D.L., Meyer, M.C., Bernatchez, J., 2009. Fire As an Engineering Tool of Early Modern Humans. Science 325, 859-862.

Conard, N.J., Soressi, M., Parkington, J.E., Wurz, S., Yates, R., 2004. A unified lithic taxonomy based on patterns of core reduction. South African Archaeological Bulletin 59, 13-17.

Debénath, A., Dibble, H.L., 1994. Handbook of Paleolithic Typology: Lower and Middle Paleolithic of Europe. UPenn Museum of Archaeology.

Dibble, H.L., 1997. Platform variability and flake morphology: a comparison of experimental and archaeological data and implications for interpreting prehistoric lithic technological strategies. Lithic Technology, 150-170.

Douglass, M.J., Simon, J.H., Fanning, P.C., Shiner, J.I., 2008. An assessment and archaeological application of cortex measurement in lithic assemblages. American Antiquity 73, 513-526.

Fischer, A., Vemming Hansen, P., Rasmussen, P., 1984. Macro and micro wear traces on lithic projectile points: experimental results and prehistoric examples. Journal of Danish Archaeology 3, 19-46.

Inizan, M.L., Reduron-Ballinger, M., Roche, H., Tixier, J., 1999. Technology and Terminology of Knapped Stone. Nanterre:CREP.

Mackay, A., 2008. A method for estimating edge length from flake dimensions: Use and implications for technological change in the southern African MSA. Journal of Archaeological Science 35, 614-622.

Porraz, G., Texier, P.-J., Archer, W., Piboule, M., Rigaud, J.-P., Tribolo, C., 2013. Technological successions in the Middle Stone Age sequence of Diepkloof Rock Shelter, Western Cape, South Africa. Journal of Archaeological Science 40, 3376-3400.

Soriano, S., Villa, P., Wadley, L., 2007. Blade technology and tool forms in the Middle Stone Age of South Africa: the Howiesons Poort and post-Howiesons Poort at Rose Cottage Cave. Journal of Archaeological Science 34, 681-703.

Villa, P., Delagnes, A., Wadley, L., 2005. A late Middle Stone Age artifact assemblage from Sibudu (KwaZulu-Natal): comparisons with the European Middle Paleolithic. Journal of Archaeological Science 32, 399-422.

Villa, P., Soriano, S., Teyssandier, N., Wurz, S., 2010. The Howiesons Poort and MSA III at Klasies River main site, Cave 1A. Journal of Archaeological Science 37, 630-655.

Villa, P., Soriano, S., Tsanova, T., Degano, I., Higham, T.F.G., d’Errico, F., Backwell, L., Lucejko, J.J., Colombini, M.P., Beaumont, P.B., 2012. Border Cave and the beginning of the Later Stone Age in South Africa. Proceedings of the National Academy of Sciences 109, 13208-13213.

Volman, T.P., 1981. The Middle Stone Age in the Southern Cape. Unpublished Ph.D. dissertation, University of Chicago.

Wilkins, J., 2013. Technological Change in the Early Middle Pleistocene: The Onset of the Middle Stone Age at Kathu Pan 1, Northern Cape, South Africa. University of Toronto, Toronto.

**Code for CFG file to use with E4 (**[www.oldstoneage.com](http://www.oldstoneage.com)**)**

[E4]

Filename=REPLACEFILENAMEHERE.mdb

Delaytime=1

Table=REPLACETABLENAMEHERE

Re-edit=Yes

BackColor=13619151

[PlottedFindNumber]

Type=Instrument

Prompt=Enter Plotted Find Number:

Length=6

Unique=True

[Lot]

Type=Numeric

Prompt=Enter the Lot Number:

Length=6

Carry=True

[ExcavatedWithResidueProto]

Type=Menu

Prompt=Was Artifact Excavated with Residue Protocol?:

Menu=No Yes

Length=5

Carry=True

[Washed]

Type=Menu

Prompt=Was Artifact Washed?:

Menu=No Yes

Length=5

Carry=True

[Researcher]

Type=Menu

Prompt=Select the Researcher:

Menu=REPLACE RESEARCHER NAMES HERE

Length=30

Carry=True

[RawMaterial]

Type=Menu

Prompt=Select the Raw Material:

Menu=REPLACE SITE SPECIFIC RAW MATERIAL CODES HERE

Length=25

Carry=True

[OtherRaw MaterialID]

Type=Text

Prompt=Identify or Describe the Raw Material:

Length=50

Condition1=RawMaterial Other

[LithicArtifactClass]

Type=Menu

Prompt=Select the Lithic Artifact Class:

Menu=CompFlake FlakeFrag BladeBladeFrag Shatter RetouchedPiece Core HammerManuportGrindstone

Length=25

[UnretouchedPoint?]

Type=Menu

Prompt=Is this piece a Point or Point Fragment?:

Menu=No Yes Indeterminate

Length=15

Condition1=LithicArtifactClass CompFlake FlakeFrag BladeBladeFrag

[RetouchedPieceBlank]

Type=Menu

Prompt=Enter the probable Blank for the Retouched Piece:

Menu=Flake Blade ShatterIndeterminate

Length=20

Condition1=LithicArtifactClass RetouchedPiece

[Completeness]

Type=Menu

Prompt=Enter the Portion of the Artifact that is Present. For Retouched Pieces Record the Blank:

Menu=Complete Proximal Fragment Distal Mesial LeftLateral RightLateral ProxLeftLat ProxRightLat DistalLeftLat DistalRightLat MesialLeftLat MesialRightLat

Length=25

Condition1=LithicArtifactClass NOT CompFlake Shatter Core HammerManuportGrindstone

[CoreHammerCompleteness]

Type=Menu

Prompt=Select completeness of Core or HammerManuportGrindstone:

Menu=Complete SplitFragment

Length=20

Condition1=LithicArtifactClass Core HammerManuportGrindstone

[EvidenceBipolarPercussion]

Type=Menu

Prompt=Is there Evidence for Bipolar (Hammer/Anvil) Percussion?:

Menu=No Yes Indeterminate

Length=15

Condition1=LithicArtifactClass CompFlake Core HammerManuportGrindstone OR

Condition2=Completeness Complete LeftLateral RightLateral SplitHammerCobble

[TechnicalCategory]

Type=Text

Prompt=Enter the Sub-Agg Specific Technical Category:

Length=25

Condition1=LithicArtifactClass BladeBladeFrag

[CortexArea]

Type=Menu

Prompt=Enter the Estimated Cortex Area of DORSAL surface, or the entire surface of Shatter Core and HammerManuportGrindstone:

Menu=0% 1-20% 21-40% 41-60% 61-80% 81-99% 100% Indeterminate

Length=13

[PlatformCortex]

Type=Menu

Prompt=Is there Platform Cortex?:

Menu=No YesComplete YesPartial Indeterminate

Length=20

Condition1=LithicArtifactClass CompFlake OR

Condition2=Completeness Complete Proximal LeftLateral RightLateral ProxLeftLat ProxRightLat

[CortexChatterMarks]

Type=Menu

Prompt=Select whether there are Chatter Marks present, or not:

Menu=No Yes Indeterminate

Length=20

Condition1=CortexArea NOT 0% OR

Condition2=PlatformCortex YesComplete YesPartial

[CortexRoundness]

Type=Menu

Prompt=Select the Roundness of the Cortex Edges:

Menu=Angular SubangularSubrounded Rounded Indeterminate

Length=20

Condition1=CortexArea NOT 0% OR

Condition2=PlatformCortex YesComplete YesPartial

[CortexLocation]

Type=Menu

Prompt=Select where the Majority of Cortex is Located:

Menu=WholeDorsal Proximal Distal Mesial LeftLateral RightLateral ProxLeftLat ProxRightLat DistalLeftLat DistalRightLat MesialLeftLat MesialRightLat Indeterminate

Length=20

Condition1=CortexArea NOT 0% AND

Condition2=LithicArtifactClass NOT Shatter Core HammerManuportGrindstone

[CortexType]

Type=Menu

Prompt=Select the probable Cortex Type:

Menu=Cobble Outcrop Ind.

Length=25

Condition1=CortexArea NOT 0% OR

Condition2=PlatformCortex YesComplete YesPartial

[VisibleLuster]

Type=Menu

Prompt=Is there a Visible Heat-treatment Luster?:

Menu=Yes No Indeterminate

Length=20

Condition1=RawMaterial Silcrete

[PreHeatArea]

Type=Menu

Prompt=Enter the Estimated PreHeatTreatment Area of DORSAL surface, or the entire surface of Shatter Core and HammerManuportGrindstone:

Menu=0% 1-20% 21-40% 41-60% 61-80% 81-99% 100% Indeterminate

Length=13

Condition1=RawMaterial Silcrete AND

Condition2=VisibleLuster Yes

[PlatformPreHeatSurface]

Type=Menu

Prompt=Is there a PreHeatTreatment Surface on the Platform?:

Menu=No YesComplete YesPartial Indeterminate

Length=20

Condition1=RawMaterial Silcrete AND

Condition2=VisibleLuster Yes AND

Condition3=LithicArtifactClass CompFlake OR

Condition4=Completeness Complete Proximal LeftLateral RightLateral ProxLeftLat ProxRightLat

[PreHeatTreatmentLocation]

Type=Menu

Prompt=Select where the Majority of PreHeatTreatment Surface is Located:

Menu=WholeDorsal Proximal Distal Mesial LeftLateral RightLateral ProxLeftLat ProxRightLat DistalLeftLat DistalRightLat MesialLeftLat MesialRightLat Indeterminate

Length=20

Condition1=RawMaterial Silcrete AND

Condition2=VisibleLuster Yes AND

Condition3=PreHeatArea NOT 0% AND

Condition4=LithicArtifactClass NOT Shatter Core HammerManuportGrindstone

[EvidencePostDepBurning]

Type=Menu

Prompt=Select what kind of Evidence for Post-Depostional Burning is Present, if any:

Menu=None PotLidScar PieceISPotlid Crazing ColorChange CombinationOfFeatures

Length=21

[DorsalScarCount]

Type=Numeric

Prompt=Enter the Number of Dorsal Scars Greater than 6mm:

Length=5

Condition1=LithicArtifactClass NOT Shatter Core HammerManuportGrindstone AND

Condition2=CortexArea NOT 100%

[DorsalDirection]

Type=Menu

Prompt=Enter the Dorsal Scar Directionality:

Menu=Radial Subradial Bidirectional Unidirectional BiOrUni Indeterminate

Length=25

Condition1=LithicArtifactClass NOT Shatter Core HammerManuportGrindstone AND

Condition2=RetouchedPieceBlank NOT ShatterIndeterminate AND

Condition3=CortexArea NOT 100% AND

Condition4=DorsalScarCount NOT 0 1

[ArisOrientation]

Type=Menu

Prompt=Enter the Aris (Scar Ridge) Orientation:

Menu=Parallel Convergent Indeterminate

Length=13

Condition1=DorsalDirection Bidirectional Unidirectional BiOrUni AND

Condition2=CortexArea NOT 100% AND

Condition3=DorsalScarCount NOT 0 1 2

[ProfileShape]

Type=Menu

Prompt=Enter the Profile Shape:

Menu=Flat Curved Twisted Indeterminate

Length=13

Condition1=LithicArtifactClass CompFlake OR

Condition2=Completeness Complete Proximal LeftLateral RightLateral ProxLeftLateral ProxRightLat

[Mass]

Type=Numeric

Prompt=Enter the Mass (g):

Length=10

[MaxLength]

Type=Instrument

Prompt=Enter the Maximum Length (mm):

Length=10

[MaxWidth]

Type=Instrument

Prompt=Enter the Maximum Width (mm):

Length=10

[TechLength]

Type=Instrument

Prompt=Enter the Technological Length (mm):

Length=10

Condition1=LithicArtifactClass CompFlake OR

Condition2=Completeness Complete LeftLateral RightLateral

[MaxTechWidth]

Type=Instrument

Prompt=Enter the Max Technological Width (mm):

Length=10

Condition1=LithicArtifactClass CompFlake OR

Condition2=Completeness Complete Proximal Mesial

[MaxThickness]

Type=Instrument

Prompt=Enter the Max Thickness (mm):

Length=10

[MidThickness]

Type=Instrument

Prompt=Enter the Thickness at the Midpoint (mm):

Length=10

Condition1=LithicArtifactClass CompFlake OR

Condition2=Completeness Complete LeftLateral RightLateral

[PlatformWidth]

Type=Instrument

Prompt=Enter Width of the Platform (mm):

Length=10

Condition1=LithicArtifactClass CompFlake OR

Condition2=Completeness Complete Proximal

[PlatformThickness]

Type=Instrument

Prompt=Enter Thickness of the Platform (mm):

Length=10

Condition1=LithicArtifactClass CompFlake OR

Condition2=Completeness Complete Proximal LeftLateral RightLateral ProxLeftLat ProxRightLat

[ExteriorPlatAngle]

Type=Numeric

Prompt=Enter Exterior Platform Angle (degrees):

Length=10

Condition1=LithicArtifactClass CompFlake OR

Condition2=Completeness Complete Proximal LeftLateral RightLateral ProxLeftLat ProxRightLat

[FlakeTermination]

Type=Menu

Prompt=Select the Termination Type:

Menu=Feather Hinge Step HingeOrStep PartialHingeOrStep Overshoot Indeterminate

Length=25

Comment=Feather,acute angle, sharp

Comment=Hinge,curved up towards the dorsal surface

Comment=Step,abrupt right angle break

Comment=HingeOrStep,difficult to classify as Hinge or Step, but one of the two

Comment=PartialHingeOrStep,in profile, termination hinged or stepped on the ventral side and is feathered on the dorsal side

Comment=Overshoot,preserves core relict edge or opposite platform, plunging or not plunging

Comment=Indeterminate,difficult to classify or absent

Condition1=LithicArtifactClass CompFlake OR

Condition2=Completeness Complete Distal DistalLeftLat DistalRightLat

[PlatformPrep]

Type=Menu

Prompt=Select the Platform Preparation:

Menu=NotPrepared FacetedWithBulb ResidualFacetWithoutBulb Indeterminate

Length=35

Condition1=LithicArtifactClass CompFlake OR

Condition2=Completeness Complete Proximal LeftLateral RightLateral ProxLeftLat ProxRightLat AND

Condition3=PlatFormCortex NOT YesComplete

[NumberPlatformScars]

Type=Text

Prompt=Enter the Number of Platform Scars (greater than 1 mm):

Length=4

Condition1=LithicArtifactClass CompFlake OR

Condition2=Completeness Complete Proximal LeftLateral RightLateral ProxLeftLat ProxRightLat AND

Condition3=PlatformPrep NOT NotPrepared Indeterminate

[DorsalPrep]

Type=Menu

Prompt=Select the Type of DORSAL Preparation:

Menu=None HingedRemovals LongFeatherRemovals LateralNotching Indeterminate

Length=25

Condition1=LithicArtifactClass CompFlake OR

Condition2=Completeness Complete Proximal LeftLateral RightLateral ProxLeftLat ProxRightLat

[PlatAbrasion]

Type=Menu

Prompt=Is there Platform Abrasion visible?:

Menu=No Yes Indeterminate

Length=15

Condition1=LithicArtifactClass CompFlake OR

Condition2=Completeness Complete Proximal LeftLateral RightLateral ProxLeftLat ProxRightLat

[FractureInitiationPoint]

Type=Menu

Prompt=Where is the Fracture Initiation Point Located?:

Menu=NotExpressed Centered Lateral Indeterminate

Length=15

Condition1=LithicArtifactClass CompFlake OR

Condition2=Completeness Complete Proximal

[PlatformDelineation]

Type=Menu

Prompt=Select the Platform Delineation with the Ventral Surface?:

Menu=RegCurve OverhangCurveNoBreak OverhangCurveBreak Double Rectillinear Indeterminate

Length=20

Comment=RegCurve,platform on same plane as flake and bulb

Comment=OverhangCurveNoBreak,platform plane is more dorsal than rest of flake, delineation between platform and ventral surface is smooth

Comment=OverhangCurveBreak,delineation has a bump that sticks out where percussion point is

Comment=Double,there are two obvious percussion points

Comment=Rectillinear,delineation is not curved, but straight line

Comment=Indeterminate,difficult to classify

Condition1=LithicArtifactClass CompFlake OR

Condition2=Completeness Complete Proximal LeftLateral RightLateral ProxLeftLat ProxRightLat

[FissuringOnPlatform]

Type=Menu

Prompt=Select the Type of Fissuring, if any, on the Platform at the Percussion Point:

Menu=None CompleteFissure PartialFissure Indeterminate

Length=20

Condition1=LithicArtifactClass CompFlake OR

Condition2=Completeness Complete Proximal LeftLateral RightLateral ProxLeftLat ProxRightLat

[MarksVentralSurface]

Type=Menu

Prompt=Select the Type of Marks on the Ventral Surface, if any:

Menu=None RipplesUndulations ShatteredBulb HertzianCone Crushing Indeterminate

Length=18

Condition1=LithicArtifactClass CompFlake OR

Condition2=Completeness Complete Proximal LeftLateral RightLateral ProxLeftLat ProxRightLat

[Lipping]

Type=Menu

Prompt=Select the Size of Lip, if present:

Menu=NoLip SmallOrPartialLip LargeContinuousLip Indeterminate

Length=18

Condition1=LithicArtifactClass CompFlake OR

Condition2=Completeness Complete Proximal LeftLateral RightLateral ProxLeftLat ProxRightLat

[Bulb]

Type=Menu

Prompt=Is there a Bulb Visible?:

Menu=No Yes Indeterminate

Length=13

Condition1=LithicArtifactClass CompFlake OR

Condition2=Completeness Complete Proximal LeftLateral RightLateral ProxLeftLat ProxRightLat

[BulbLength]

Type=Instrument

Prompt=Enter the Length of the Bulb (mm):

Length=10

Condition1=Bulb Yes

[PlatformMorphology]

Type=Menu

Prompt=Select Platform Morphology:

Menu=NarrowLinear QuadrangularTrapezoid OvularOrTriangular NarrowCurved Punctiform ChapeauDeGendarme Indeterminate

Length=21

Condition1=LithicArtifactClass CompFlake OR

Condition2=Completeness Complete Proximal LeftLateral RightLateral ProxLeftLat ProxRightLat

[EdgeDamage]

Type=Menu

Prompt=Is Edge Damage Visible? For Retouched Pieces only look at Unmodified Edges.

Menu=No Yes Indeterminate

Length=15

Comment=No,edge shows no damage

Comment=Yes,small, generally isolated flake scars and/or snaps are present on edges, this includes DIFs, which will be coded next

Comment=Indeterminate,difficult to classify or determine

Condition1=LithicArtifactClass NOT Shatter Core HammerManuportGrindstone AND

Condition2=Completeness NOT Fragment

[DiagnosticImpactFractures]

Type=Menu

Prompt=What type of Diagnostic Impact Fractures, if any, are present?:

Menu=None StepTerminatingBendingFracture UnifacialSpinOff>6mm BifacialSpinOff ImpactBurinationNoBulb MoreThanOneTypeOfDIF

Length=30

Comment=None,No DIFS present, though there may be snaps or other types of breaks that are not 'diagnostic'

Comment=StepTerminatingBendingFracture,Step-terminating fractures end abruptly and meet the surface of the flake at a right angle (Hayden 1979). Bending fractures initiate without the formation of a Hertzian cone and consequently lack a negative bulb of percussion.

Comment=UnifacialSpinOff>6mm,Spin-off fractures are cone fractures that initiate off bending fractures. Unifacial spin-off fractures occur only on one face, not both. These fractures need to be more than 6 mm in max length to be considered DIFs (Fischer et al. 1984).

Comment=BifacialSpinOff, Multiple spin-off fractures that initiate off both faces of a bending fracture.

Comment=ImpactBurinationNoBulb,Impact burinations resemble a burin blow occurring along either one of the lateral edges, but lack the negative bulb of percussion common to deliberate burination.

Comment=MoreThanOneTypeOfDIF,More than one type of DIF is present. These will be coded later in a seperate analysis.

Condition1=EdgeDamage Yes

[BandingOrientation]

Type=Menu

Prompt=How is the Axis of Percussion Oriented with respect to Visible Banding, if present?

Menu=NoBandingVisible With Through Oblique Against Indeterminate

Length=16

Comment=NoBandingVisible,NoBandingVisible - no bands are visible

Comment=With,With - bands will be visible on dorsal and ventral surfaces and parallel to flaking axis

Comment=Through,Through - bands are mainly visible in side view and run parallel to the flaking axis

Comment=Oblique,Oblique - bands are visible on the dorsal and ventral surfaces and are diagonal to the flaking axis

Comment=Against,Against - bands are visible on dorsal and lateral surfaces and are perpendicular to flaking axis

Comment=Indeterminate,Indeterminate - banding visible but difficult to interpret relationship to flaking axis

Condition1=RawMaterial Quartzite AND

Condition2=LithicArtifactClass CompFlake OR

Condition3=Completeness Complete Proximal Distal LeftLateral RightLateral ProxLeftLat ProxRightLat DistalLeftLat DistalRightLat MesialLeftLat MesialRightLat

[RetouchType]

Type=Menu

Prompt=Select the Type of Retouch:

Menu=NotchDenticulate Backing Burination InvasiveEdgeShaping MarginalEdgeShaping PiecesEsquillees

Length=25

Comment=NotchDenticulate,one or more convex half-moon modification to edge that was prepared with at least two small flake removals, or denticulation is regular and exaggerated (snaps or single flake removals are not considered notches but should be coded as edge damage)

Comment=Backing,blunted edge(s) - very steep retouch, perpendicular to plane of tool

Comment=Burination,probably rare - must have negative bulb and located along a lateral edge

Comment=InvasiveEdgeShaping,Long flake scars extend into body of tool

Comment=MarginalEdgeShaping,Short flake scars that do not extend into body of tool

Comment=PiecesEsquillees,Wedge shaped piece with several small stepped scars imitating from two opposed chisel-like edges

Condition1=LithicArtifactClass RetouchedPiece

[RetouchEdgeAngle]

Type=Numeric

Prompt=Enter the Angle of the Retouched Edge. Measure center of longest series of continuous retouch or from inside the largest notch.:

Length=5

Condition1=RetouchType NotchDenticulate Backing InvasiveEdgeShaping MarginalEdgeShaping

[DiameterOfLargestNotch]

Type=Instrument

Prompt=Enter the Diameter of the Largest Notch (mm):

Length=5

Condition1=RetouchType NotchDenticulate

[RetouchedPieceTypology]

Type=Menu

Prompt=Select the Retouched Piece Typology:

Menu=BackedPiece NotchedPiece RetouchedPoint SideScraper EndScraper DoubleScraper ScraperWithThreeOrMoreEdges PiecesEsquillees MinimallyRetouched Indeterminate Other

Length=30

Comment=BackedPiece, steeply retouched edge, can be partial or complete, opposed edge 'cutting' edge is unretouched

Comment=NotchedPiece, notched or denticulated piece, each notch must have more than one retouch scar within it, an edge may be denitciluate with 'single flake notches' if there are in a continuous series and regular in form

Comment=RetouchedPoint,converging edges shaped into a point

Comment=SideScraper,continuous retouch along a portion of one lateral edge

Comment=EndScraper,continuous retouch that includes distal end

Comment=DoubleScraper,continuous retouch along two edges that do not converge or converge into a very thick or irregular point (would not be classified as a point)

Comment=PiecesEsquillees,Wedge shaped piece with several small stepped scars imitating from two opposed chisel-like edge

Comment=MinimallyRetouched,retouch is limited or edge damage may have resulted from use, difficult to classify because there is so little retouch

Comment=Indeterminate,difficult to classify because irregular or broken, but could be one of above categories

Comment=Other,does not fit above category, but a typological designation is possible (i.e., transverse scraper, tanged tool, any other thing that might surprise us), if you pick this you will have to call it something in the next field

Condition1=LithicArtifactClass RetouchedPiece

[OtherRetouchedPieceTypology]

Type=Text

Prompt=Type in Retouched Piece Typology:

Length=50

Condition1=RetouchedPieceTypology Other

[NumberCoreFaces]

Type=Menu

Prompt=Select the Number of Core Faces:

Menu=One Two Three Four MoreThanFour

Length=13

Comment=One,One face with one or more removals. Face 1 will be the largest and/or most exploited face.

Comment=Two,Two faces with one or more removals. Face 1 will be the largest and/or most exploited face. Face 2 will be the next largest and/or most exploited face.

Comment=Three,Three faces with one or more removals. Face 1 will be the largest and/or most exploited face. Face 2 will be the next largest and/or most exploited face. Face 3 will be the third largest and/or most exploited face.

Comment=Four,Four faces with one or more removals. Three faces with one or more removals. Face 1 will be the largest and/or most exploited face. Face 2 will be the next largest and/or most exploited face. Face 3 will be the third largest and/or most exploited face. Face 4 will be the fourth largest and/or most exploited face.

Comment=MoreThanFour,More than four faces with one or more removals. Details for only the first four faces will be recorded in this database.

Condition1=LithicArtifactClass Core

[Face1CortexArea]

Type=Menu

Prompt=Enter the Estimated Cortex Area of Face 1:

Menu=0% 1-20% 21-40% 41-60% 61-80% 81-99%

Length=10

Condition1=LithicArtifactClass Core AND

Condition2=CortexArea NOT 0%

[Face1PreHeatArea]

Type=Menu

Prompt=Enter the Estimated PreHeatTreatment Area of Face 1:

Menu=0% 1-20% 21-40% 41-60% 61-80% 81-99% 100%

Length=10

Condition1=LithicArtifactClass Core AND

Condition2=RawMaterial Silcrete AND

Condition3=VisibleLuster Yes AND

Condition4=PreHeatArea NOT 0%

[Face1DorsalScarCount]

Type=Numeric

Prompt=Enter the Number of Dorsal Scars Greater than 6mm on Face 1:

Length=5

Condition1=LithicArtifactClass Core

[Face1DorsalDirection]

Type=Menu

Prompt=Enter the Dorsal Scar Directionality of Face 1:

Menu=Radial Subradial Bidirectional Unidirectional BiOrUni Indeterminate NA

Length=25

Condition1=LithicArtifactClass Core AND

Condition2=Face1DorsalScarCount NOT 0 1

[Face1ArisOrientation]

Type=Menu

Prompt=Enter the Aris (Scar Ridge) Orientation of Face 1:

Menu=Parallel Convergent Indeterminate

Length=13

Condition1=LithicArtifactClass Core AND

Condition2=Face1DorsalDirection NOT Radial Subradial Indeterminate NA AND

Condition3=Face1DorsalScarCount NOT 0 1 2

[Face1CoreExploitation]

Type=Menu

Prompt=Enter the Type of Exploitation of Face 1:

Menu=Recurrent Preferential Platform Indeterminate

Length=20

Condition1=LithicArtifactClass Core

[Face1TerminationFinalRemoval]

Type=Menu

Prompt=Enter the Termination of the Final Removal on Face 1:

Menu=Feather Hinge Step HingeOrStep PartialHingeOrStep Overshoot Indeterminate

Length=25

Comment=Feather,acute angle, sharp

Comment=Hinge,curved up towards the dorsal surface

Comment=Step,abrupt right angle break

Comment=HingeOrStep,difficult to classify as Hinge or Step, but one of the two

Comment=PartialHingeOrStep,in profile, termination hinged or stepped on the ventral side and is feathered on the dorsal side

Comment=Overshoot,preserves core relict edge or opposite platform, plunging or not plunging

Comment=Indeterminate,difficult to classify or absent

Condition1=LithicArtifactClass Core

[Face1LengthOfFinalRemoval]

Type=Instrument

Prompt=Enter the Technological Length of the Final Removal on Face 1:

Length=5

Condition1=LithicArtifactClass Core

[Face1WidthOfFinalRemoval]

Type=Instrument

Prompt=Enter the Technological Width of the Final Removal on Face 1:

Length=5

Condition1=LithicArtifactClass Core

[Face1RemnantPlatformOfFinalRemoval]

Type=Menu

Prompt=Enter the Remnant Platform Type of the Final Removal on Face 1:

Menu=NotPrepared Faceted Cortical Indeterminate

Length=20

Condition1=LithicArtifactClass Core

[Face2CortexArea]

Type=Menu

Prompt=Enter the Estimated Cortex Area of Face 2:

Menu=0% 1-20% 21-40% 41-60% 61-80% 81-99%

Length=10

Condition1=LithicArtifactClass Core AND

Condition2=NumberCoreFaces NOT One AND

Condition3=CortexArea NOT 0%

[Face2PreHeatArea]

Type=Menu

Prompt=Enter the Estimated PreHeatTreatment Area of Face 2:

Menu=0% 1-20% 21-40% 41-60% 61-80% 81-99% 100%

Length=10

Condition1=LithicArtifactClass Core AND

Condition2=RawMaterial Silcrete AND

Condition3=VisibleLuster Yes AND

Condition4=PreHeatArea NOT 0% AND

Condition5=NumberCoreFaces NOT One

[Face2DorsalScarCount]

Type=Numeric

Prompt=Enter the Number of Dorsal Scars Greater than 6mm on Face 2:

Length=5

Condition1=LithicArtifactClass Core AND

Condition2=NumberCoreFaces NOT One

[Face2DorsalDirection]

Type=Menu

Prompt=Enter the Dorsal Scar Directionality of Face 2:

Menu=Radial Subradial Bidirectional Unidirectional BiOrUni Indeterminate NA

Length=25

Condition1=LithicArtifactClass Core AND

Condition2=Face2DorsalScarCount NOT 0 1 AND

Condition3=NumberCoreFaces NOT One

[Face2ArisOrientation]

Type=Menu

Prompt=Enter the Aris (Scar Ridge) Orientation of Face 2:

Menu=Parallel Convergent Indeterminate

Length=13

Condition1=LithicArtifactClass Core AND

Condition2=Face2DorsalDirection NOT Radial Subradial Indeterminate NA AND

Condition3=Face2DorsalScarCount NOT 0 1 2 AND

Condition4=NumberCoreFaces NOT One

[Face2CoreExploitation]

Type=Menu

Prompt=Enter the Type of Exploitation of Face 2:

Menu=Recurrent Preferential Platform Indeterminate

Length=20

Condition1=LithicArtifactClass Core AND

Condition2=NumberCoreFaces NOT One

[Face2TerminationFinalRemoval]

Type=Menu

Prompt=Enter the Termination of the Final Removal on Face 2:

Menu=Feather Hinge Step HingeOrStep PartialHingeOrStep Overshoot Indeterminate

Length=25

Comment=Feather,acute angle, sharp

Comment=Hinge,curved up towards the dorsal surface

Comment=Step,abrupt right angle break

Comment=HingeOrStep,difficult to classify as Hinge or Step, but one of the two

Comment=PartialHingeOrStep,in profile, termination hinged or stepped on the ventral side and is feathered on the dorsal side

Comment=Overshoot,preserves core relict edge or opposite platform, plunging or not plunging

Comment=Indeterminate,difficult to classify or absent

Condition1=LithicArtifactClass Core AND

Condition2=NumberCoreFaces NOT One

[Face2LengthOfFinalRemoval]

Type=Instrument

Prompt=Enter the Technological Length of the Final Removal on Face 2:

Length=5

Condition1=LithicArtifactClass Core AND

Condition2=NumberCoreFaces NOT One

[Face2WidthOfFinalRemoval]

Type=Instrument

Prompt=Enter the Technological Width of the Final Removal on Face 2:

Length=5

Condition1=LithicArtifactClass Core AND

Condition2=NumberCoreFaces NOT One

[Face2RemnantPlatformOfFinalRemoval]

Type=Menu

Prompt=Enter the Remnant Platform Type of the Final Removal on Face 2:

Menu=NotPrepared Faceted Cortical Indeterminate

Length=20

Condition1=LithicArtifactClass Core AND

Condition2=NumberCoreFaces NOT One

[Face3CortexArea]

Type=Menu

Prompt=Enter the Estimated Cortex Area of Face 3:

Menu=0% 1-20% 21-40% 41-60% 61-80% 81-99%

Length=10

Condition1=LithicArtifactClass Core AND

Condition2=NumberCoreFaces NOT One Two AND

Condition3=CortexArea NOT 0%

[Face3PreHeatArea]

Type=Menu

Prompt=Enter the Estimated PreHeatTreatment Area of Face 3:

Menu=0% 1-20% 21-40% 41-60% 61-80% 81-99% 100%

Length=10

Condition1=LithicArtifactClass Core AND

Condition2=RawMaterial Silcrete AND

Condition3=VisibleLuster Yes AND

Condition4=PreHeatArea NOT 0% AND

Condition5=NumberCoreFaces NOT One Two

[Face3DorsalScarCount]

Type=Numeric

Prompt=Enter the Number of Dorsal Scars Greater than 6mm on Face 3:

Length=5

Condition1=LithicArtifactClass Core AND

Condition2=NumberCoreFaces NOT One Two

[Face3DorsalDirection]

Type=Menu

Prompt=Enter the Dorsal Scar Directionality of Face 3:

Menu=Radial Subradial Bidirectional Unidirectional BiOrUni Indeterminate NA

Length=25

Condition1=LithicArtifactClass Core AND

Condition2=Face3DorsalScarCount NOT 0 1 AND

Condition3=NumberCoreFaces NOT One Two

[Face3ArisOrientation]

Type=Menu

Prompt=Enter the Aris (Scar Ridge) Orientation of Face 3:

Menu=Parallel Convergent Indeterminate

Length=13

Condition1=LithicArtifactClass Core AND

Condition2=Face3DorsalDirection NOT Radial Subradial Indeterminate NA AND

Condition3=Face3DorsalScarCount NOT 0 1 2 AND

Condition4=NumberCoreFaces NOT One Two

[Face3CoreExploitation]

Type=Menu

Prompt=Enter the Type of Exploitation of Face 3:

Menu=Recurrent Preferential Platform Indeterminate

Length=20

Condition1=LithicArtifactClass Core AND

Condition2=NumberCoreFaces NOT One Two

[Face3TerminationFinalRemoval]

Type=Menu

Prompt=Enter the Termination of the Final Removal on Face 3:

Menu=Feather Hinge Step HingeOrStep PartialHingeOrStep Overshoot Indeterminate

Length=25

Comment=Feather,acute angle, sharp

Comment=Hinge,curved up towards the dorsal surface

Comment=Step,abrupt right angle break

Comment=HingeOrStep,difficult to classify as Hinge or Step, but one of the two

Comment=PartialHingeOrStep,in profile, termination hinged or stepped on the ventral side and is feathered on the dorsal side

Comment=Overshoot,preserves core relict edge or opposite platform, plunging or not plunging

Comment=Indeterminate,difficult to classify or absent

Condition1=LithicArtifactClass Core AND

Condition2=NumberCoreFaces NOT One Two

[Face3LengthOfFinalRemoval]

Type=Instrument

Prompt=Enter the Technological Length of the Final Removal on Face 3:

Length=5

Condition1=LithicArtifactClass Core AND

Condition2=NumberCoreFaces NOT One Two

[Face3WidthOfFinalRemoval]

Type=Instrument

Prompt=Enter the Technological Width of the Final Removal on Face 3:

Length=5

Condition1=LithicArtifactClass Core AND

Condition2=NumberCoreFaces NOT One Two

[Face3RemnantPlatformOfFinalRemoval]

Type=Menu

Prompt=Enter the Remnant Platform Type of the Final Removal on Face 3:

Menu=NotPrepared Faceted Cortical Indeterminate

Length=20

Condition1=LithicArtifactClass Core AND

Condition2=NumberCoreFaces NOT One Two

[Face4CortexArea]

Type=Menu

Prompt=Enter the Estimated Cortex Area of Face 4:

Menu=0% 1-20% 21-40% 41-60% 61-80% 81-99%

Length=10

Condition1=LithicArtifactClass Core AND

Condition2=NumberCoreFaces NOT One Two Three AND

Condition3=CortexArea NOT 0%

[Face4PreHeatArea]

Type=Menu

Prompt=Enter the Estimated PreHeatTreatment Area of Face 4:

Menu=0% 1-20% 21-40% 41-60% 61-80% 81-99% 100%

Length=10

Condition1=LithicArtifactClass Core AND

Condition2=RawMaterial Silcrete AND

Condition3=VisibleLuster Yes AND

Condition4=PreHeatArea NOT 0% AND

Condition5=NumberCoreFaces NOT One Two Three

[Face4DorsalScarCount]

Type=Numeric

Prompt=Enter the Number of Dorsal Scars Greater than 6mm on Face 4:

Length=5

Condition1=LithicArtifactClass Core AND

Condition2=NumberCoreFaces NOT One Two Three

[Face4DorsalDirection]

Type=Menu

Prompt=Enter the Dorsal Scar Directionality of Face 4:

Menu=Radial Subradial Bidirectional Unidirectional BiOrUni Indeterminate NA

Length=25

Condition1=LithicArtifactClass Core AND

Condition2=Face4DorsalScarCount NOT 0 1 AND

Condition3=NumberCoreFaces NOT One Two Three

[Face4ArisOrientation]

Type=Menu

Prompt=Enter the Aris (Scar Ridge) Orientation of Face 4:

Menu=Parallel Convergent Indeterminate

Length=13

Condition1=LithicArtifactClass Core AND

Condition2=Face4DorsalDirection NOT Radial Subradial Indeterminate NA AND

Condition3=Face4DorsalScarCount NOT 0 1 2 AND

Condition4=NumberCoreFaces Not One Two Three

[Face4CoreExploitation]

Type=Menu

Prompt=Enter the Type of Exploitation of Face 4:

Menu=Recurrent Preferential Platform Indeterminate

Length=20

Condition1=LithicArtifactClass Core AND

Condition2=NumberCoreFaces NOT One Two Three

[Face4TerminationFinalRemoval]

Type=Menu

Prompt=Enter the Termination of the Final Removal on Face 4:

Menu=Feather Hinge Step HingeOrStep PartialHingeOrStep Overshoot Indeterminate

Length=25

Comment=Feather,acute angle, sharp

Comment=Hinge,curved up towards the dorsal surface

Comment=Step,abrupt right angle break

Comment=HingeOrStep,difficult to classify as Hinge or Step, but one of the two

Comment=PartialHingeOrStep,in profile, termination hinged or stepped on the ventral side and is feathered on the dorsal side

Comment=Overshoot,preserves core relict edge or opposite platform, plunging or not plunging

Comment=Indeterminate,difficult to classify or absent

Condition1=LithicArtifactClass Core AND

Condition2=NumberCoreFaces NOT One Two Three

[Face4LengthOfFinalRemoval]

Type=Instrument

Prompt=Enter the Technological Length of the Final Removal on Face 4:

Length=5

Condition1=LithicArtifactClass Core AND

Condition2=NumberCoreFaces NOT One Two Three

[Face4WidthOfFinalRemoval]

Type=Instrument

Prompt=Enter the Technological Width of the Final Removal on Face 4:

Length=5

Condition1=LithicArtifactClass Core AND

Condition2=NumberCoreFaces NOT One Two Three

[Face4RemnantPlatformOfFinalRemoval]

Type=Menu

Prompt=Enter the Remnant Platform Type of the Final Removal on Face 4:

Menu=NotPrepared Faceted Cortical Indeterminate

Length=20

Condition1=LithicArtifactClass Core AND

Condition2=NumberCoreFaces NOT One Two Three

[CoreBlankSphericity]

Type=Menu

Prompt=Select the Estimated Core Blank Sphericity:

Menu=NotSphericalFlat SphericalOrCubic Flake Indeterminate

Length=20

Condition1=LithicArtifactClass Core

[IntendedProduct]

Type=Menu

Prompt=Select the Intended Product:

Menu=Flake BladeBladelet Point Indeterminate

Length=25

Condition1=LithicArtifactClass Core

[VolmanTypeOne]

Type=Menu

Prompt=Select the Volman Type:

Menu=CoreOnFlake MinimalCore CorePrepForOneMajor CoreForIntersectingScars CoreForParallelConvScars CylinderCore Other

Length=25

Condition1=LithicArtifactClass Core

[VolmanCoreForIntersectingScars]

Type=Menu

Prompt=Select the Type of CoreForIntersectingScars:

Menu=ChangeOrientation RadialCore AdjacentPlatformCore

Length=25

Condition1=LithicArtifactClass Core AND

Condition2=VolmanTypeOne CoreForIntersectingScars

[VolmanCoreForParallelConvScars]

Type=Menu

Prompt=Select the Type of CoreForParallelConvScars:

Menu=SinglePlat OpposedPlatSameSide OpposedPlatOppSide OpposedPlatSameAndOppSide OtherDoublePlat

Length=25

Condition1=LithicArtifactClass Core AND

Condition2=VolmanTypeOne CoreForParallelConvScars

[ConardUnifiedType]

Type=Menu

Prompt=Select the Conard Unified Type:

Menu=Inclined Parallel Platform Other

Length=25

Condition1=LithicArtifactClass Core

[SiteSpecificCoreTypology]

Type=Menu

Prompt=Select the Site Specific Core Typology:

Menu=REPLACE SITE SPECIFIC CORE TYPLOGY HERE

Length=25

Condition1=LithicArtifactClass Core

[CompletenessOfBackedPiece]

Type=Menu

Prompt=Enter the completeness of the backed piece:

Menu=Complete Fragment

Length=25

Condition1=RetouchedPieceTypology BackedPiece

[BackedPieceTypology]

Type=Menu

Prompt=Select the backed piece typology:

Menu=Segment Triangle Trapeze TruncationSingle TruncationDouble TrunctionInd MinimallyBacked Indeterminate Other

Length=25

Condition1=RetouchedPieceTypology BackedPiece

[OtherBackedPieceID]

Type=Text

Prompt=Identify the other backed piece typology:

Length=50

Condition1=BackedPieceTypology Other

[BackingDistribution]

Type=Menu

Prompt=Enter how the backing retouch is distributed:

Menu=CompletelyBacked PartiallyBacked

Length=30

Condition1=RetouchedPieceTypology BackedPiece

[OtherNotes]

Type=Text

Prompt=Enter Other Notes:

Length=50
