# Supplementary material for: Lithic technological responses to Late Pleistocene glacial cycling at Pinnacle Point Site 5-6, South Africa
Source: PLoS One. 2017 Mar 29;12(3):e0174051. doi: 10.1371/journal.pone.0174051 (PMC5371328; doi:10.1371/journal.pone.0174051)
Supplement: S2 File — Correspondence plots for all multivariate categorical data that exhibited a significant association with MIS (Chi2, p<0.05). (PDF) [file pone.0174051.s002.pdf]

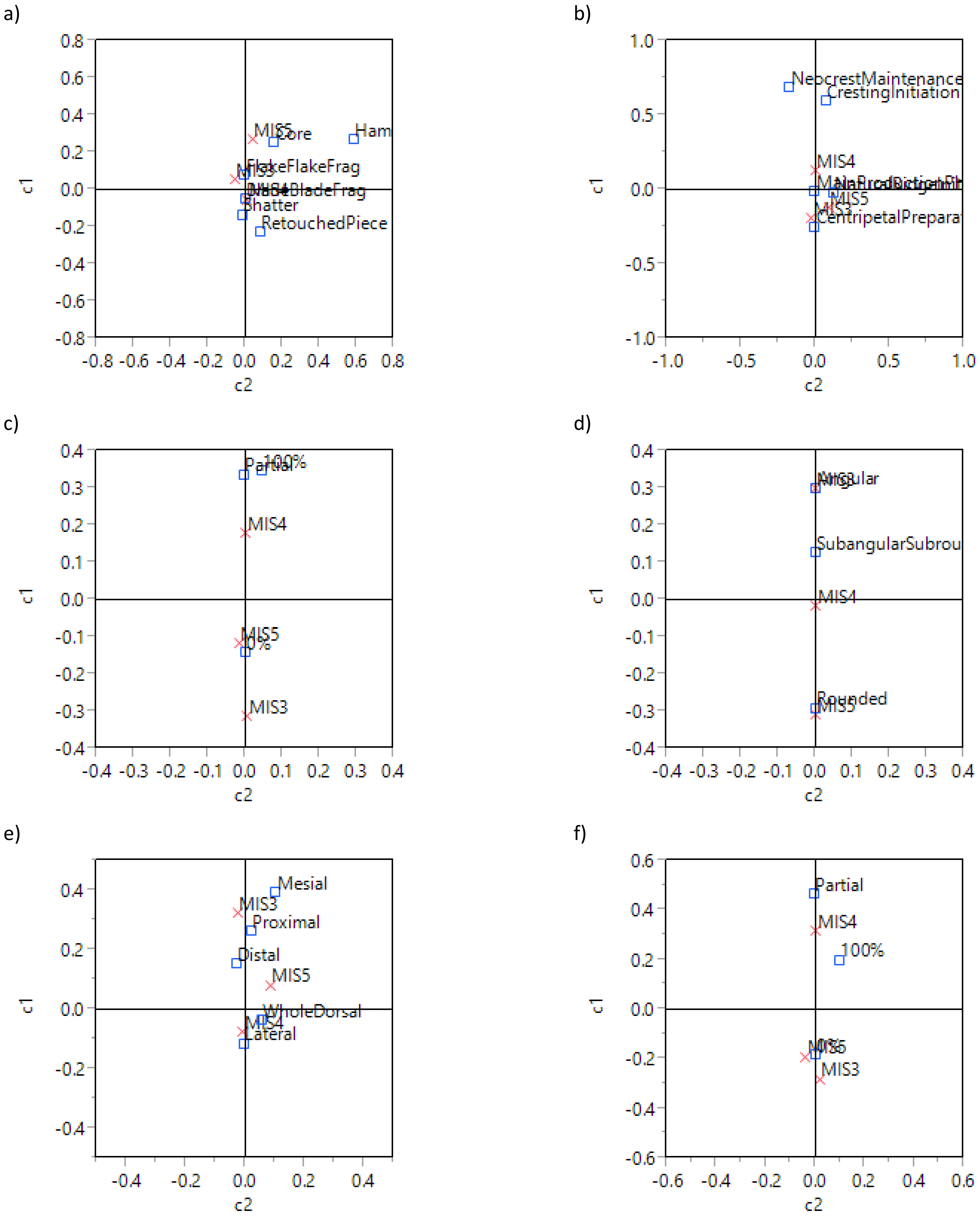

(figure caption on next page)

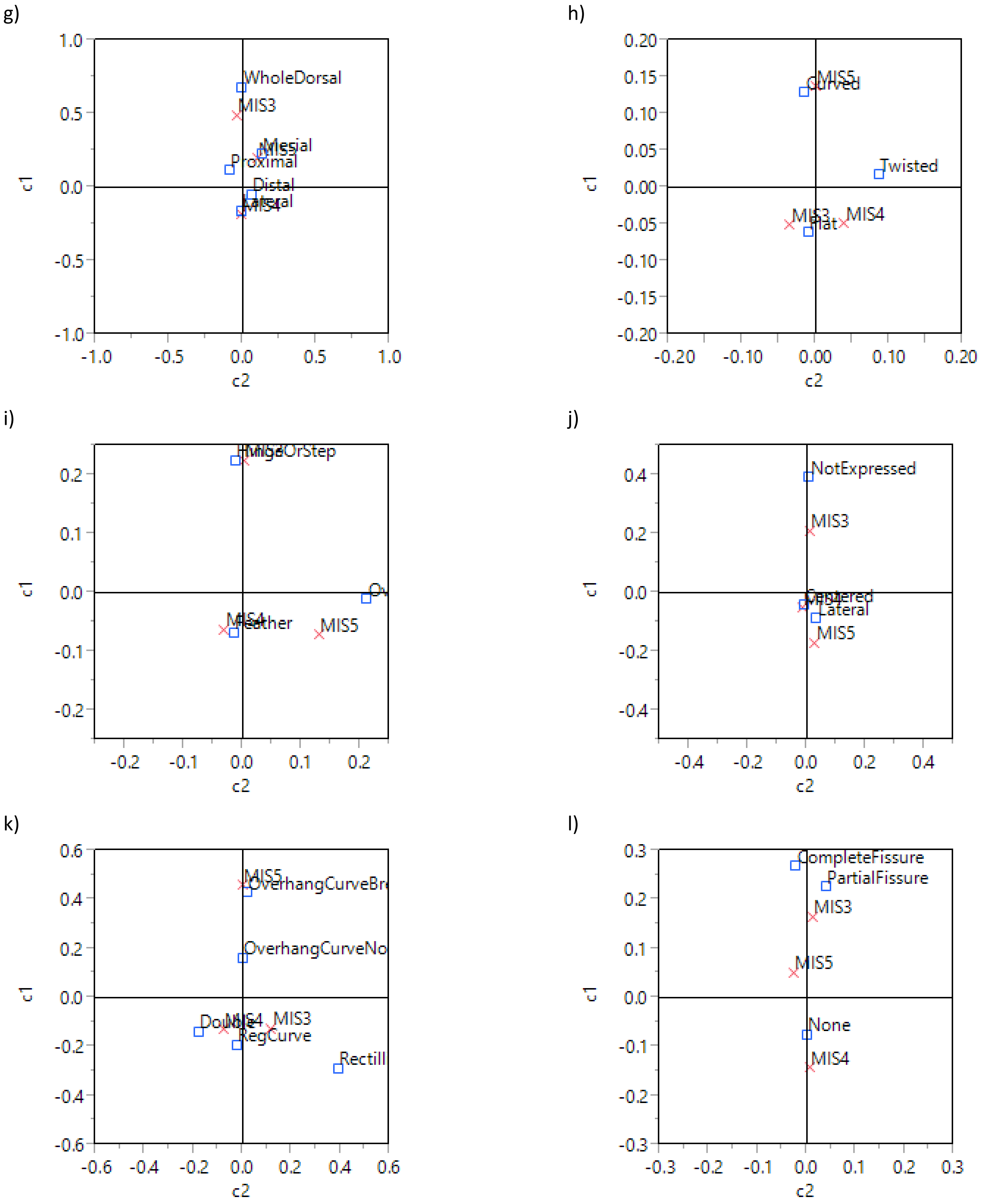

(figure caption on next page)

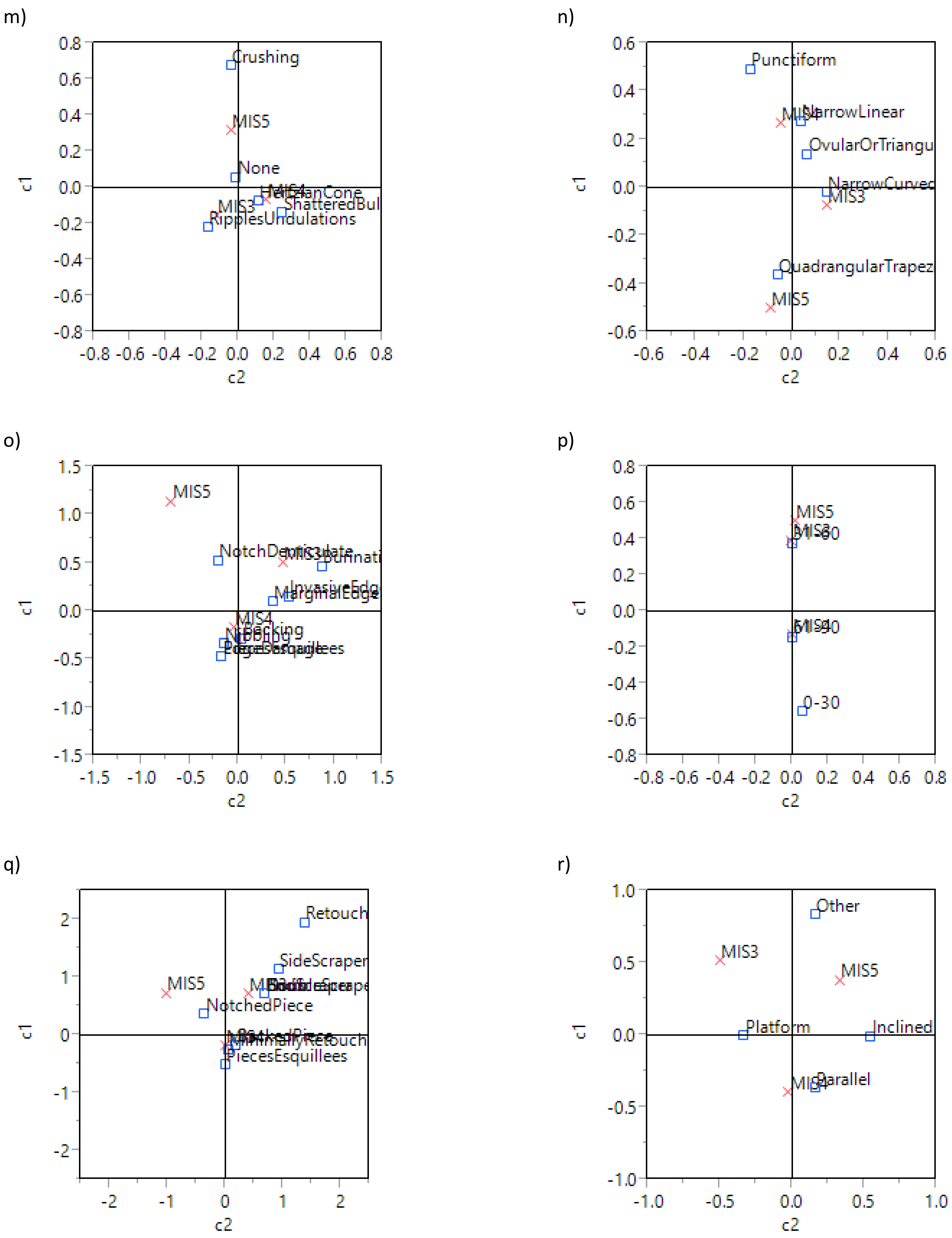

Fig A. Correspondence analysis plots for silcrete traits that show significant association with MIS. a) lithic artifact class, b)technical category, c)cortex area, d)cortex roundness, e)cortex location, f)pre heattreatment area, g)pre heattreatment location, h)profile shape, i) flake termination, j) fracture initiation point, k)platform delineation, l) fissuring on platform, m) marks on ventral surface, n) platform morphology, o)retouch type, p)retouch edge angle, q) retouched piece typology, r) core type Conard unified type. Further details in Tables F-H in S1 Dataset.

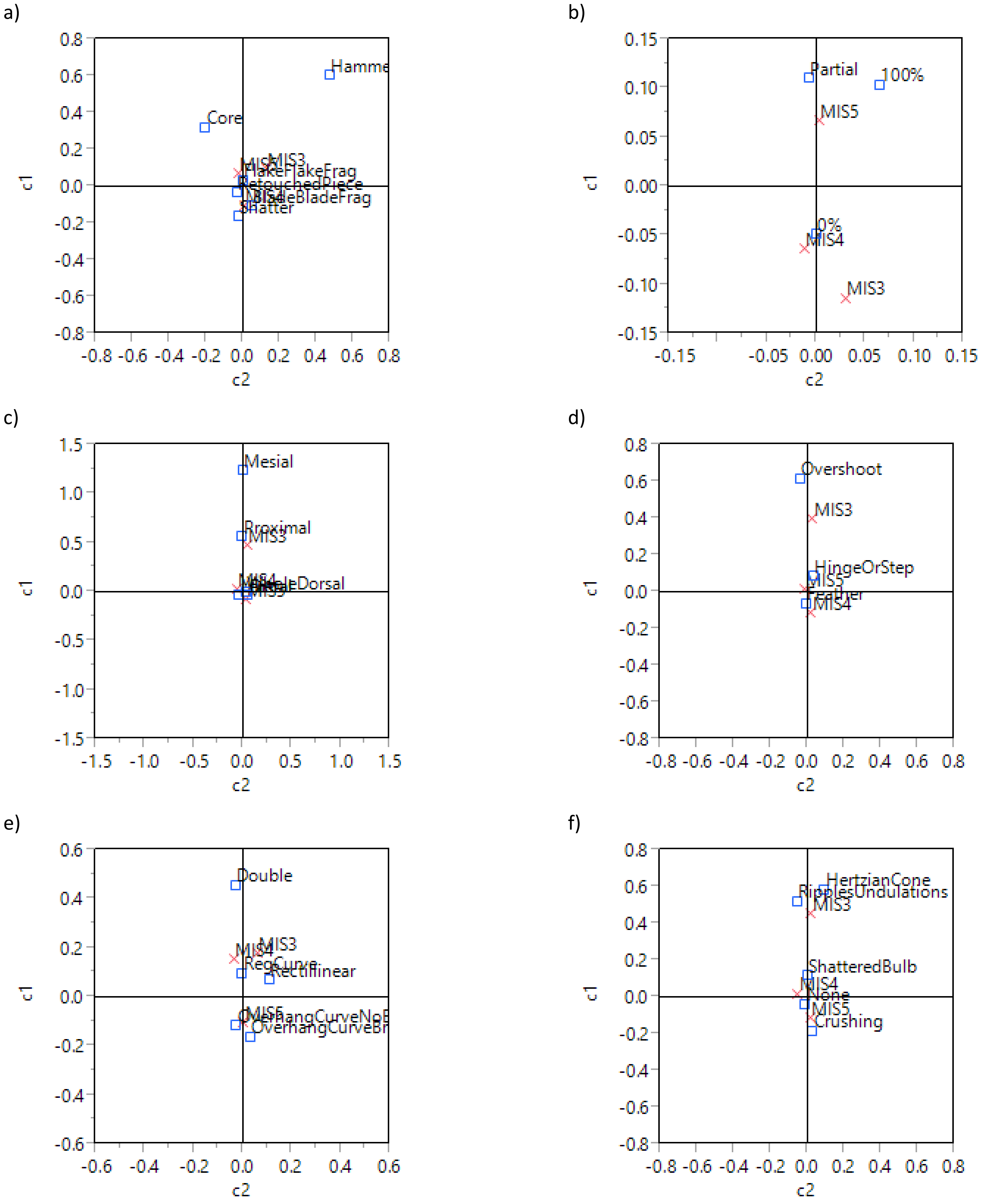

(figure caption on next page)

g)

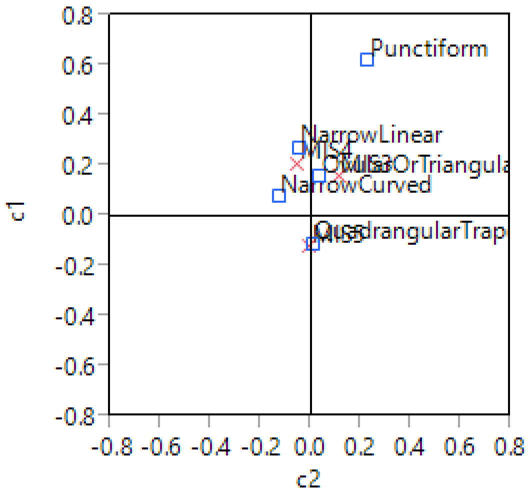

h)

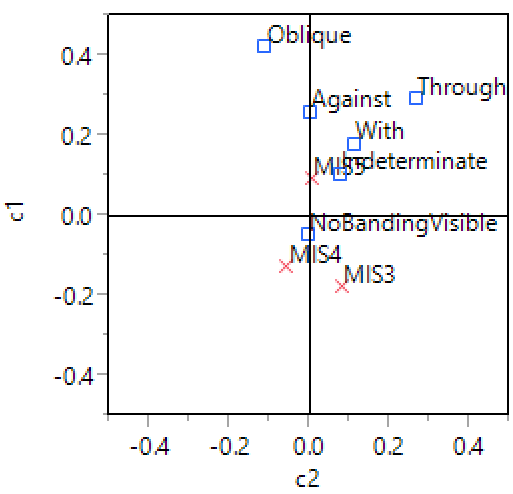

Fig B. Correspondence analysis plots for quartzite. a) lithic artifact class, b) cortex area, c) cortex location, d) flake termination, e)platform delineation, f)marks on ventral surface, g)platform morphology, h) banding orientation. Further details in Tables F-H in S1 Dataset.

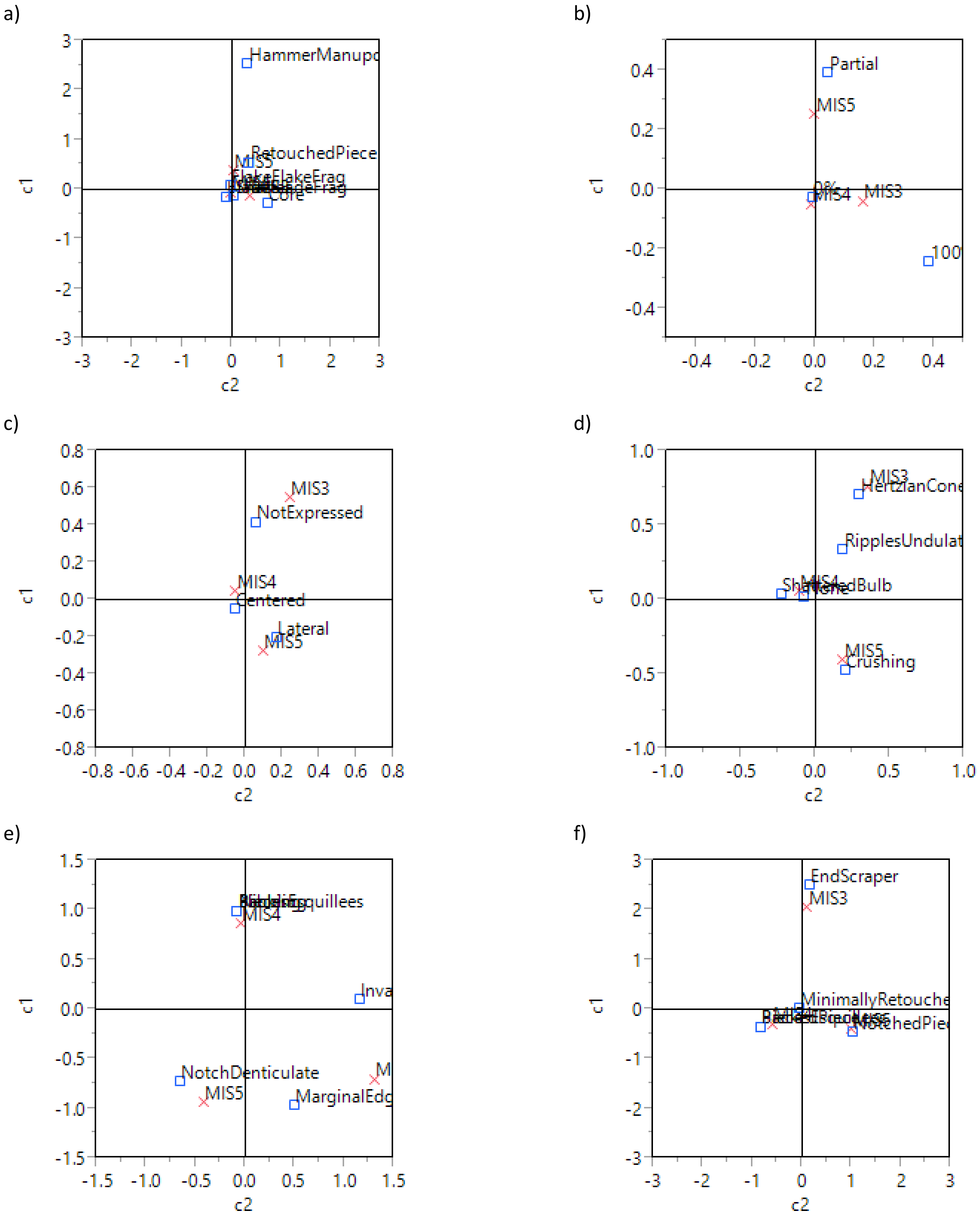

(figure caption on next page)

g)

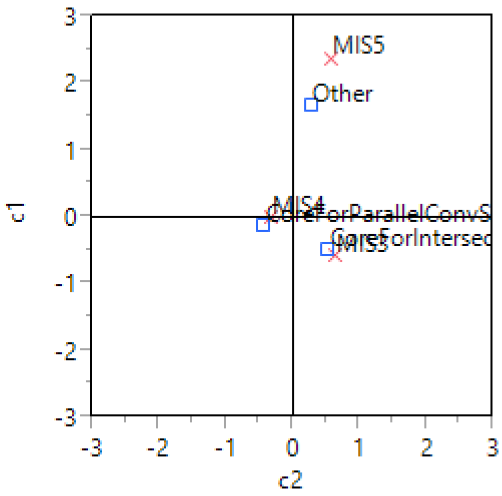

Fig C. Correspondence analysis plots for quartz. a) lithic artifact class, b) cortex area, c) fracture initiation point, d) marks on ventral surface, e) retouch type, f) retouched piece typology, g) Volman core type (CoreVolmanTypeOne). Further details in Tables F-H in S1 Dataset.

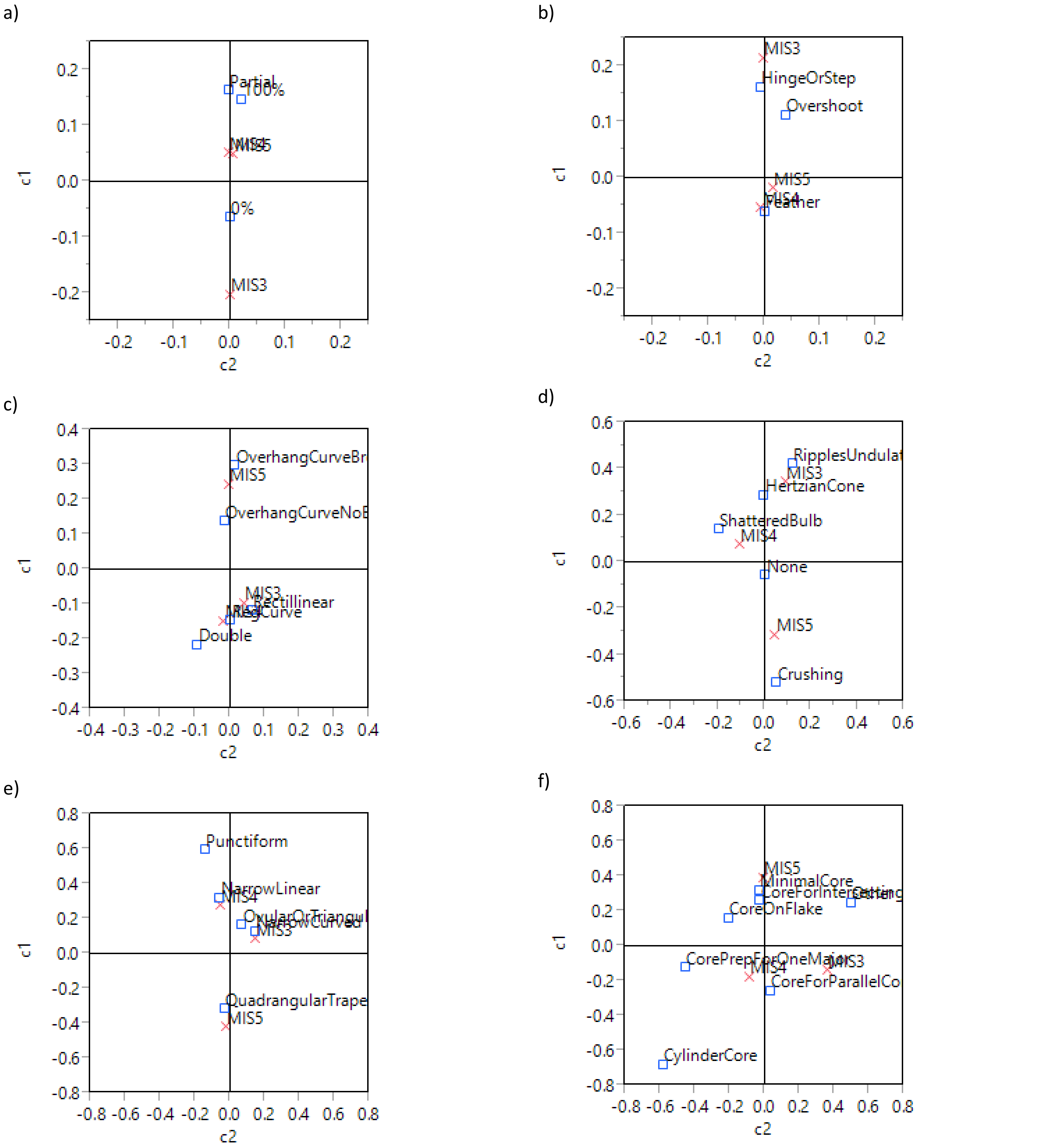

Fig D. Correspondence analysis plots for chert and chalcedony. a) cortex area, b)flake termination, c) platform delineation, d) platform delineation, e) platform morphology, f) Volman core type (CoreVolmanTypeOne). Further details in Tables F-H in S1 Dataset.
