# Supplementary material for: Lithic technological responses to Late Pleistocene glacial cycling at Pinnacle Point Site 5-6, South Africa
Source: PLoS One. 2017 Mar 29;12(3):e0174051. doi: 10.1371/journal.pone.0174051 (PMC5371328; doi:10.1371/journal.pone.0174051)
Supplement: S3 File — Relative point density maps for key traits based on point elevation. (PDF) [file pone.0174051.s003.pdf]

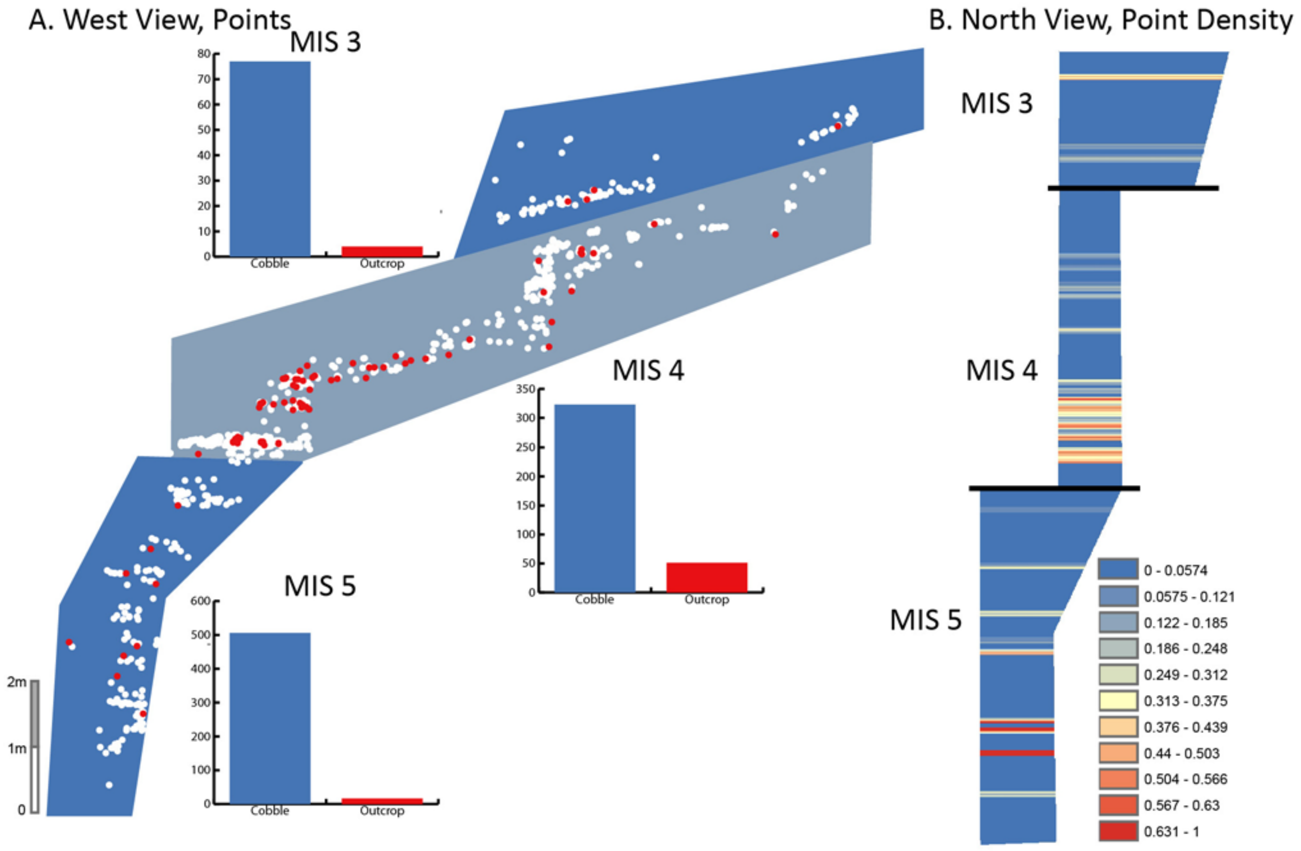

Figure E. Vertical distribution of quartzite cortex type frequencies at PP5-6. When lumped by MIS stage, relative quartzite outcrop cortex frequency is highest in MIS 4, though there is variability through the sequence. There is a significant association between quartzite cortex type and MIS (Pearson Chi^2=36.84, p<0.001). A. West view of PP5-6 lithic artifact plotted find points. MIS boundaries indicated by blue bounded areas are defined based on the mean OSL age estimate within StratAggs. Red points are quartzite artifacts with outcrop cortex, white points are quartzite artifacts with cobble cortex. Bar plots indicate frequency counts of quartzite cortex types and MIS. B. Relative point density map of quartzite artifacts with outcrop cortex for the north view of PP5-6. The relative point density map was generated in ArcMap using the rectangular neighborhood option and based on point elevation. Classes defined based on 1/3 standard deviation. Because some of the deposits dating to late MIS 4 and early MIS 3 overlap with respect to elevation, the MIS 3 relative density map was generated separately from the rest of the sequence and is depicted above the MIS 4 deposits in the point density column.

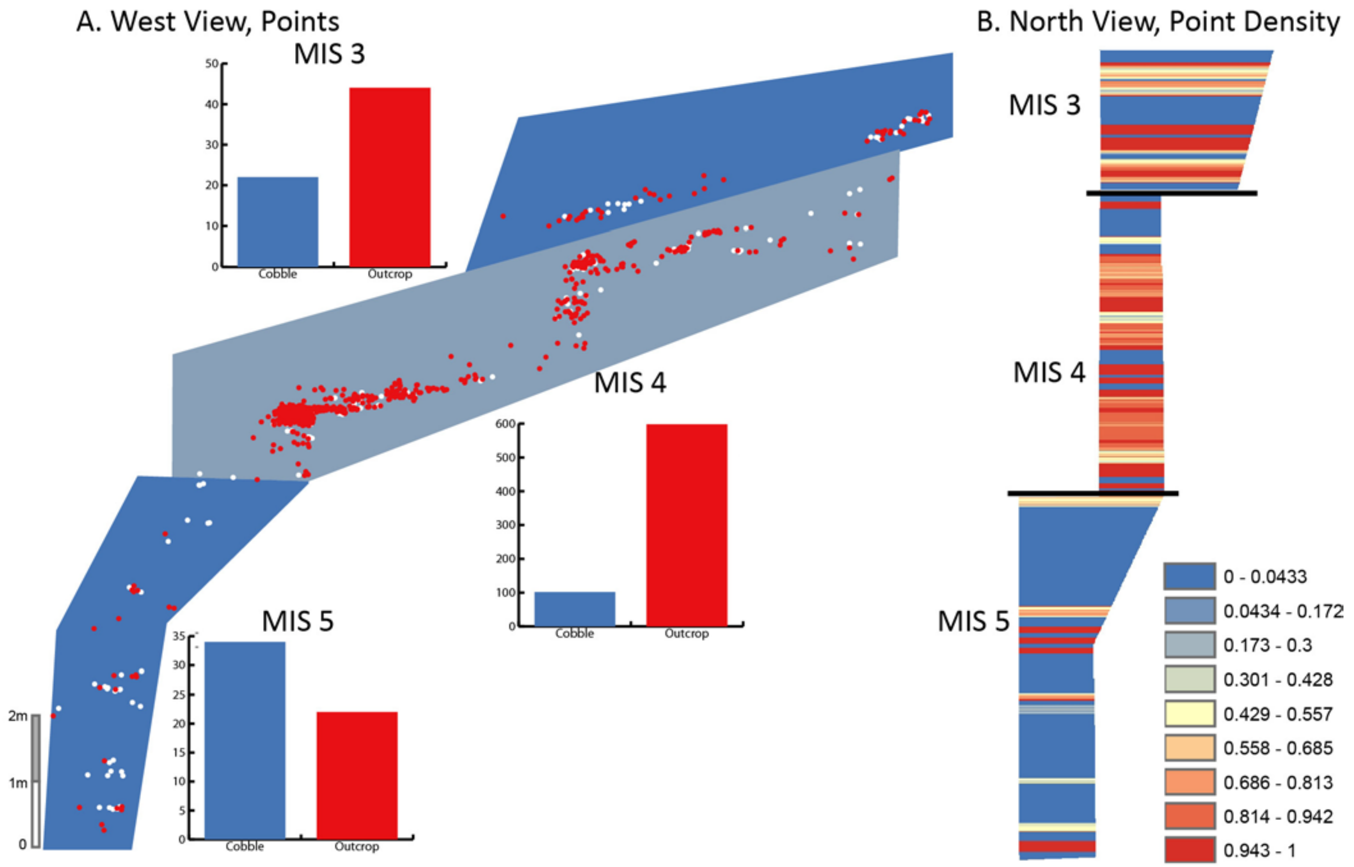

Figure F. Vertical distribution of silcrete cortex type frequencies at PP5-6. When lumped by MIS stage, relative silcrete outcrop cortex frequency is highest in MIS 4, though there is variability through the sequence. There is a significant association between silcrete cortex type and MIS (Pearson Chi^2=80.31, p<0.001). A. West view of PP5-6 lithic artifact plotted find points. MIS boundaries indicated by blue bounded areas are defined based on the mean OSL age estimate within StratAggs. Red points are silcrete artifacts with outcrop cortex, white points are silcrete artifacts with cobble cortex. Bar plots indicate frequency counts of silcrete cortex types and MIS. B. Relative point density map of silcrete artifacts with outcrop cortex for the north view of PP5-6. The relative point density map was generated in ArcMap using the rectangular neighborhood option and based on point elevation. Classes defined based on 1/3 standard deviation. Because some of the deposits dating to late MIS 4 and early MIS 3 overlap with respect to elevation, the MIS 3 relative density map was generated separately from the rest of the sequence and is depicted above the MIS 4 deposits in the point density column.

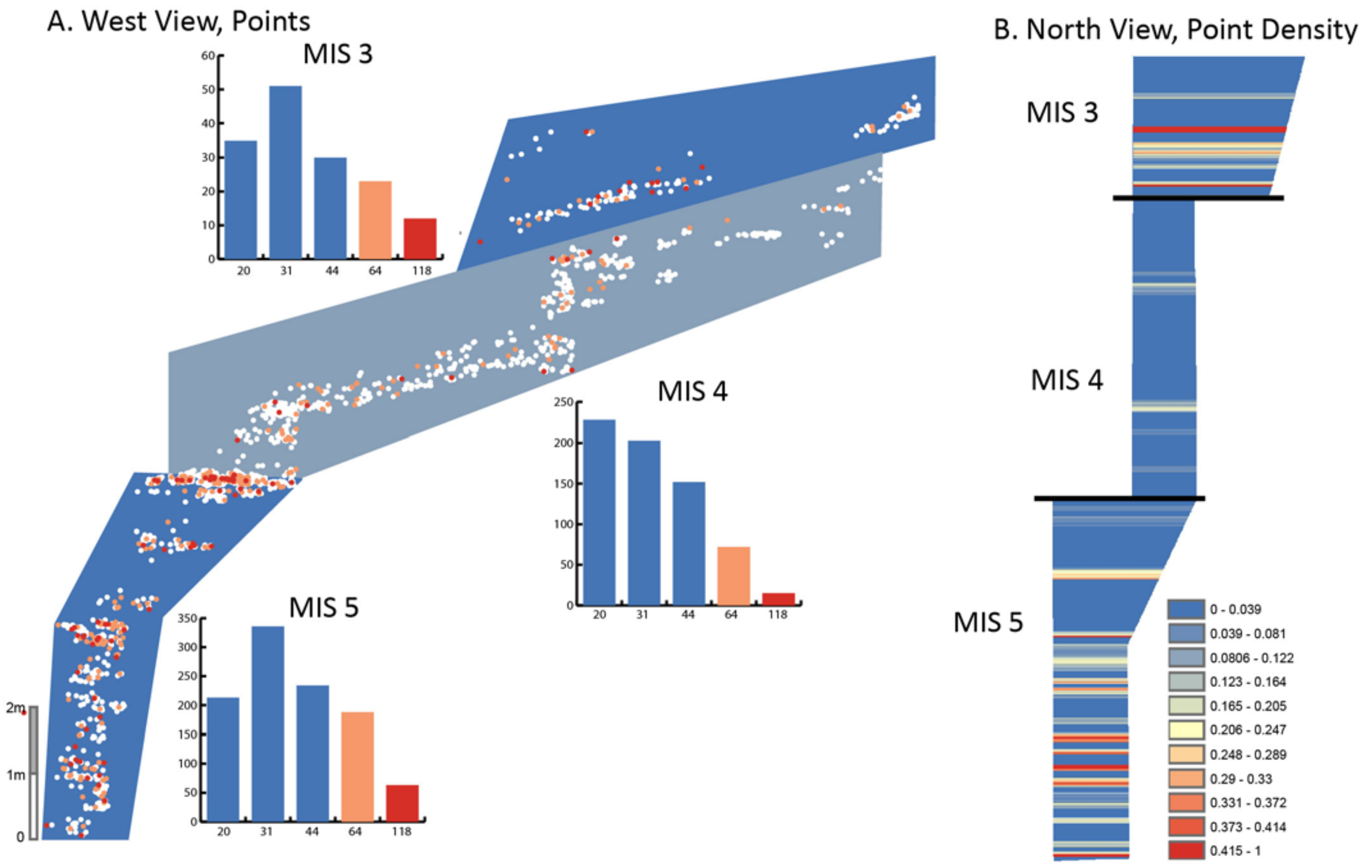

Figure G. Vertical distribution of quartzite technological length at PP5-6. When lumped by MIS stage, the frequency of quartzite pieces with high technological length values is lowest in MIS 4, though there is variability through the sequence. Mean quartzite technological length is significantly lower in MIS 4 (Wilcoxon Each Pair Test, $p<0.005$). A. West view of PP5-6 lithic artifact plotted find points. MIS boundaries indicated by blue bounded areas are defined based on the mean OSL age estimate within StratAggs. White points are in the three smallest classes, orange and red are in the second largest and largest classes, respectively. Classes defined based on Jenks optimization method. Bar plots indicate frequency counts of technological length classes; x-axis shows maximum value in that class. B. Relative point density map of north view of PP5-6 lithic artifact plotted finds with technological length values greater than 44 (top 2 classes) generated in ArcMap using the rectangular neighborhood option and based on point elevation. Classes defined based on 1/3 standard deviation. Because some of the deposits dating to late MIS 4 and early MIS 3 overlap with respect to elevation, the MIS 3 relative density map was generated separately from the rest of the sequence and is depicted above the MIS 4 deposits in the point density column.

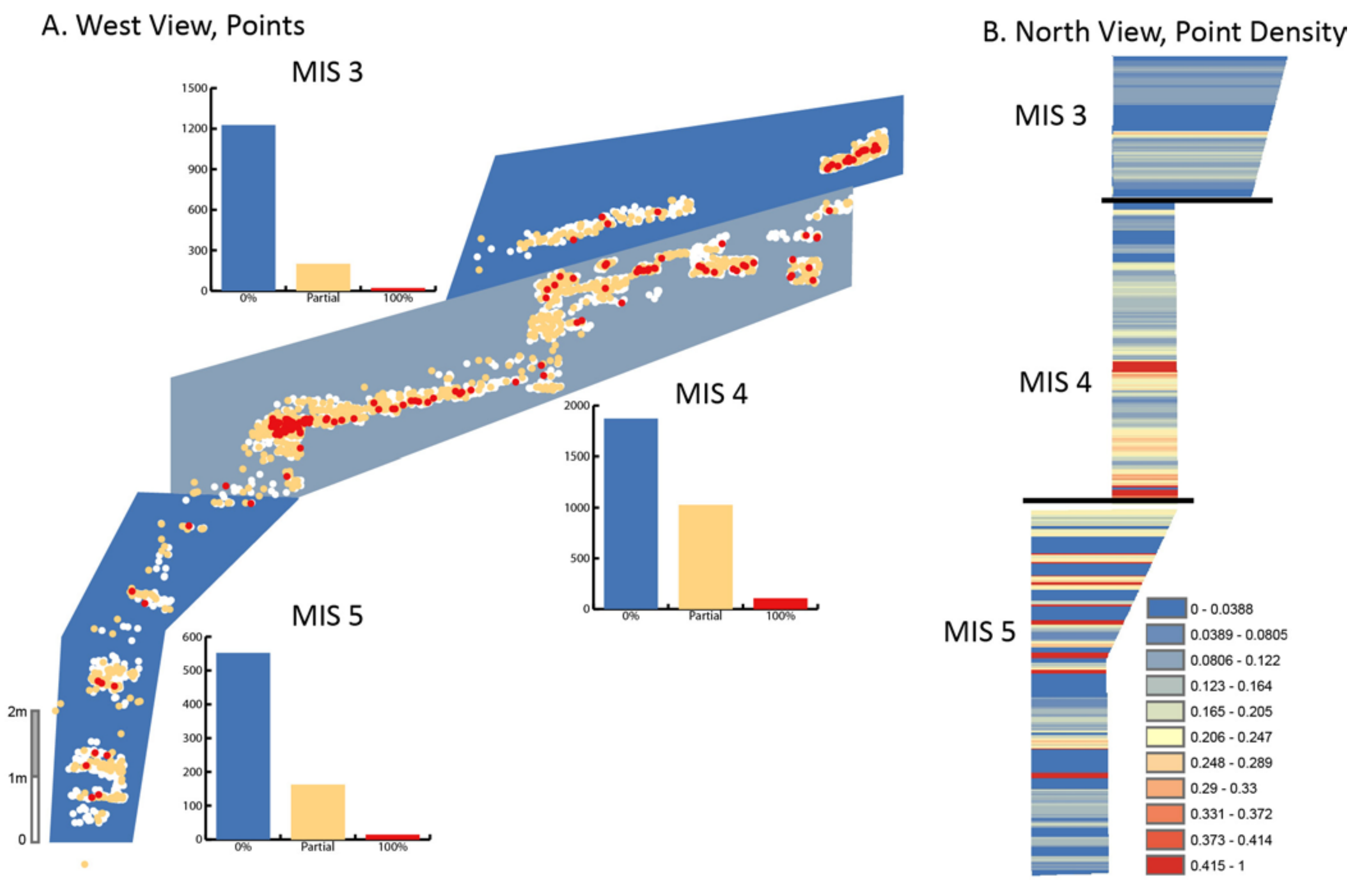

Figure H. Vertical distribution of silcrete cortex amount at PP5-6. When lumped by MIS stage, relative silcrete cortex amount is highest in MIS 4, though there is variability through the sequence. There is a significant association between silcrete cortex area and MIS (Pearson Chi^2=247.5, p<0.001). A. West view of PP5-6 lithic artifact plotted find points. MIS boundaries indicated by blue bounded areas are defined based on the mean OSL age estimate within StratAggs. Red points are silcrete artifacts with complete dorsal cortex, yellow points are silcrete artifacts with partial dorsal cortex, white points are silcrete artifacts with no cortex. Bar plots indicate frequency counts of the three silcrete cortex area classes and MIS. B. Relative point density map of north view of PP5-6 silcrete plotted finds with partial or complete cortex (last 2 classes) generated in ArcMap using the rectangular neighborhood option and based on point elevation. Classes defined based on 1/3 standard deviation. Because some of the deposits dating to late MIS 4 and early MIS 3 overlap with respect to elevation, the MIS 3 relative density map was generated separately from the rest of the sequence and is depicted above the MIS 4 deposits in the point density column.

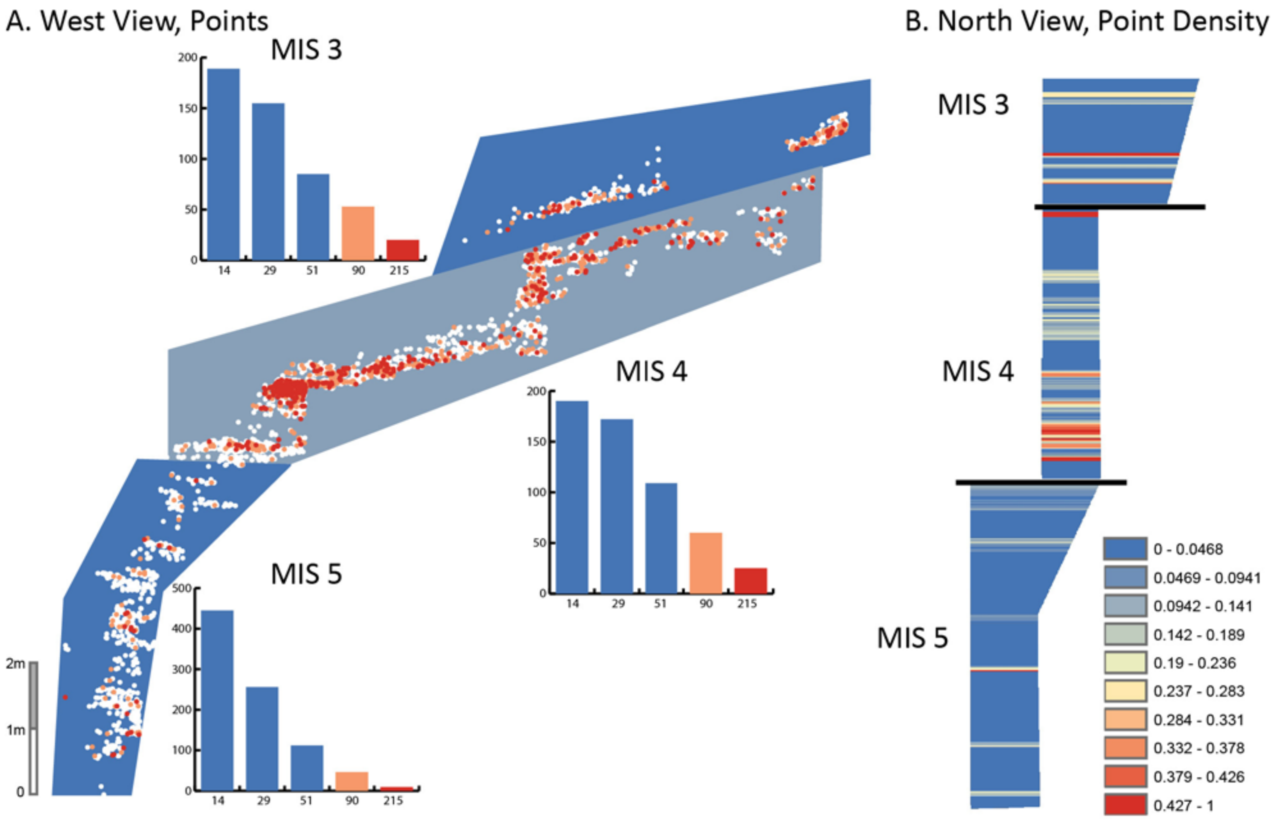

Figure I. Vertical distribution of quartzite edge length to mass ratio (EL/M) at PP5-6. When lumped by MIS stage, the frequency of quartzite pieces with high EL/M values is highest in MIS 4, though there is variability through the sequence. Mean quartzite EL/M is significantly higher in MIS 4 (Wilcoxon Each Pair Test, $p<0.001$). A. West view of PP5-6 lithic artifact plotted find points. MIS boundaries indicated by blue bounded areas are defined based on the mean OSL age estimate within StratAggs. White points are in the three smallest classes, orange and red are in the second largest and largest classes, respectively. Classes defined based on Jenks optimization method. Bar plots indicate frequency counts of EL/M classes; x-axis shows maximum value in that class. B. Relative point density map of north view of PP5-6 lithic artifact plotted finds with EL/M values greater than 51 (top 2 classes) generated in ArcMap using the rectangular neighborhood option and based on point elevation. Classes defined based on 1/3 standard deviation. Because some of the deposits dating to late MIS 4 and early MIS 3 overlap with respect to elevation, the MIS 3 relative density map was generated separately from the rest of the sequence and is depicted above the MIS 4 deposits in the point density column.

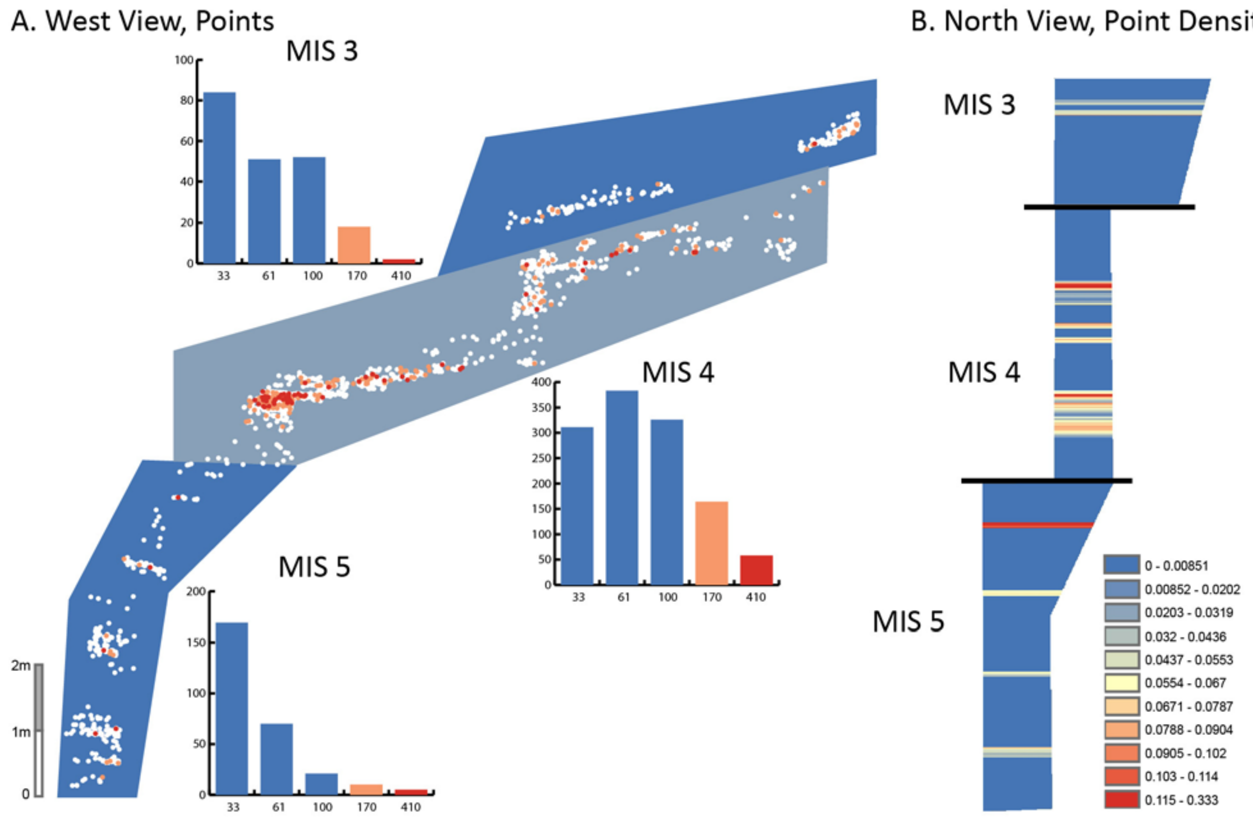

Figure J. Vertical distribution of silcrete edge length to mass ratio (EL/M) at PP5-6. When lumped by MIS stage, the frequency of silcrete pieces with high EL/M values is highest in MIS 4, though there is variability through the sequence. Mean silcrete EL/M is significantly higher in MIS 4 (Wilcoxon Each Pair Test, p<0.001). A. West view of PP5-6 lithic artifact plotted find points. MIS boundaries indicated by blue bounded areas are defined based on the mean OSL age estimate within StratAggs. White points are in the three smallest classes, orange and red are in the second largest and largest classes, respectively.. Classes defined based on Jenks optimization method. Bar plots indicate frequency counts of EL/M classes; x-axis shows maximum value in that class. B. Relative point density map of north view of PP5-6 lithic artifact plotted finds with EL/M values greater than 100 (top 2 classes) generated in ArcMap using the rectangular neighborhood option and based on point elevation. Classes defined based on 1/3 standard deviation. Because some of the deposits dating to late MIS 4 and early MIS 3 overlap with respect to elevation, the MIS 3 relative density map was generated separately from the rest of the sequence and is depicted above the MIS 4 deposits in the point density column.

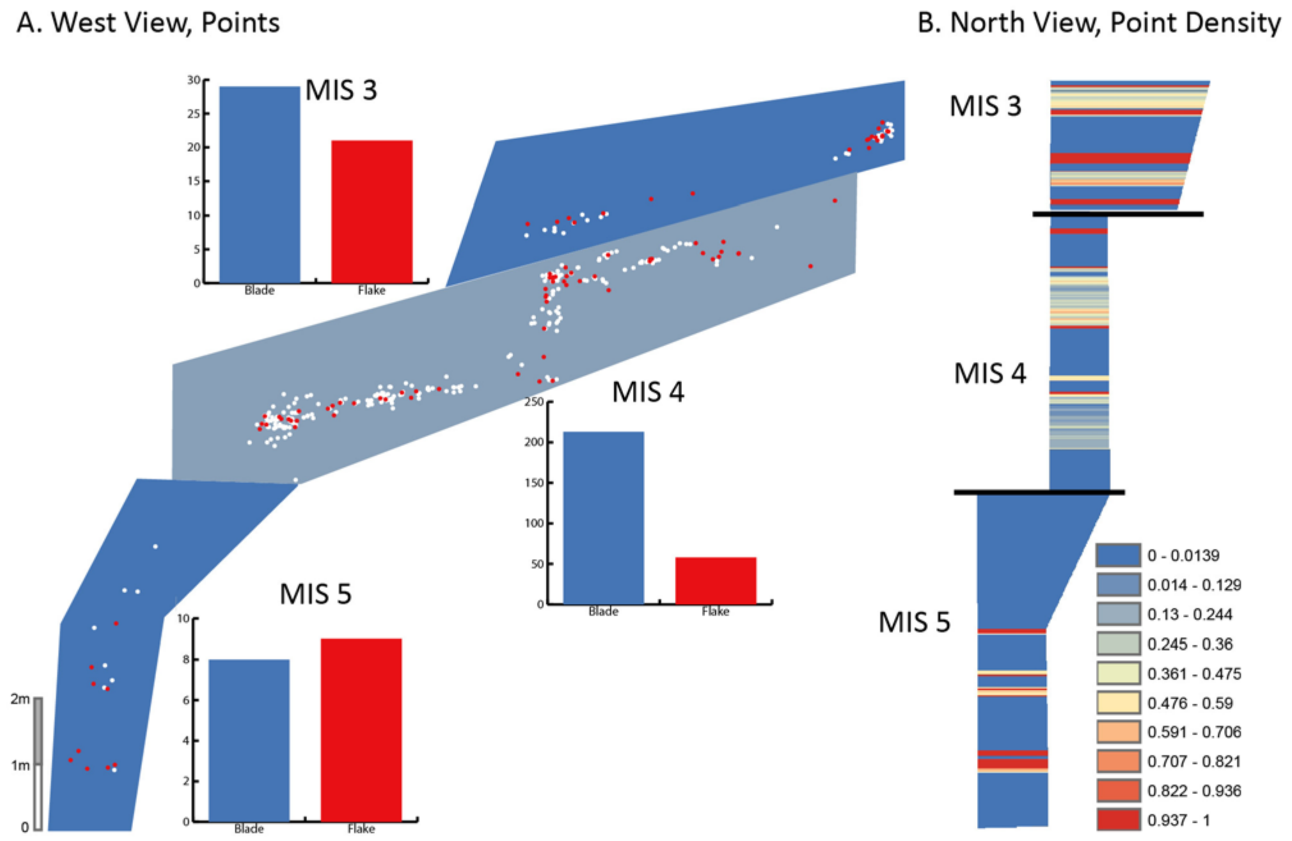

Figure K. Vertical distribution of silcrete retouched piece blank frequencies at PP5-6. When lumped by MIS stage, relative silcrete flake blank frequency is lowest in MIS 4, though there is variability through the sequence. There is a significant association between silcrete retouched piece blank and MIS (Pearson Chi^2=16.03, p<0.001). A. West view of PP5-6 lithic artifact plotted find points. MIS boundaries indicated by blue bounded areas are defined based on the mean OSL age estimate within StratAggs. Red points are silcrete retouched pieces on flake blanks, white points are silcrete retouched pieces on blade blanks. Bar plots indicate frequency counts of silcrete retouched piece blank types and MIS. B. Relative point density map of north view of PP5-6 silcrete retouched pieces on flake blanks generated in ArcMap using the rectangular neighborhood option and based on point elevation. Classes defined based on 1/3 standard deviation. Because some of the deposits dating to late MIS 4 and early MIS 3 overlap with respect to elevation, the MIS 3 relative density map was generated separately from the rest of the sequence and is depicted above the MIS 4 deposits in the point density column.
